# Electronic Coupling in Triferrocenylpnictogens

**DOI:** 10.1021/acsorginorgau.4c00034

**Published:** 2024-08-17

**Authors:** Corina Stoian, Fawaz Al Hussein, Wesley R. Browne, Emanuel Hupf, Jens Beckmann

**Affiliations:** † Institute for Inorganic Chemistry and Crystallography, Faculty of Biology and Chemistry, University of Bremen, Leobener Straße 7, Bremen 28359, Germany; ‡ Molecular Inorganic Chemistry, Stratingh Institute for Chemistry, Faculty of Science and Engineering, University of Groningen, Nijenborgh 4, Groningen 9747, AG, The Netherlands

**Keywords:** ferrocene, pnictogens, mixed valence species, spectroelectrochemistry, charge transfer

## Abstract

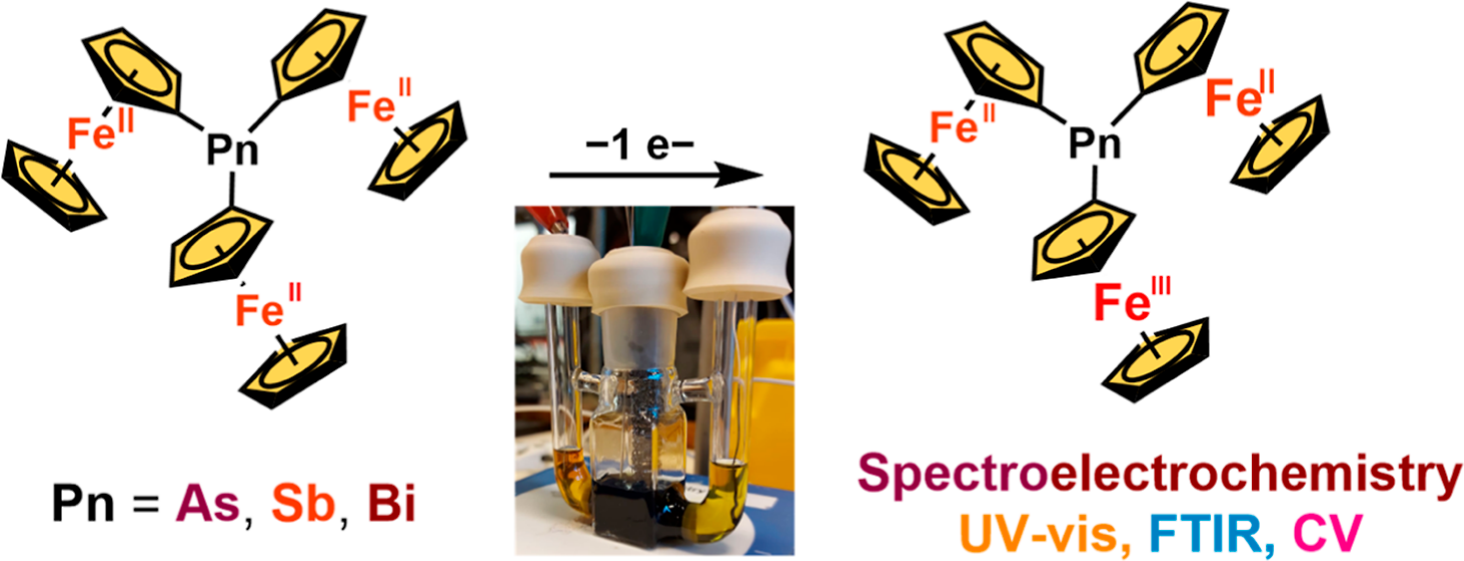

From a fundamental perspective, studies of novel mixed-valent complexes containing ferrocenyl units are motivated by the prospect of improving and extending electron transfer models and theories. Here, the series of triferrocenylpnictogens Fc_3_E was extended to the heavier analogues (E = As, Sb, and Bi), and the influence of the bridging atom was investigated with Fc_3_P as a reference. Electrochemical studies elucidate the effect of electrostatic contribution on the large redox splitting (Δ*E*_1_) exhibited by the compounds and solvent stabilization in the case of Fc_3_As. Structural characterization of the triferrocenylpnictogens combined with spectroelectrochemical studies indicates weak electronic couplings in the related cations [Fc_3_E]^+^, suggesting a through-space mechanism.

## Introduction

Since the discovery of the intensely colored pigment Prussian blue,^1^ mixed-valent (MV) compounds are subject for great fascination both for their colors and electronic properties.^2^ Such compounds find applications in the fields of material science and redox catalysis.^3−7^ MV compounds display unique physical characteristics, such as intervalence electronic transitions in the near infrared (NIR) region, which correlate with the degree of the electronic coupling between the metal centers.^8−13^ The physical parameters associated with the electronic interaction between different metals can be determined from the intervalence charge transfer (IVCT) band by employing the Marcus–Hush theory.^14−16^ Robin and Day proposed a classification for MV complexes, dividing them into three main categories.^17^ Class I complexes comprise metal centers with assignable and distinct oxidation states. Due to large separation of the metal centers or the presence of an insulating bridge between them, class I complexes do not show IVCT bands. Class II complexes exhibit broad IVCT bands due to weak interactions between the metal centers, which, however, retain their individual properties. Class III complexes show strong electronic coupling, which stems from extensive valence delocalization. Class III complexes exhibit cooperative properties, and the IVCT bands are sharp and highly intense. There are two IVCT mechanisms, which include electron transfer through a ligand (bridge) and through space. The first mechanism involves communication by orbital overlap between the metal centers, i.e., electron hopping (or “hole” hopping in cationic species) or superexchange processes mediated via the HOMO or LUMO of the bridging ligand, while the second mechanism arises when the distance between the metal centers is greater than a few angstroms and an effective metal–ligand orbital overlap is missing. In that case, the through-space interactions are exclusively electrostatic.^15,17−19^

**Scheme 1:**
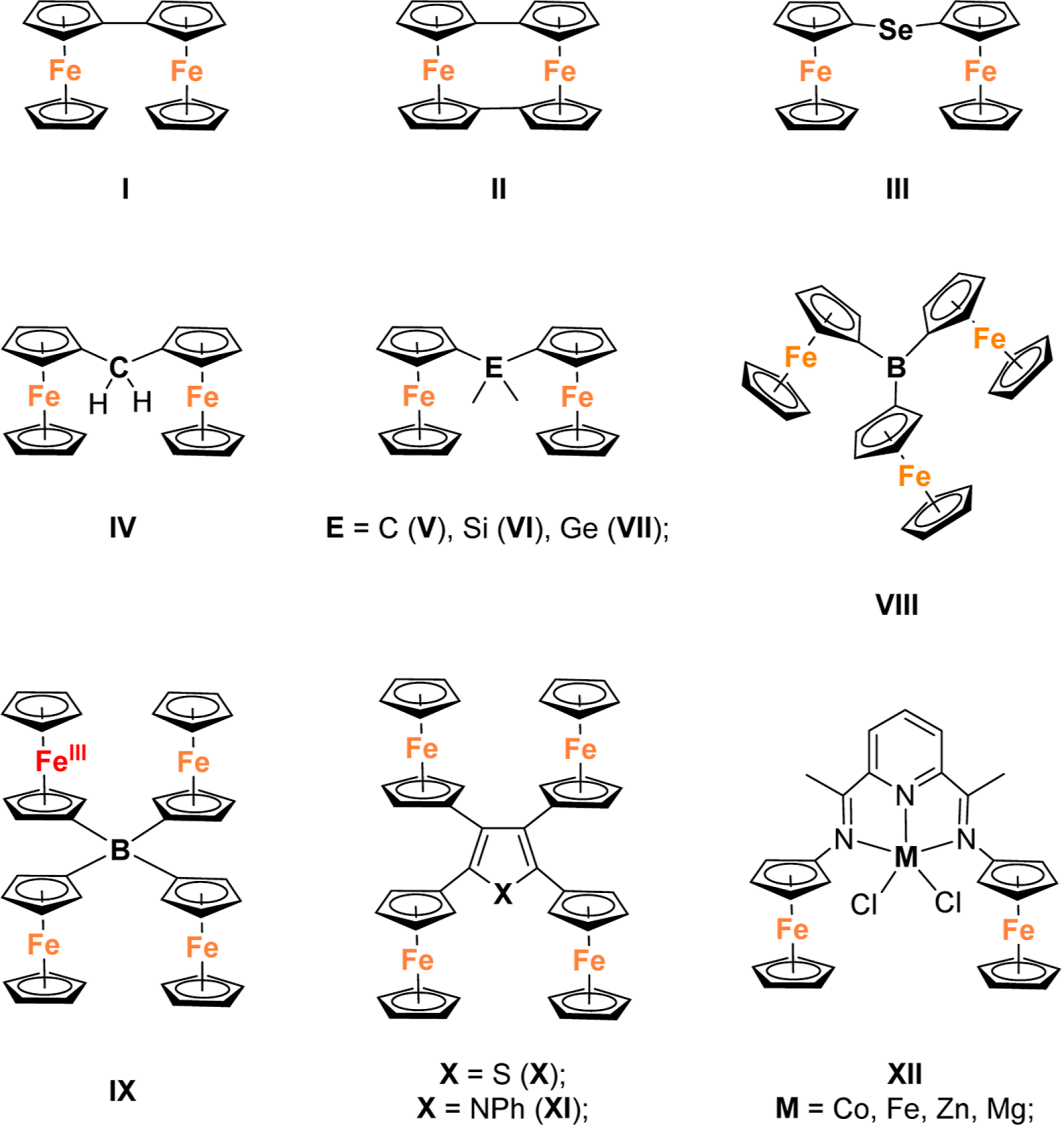
Selected Ferrocenyl-Substituted Compounds That Have Been Studied Extensively by Spectroelectrochemistry

The ferrocene(II)/ferrocenium(III) couple is one of the most widely investigated redox system owing to its chemical versatility, high thermal stability, and electrochemical reversibility.^20^ Complexes possessing multiple ferrocenyl sites, linked either via one or more atom bridges or via a delocalized system such as aryls or heterocycles, are of particular interest (Scheme 1).

The monocation derived from biferrocene (**I**) is classified as a type II complex,^21^ whereas the related cation derived from bis(fulvalene)diiron (**II**) is a classical type III complex.^22^ When an atom is bridging the two ferrocenyl sites, the electronic situation of the oxidized species is more difficult to predict. For example, in bis(ferrocenyl)selenide Fc_2_Se (**III**),^23−25^ the selenium atom acts as an insulating bridge between the two ferrocenyl moieties, similar to the methylene bridge in Fc_2_CH_2_ (**IV**),^26^ and the related monocations are class I complexes with localized Fe(II)/Fe(III) centers. However, substitution of the selenium atom in **III** with dimethyl element moieties leads to class II cations for Fc_2_EMe_2_ [E = C (**V**), Si (**VI**), and Ge (**VII**)] with predominantly through-space electronic couplings, which decline with increasing atomic number.^27^

The electrochemical properties of similar compounds have been studied extensively, with triferrocenylamine Fc_3_N (**1**),^28,29^ triferrocenylphosphine Fc_3_P (**2**),^30−32^ triferrocenylborane Fc_3_B (**VIII)**,^33,34^ and ferrocenyl-containing silanes^35,36^ and siloxanes^37^ being relevant examples. The zwitterionic neutral “borate” Fc^III^Fc^II^_3_B (**IX**) is one of few structurally characterized MV complexes for which distinct Fe(II) and Fe(III) sites were established by X-ray crystallography. Both the neutral complex **IX** and the related monocation [Fc(III)_2_Fc(II)_2_B]^+^ (**IX**^**+**^) reveal IVCT bands and belong to class II complexes.^38^ Finally, a series of multiferrocenyl compounds bridged by extensive conjugated π-systems have been studied in detail.^39−44^ The examples of tetraferrocenylthiophene (**X**)^40^ and -*N*-phenylpyrrole (**XI**)^41^ show another contrasting behavior upon oxidation. An increased value for the potential difference between the first and the second oxidation waves, Δ*E*_1_, can be indicative of a greater degree of electronic coupling.^45^ Tetraferrocenylthiophene (**X**) shows a potential difference of Δ*E*_1_ = 220 mV (CH_2_Cl_2_/[*n*-Bu_4_N][B(C_6_F_5_)_4_]), while for tetraferrocenyl-*N*-phenylpyrrole (**XI**), the potential difference is Δ*E*_1_ = 322 mV (CH_2_Cl_2_/[*n*-Bu_4_N][B(C_6_F_5_)_4_]). Although both Δ*E*_1_ are appreciably high, the latter (**XI**) shows increased electronic communication and belongs to class II complexes due to the smaller energy gap between the ferrocenyl sites and the heterocyclic bridge with increased delocalization on the C_4_N unit. In contrast, tetraferrocenylthiophene (**X**) does not exhibit significant interactions between the metal centers, hence the large Δ*E*_1_ value can be attributed to the electrostatic component. When ferrocenyl units are bridged by a “noninnocent” pyridinediimine (“pincer”) ligand (**XII**) that coordinates to various metal dichlorides MCl_2_ (M = Co, Fe, Zn, and Mg), the observed electronic coupling of the related monocations is indicative for class II complexes. The experimental and computational results unravel that the extent of mixing between the d-orbital of the ferrocenyl groups and the π orbitals of the ligand is dependent on the binding to a metal, regardless of which one it is.^6^

Since the bridging element, either by itself or in a π-conjugated system, in homometallic ferrocenyl compounds, influences the outcome of the metal–metal interactions, we decided to investigate the electrochemical behavior of the heavier triferrocenylpnictogens Fc_3_As (**3**), Fc_3_Sb (**4**), and Fc_3_Bi (**5**) in comparison with the previously studied Fc_3_P (**2**) as a reference.^30−32^ Furthermore, the nature of the lone pairs at the pnictogens might impact the interactions between the metal atoms.

## Results and Discussion

### Synthetic Aspects and Structural Characterization

**Scheme 2:**
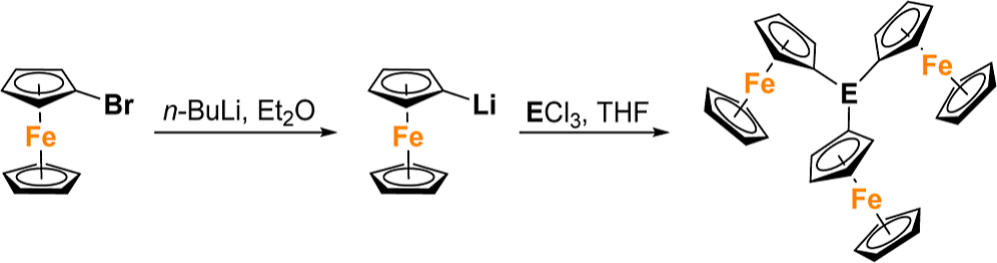
Synthesis of the Triferrocenylpnictogens Fc_3_E [E = P (**2**), As (**3**), Sb (**4**), and Bi (**5**)]

Originally, Fc_3_P (**2**) was prepared by a Friedel–Crafts reaction of ferrocene with PCl_3_.^46^ Later, **2** was obtained in higher yields by the reaction of FcLi with PCl_3_.^31^ Following this synthetic protocol (Scheme 2), we obtained the heavier congeners Fc_3_As (**3**), Fc_3_Sb (**4**), and Fc_3_Bi (**5**) in yields of 83%, 81%, and 63%, respectively. Compounds **4** and **5** were mentioned elsewhere for their chromatographic behavior, but their preparation has not been described.^47^

**Figure 1:**
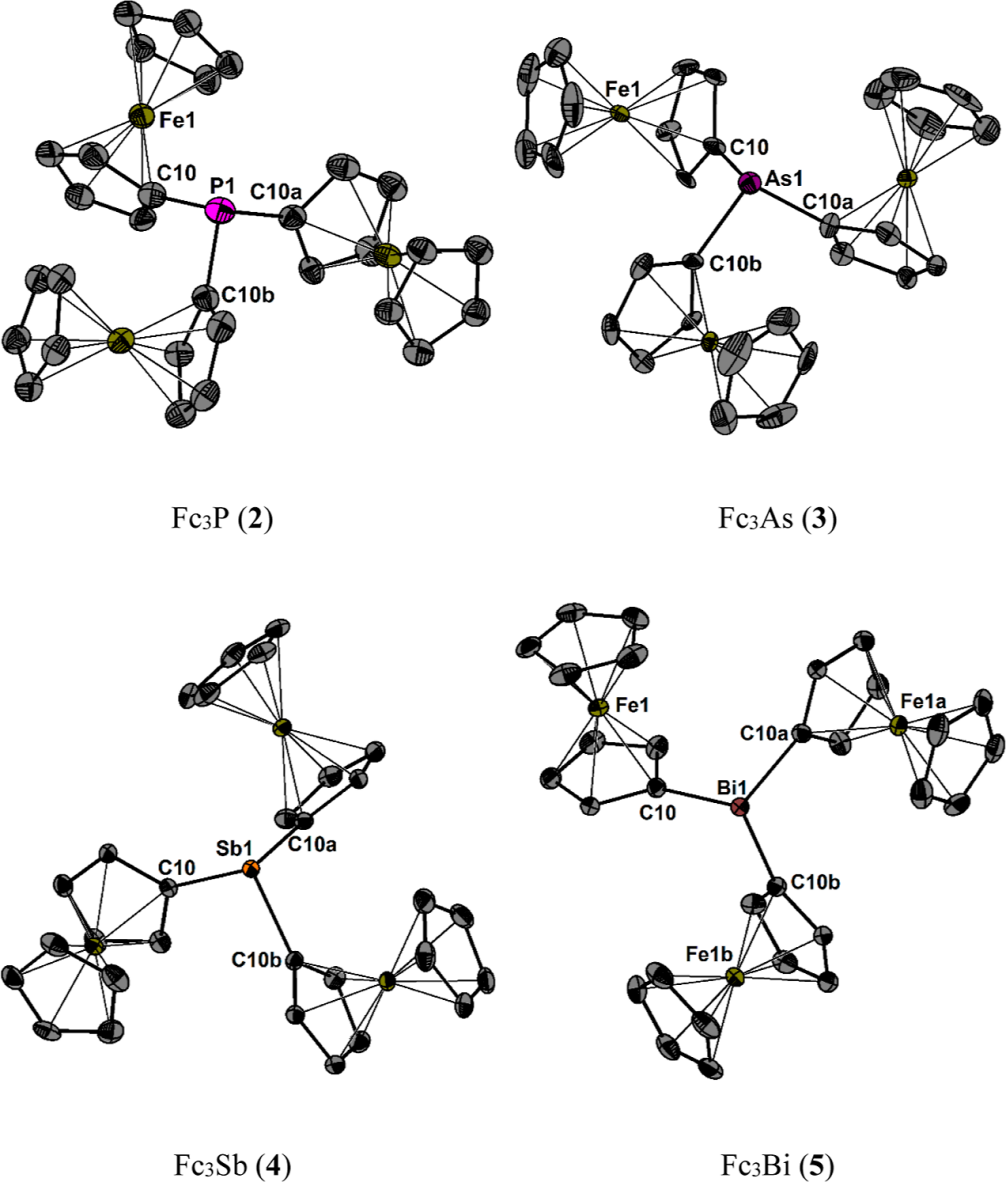
Molecular structures of Fc_3_E (E = P, As, Sb, and Bi) showing 50% probability ellipsoids and the atomic numbering scheme. Hydrogen atoms are omitted for clarity.

Although Fc_3_P (**2**) was first isolated over five decades ago, its molecular structure has not yet been not reported earlier. The molecular structure of **5** has been reported at room temperature.^48^ For the sake of comparison in the present study, the molecular structures of **2**–**5** at 100 K, as established by single-crystal X-ray diffraction, are shown in Figure 1. Selected bond parameters of Fc_3_N (**1**),^28^ Fc_3_P (**2**), Fc_3_As (**3**), Fc_3_Sb (**4**), and Fc_3_Bi (**5**) are collected in Table 1. The molecular structures of **1**–**5** comprise trigonal symmetry, and consequently, all three ferrocenyl groups are crystallographically equivalent. The spatial arrangement of the pnictogen atoms is trigonal pyramidal. Similarly as observed for Ph_3_E (E = N, P, As, Sb, and Bi), the E–C bond lengths increase when going down group 15 (from N to Bi).^49^

**Table 1:** Selected Distances and Angles for **Fc**_**3**_**E** (E = N, P, As, Sb, and Bi)^a^

	E = N (1)^28^	E = P (2)	E = As (3)	E = Sb (4)	E = Bi (5)
E–C [Å]	1.414(6)	1.806(8)	1.961(11)	2.130(2)	2.227(4)
	*1.420*	*1.817*	*1.935*	*2.128*	*2.223*
C–E–C [deg]	119.8(3)	101.7(4)	99.1(5)	94.46(8)	92.63(15)
	*115.4*	*100.59*	*97.69*	*95.08*	*93.43*
*h*_Π*-E*_ [Å]	0.06	0.81	0.94	1.13	1.22
	*0.311*	*0.834*	*0.956*	*1.114*	*1.204*
*r*_*ab*_ (Fe–Fe) [Å]		5.85	5.95	6.14	6.23
	*5.460*	*5.739*	*5.847*	*5.954*	*6.014*

a Computed gas phase-optimized values given in italics.

As the atoms become larger, the increased bond lengths of the heavier pnictogens result in reduction of steric hindrance, while the *s*/*p* hybridization defect leads to a decrease in valence orbital overlap. Consequently, lower C–E–C angles and greater pyramidalization are observed with increasing atomic number.

This also translates into greater distances when going down group 15 (*h*_Π*-E*_/Å) between the elements E (E = N, P, As, Sb, and Bi) and the plane defined by the three carbon atoms (*h*_Π*-E*_), with Fc_3_N (0.06) being almost planar.^28^ Almost all structural features are well reproduced by gas-phase geometry optimizations at the B3PW91/6-311+G(2df,p) level of theory for compounds **2**–**5**. However, larger deviations are observed for the lighter congener **1** with the gas-phase structure giving a more pyramidal arrangement as indicated by smaller C–N–C angles in conjunction with a larger *h*_Π*-E*_ distance compared to the reported experimental values (Table 1 and Figure S63).

### Electronic and Redox Properties

**Figure 2:**
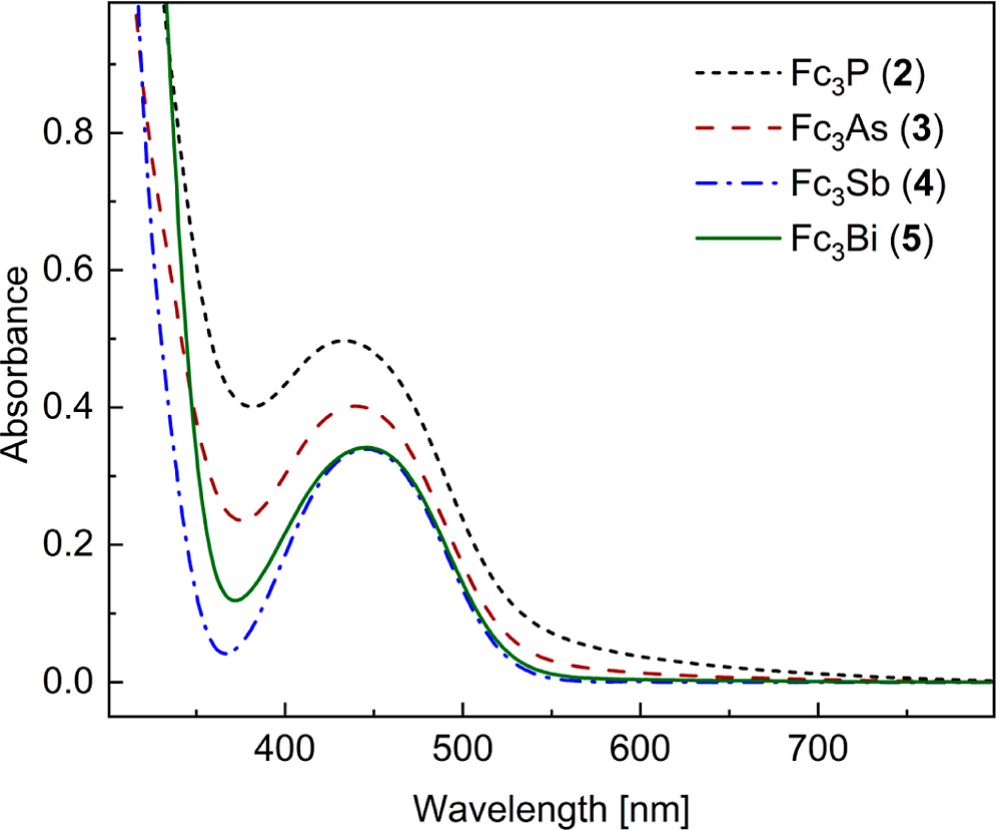
UV–vis absorption spectra of Fc_3_E [E = P (**2**), As (**3**), Sb (**4**), and Bi (**5**)] in CH_2_Cl_2_ (0.9 mM). Absorption maxima λ_max_ [nm] [ε (cm^–1^ M^–1^): 433(603) for **2**, 440(501) for **3**, 446(411) for **4**, and 446(408) for **5**].

The UV–vis absorption spectra of **2**–**5** show a broad absorption band between 350 and 550 nm, which can be attributed to ligand to metal charge transfer (LMCT), with the respective maxima slightly shifted bathochromically (“red-shifted”) when going from Fc_3_P to Fc_3_Bi (Figure 2).

**Figure 3:**
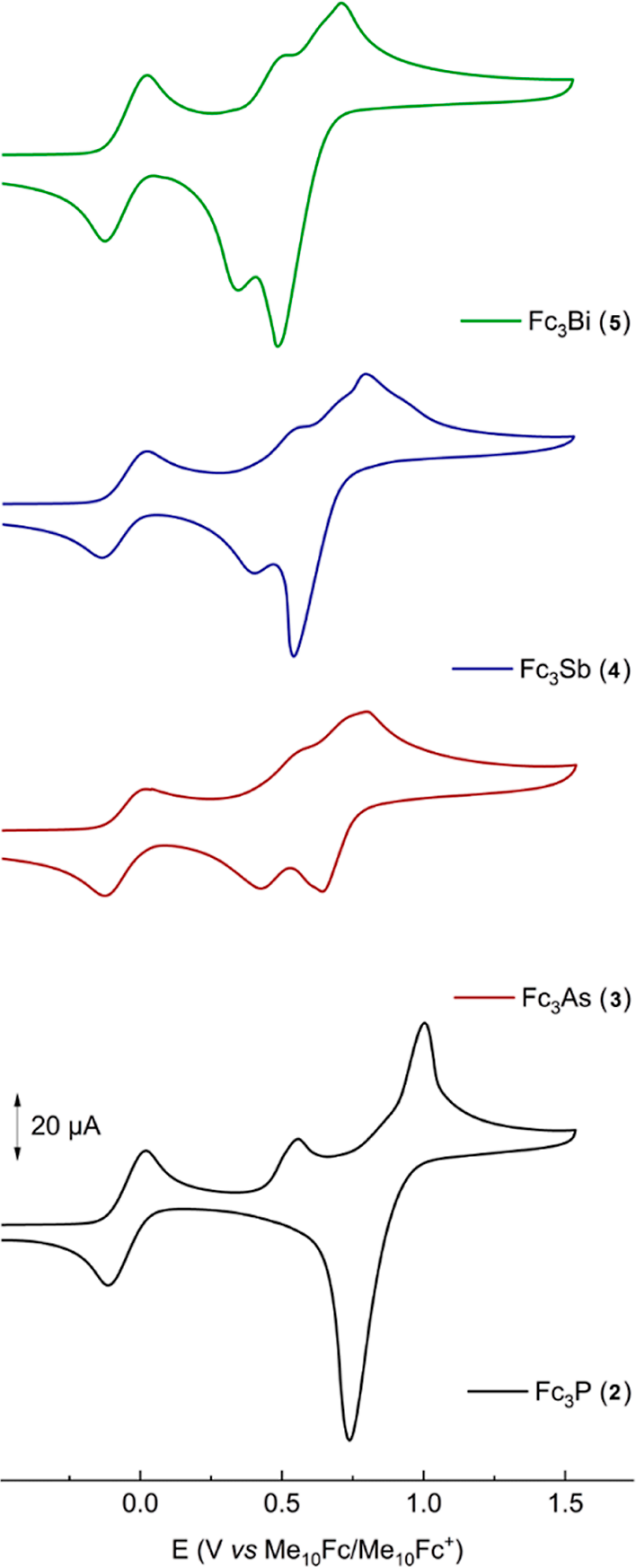
Cyclic voltammograms of **2**–**5** (1 mM) in dichloromethane at room temperature (298 K), [*n*-Bu_4_N][PF_6_] (0.1 M) as supporting electrolyte, and 100 mV/s scan rate, potential reported against Me_10_Fc/Me_10_Fc^+^ (1 mM Me_10_Fc is present in solution). Plotting convention: IUPAC (direction of the scan: toward positive potentials (to the right); initial potential: −0.1 V).

The lightest member Fc_3_N (**1**) was not further investigated in the present study, as its redox properties were previously studied by Herberhold, Britton, and co-workers.^29^ These authors reported that the cyclic voltammogram of **1**, measured in acetonitrile and NaClO_4_ supporting electrolyte, showed three reversible oxidation processes and revealed that the oxidations occur at ferrocenyl groups rather than at the nitrogen, also indicated by the first oxidation of **1**, which is shifted to negative potential (−0.31 V, E vs Fc/Fc^+^) in comparison to the corresponding primary and secondary ferrocenylamines (−0.37 and −0.36 V, respectively). The redox behavior of Fc_3_P (**2**) was previously investigated by several groups,^50,51^ but the work of Barrière, Kirss, and Geiger in particular evaluated in detail the prior results.^30^ The oxidative electrochemistry results of **2** were compared in dichloromethane, using [*n*-Bu_4_N][PF_6_] and [*n*-Bu_4_N][B(C_6_F_5_)_4_] as supporting electrolytes. The authors reported the irreversible redox chemistry of **2** in CH_2_Cl_2_/[*n*-Bu_4_N][PF_6_], due to ion pairing and poor solubility of the highly charged species (Figure 3).

However, when the cyclic voltammograms of **2** were recorded in CH_2_Cl_2_/[*n*-Bu_4_N][B(C_6_F_5_)_4_], four or more reversible oxidations were observed, with the first at −0.02 V (E vs Fc/Fc^+^, 0.60 V vs Me_10_Fc/Me_10_Fc^+^). After failing to isolate the [**2**]^**+**^ cation via bulk electrolysis, they repeated the cyclic voltammetry (CV) measurements after applying 1F/eq (*E*_appl_ = 0.2 V). Since the voltammograms showed the same three oxidations at more positive potentials, Barrière, Kirss, and Geiger concluded that the first oxidation resulted in charge delocalization and further reaction at the phosphine.^30^

We recorded cyclic voltammograms of Fc_3_E (**2**–**5**; E = P, As, Sb, and Bi) in four electrolytes (listed in Table 2) in dichloromethane at room (298 K) and low temperatures (193 K), as well as in acetonitrile with [*n*-Bu_4_N][PF_6_] (see Supporting Information for additional data) in order to study the effect of solvent and ion pairing on the electrochemical behavior. In CH_2_Cl_2_/[*n*-Bu_4_N][PF_6_], all compounds show two redox processes, with the first reversible in **3**–**5** (Figure 3). Notably, all the measurements at room temperature in CH_2_Cl_2_, except with [*n*-Bu_4_N][B(C_6_F_5_)_4_], are affected by electrode adsorption since the solubility of the highly charged cationic species generated decreases. The adsorption spikes disappear once the temperature is decreased or when acetonitrile is used instead. The first oxidation of **2** appears irreversible in the studied conditions and seems to follow an ECE “like” process. As with the results in [*n*-Bu_4_N][B(C_6_F_5_)_4_], multiple redox processes arise in MeCN/[*n*-Bu_4_N][PF_6_], which are attributed to the phosphorus radical character in the oxidized species.

**Table 2:** Redox Properties of **Fc**_**3**_**E** (E = P, As, Sb, and Bi) Recorded at 100 mV/s Scan Rate in CH_2_Cl_2_ at Room Temperature (298 K)^e^

	*E*_p,a1_ [V]	*E*_p,a2_ [V]	*E*_p,a,3_ [V] (*E*_p,a,4_ [V])	Δ*E*_1_ [mV] (*E*_p,a2_ – *E*_p,a1_)	Δ*E*_2_ [mV] (*E*_p,a3_ – *E*_p,a2_)
**2** **in: [***n***-Bu**_**4**_**N][BF**_**4**_**]**	0.53	0.63	0.81(0.98)	100	180(170)^d^
**[***n***-Bu**_**4**_**N][PF**_**6**_**]**	0.56	1.00		440	
**[***n***-Bu**_**4**_**N][SbF**_**6**_**]**	0.57	1.01		440	
**[***n***-Bu**_**4**_**N][PF**_**6**_**]**^a^^,^^b^	0.45	0.59	1.00, *br*	140	410
**[***n***-Bu**_**4**_**N][B(C**_**6**_**F**_**5**_**)**_**4**_**]**^a^	0.57	0.71	1.03(1.35)	140	320(320)^d^
**3** **in: [***n***-Bu**_**4**_**N][BF**_**4**_**]**	0.55	0.69	0.80	140	110
**[***n***-Bu**_**4**_**N][PF**_**6**_**]**	0.54	0.78		240	
**[***n***-Bu**_**4**_**N][SbF**_**6**_**]**	0.55	0.71	0.83	160	120
**[***n***-Bu**_**4**_**N][PF**_**6**_**]**^a^^,^^b^	0.37	0.53	0.68	160	150
**[***n***-Bu**_**4**_**N][B(C**_**6**_**F**_**5**_**)**_**4**_**]**^a^	0.57	0.79	1.00(1.32)	220	210(320)^d^
**4** **in: [***n***-Bu**_**4**_**N][BF**_**4**_**]**	0.53	0.76	0.92	230	160
**[***n***-Bu**_**4**_**N][PF**_**6**_**]**	0.54	0.79		250	
**[***n***-Bu**_**4**_**N][SbF**_**6**_**]**	0.53	0.69	0.78	160	90
**[***n***-Bu**_**4**_**N][PF**_**6**_**]**^a^^,^^b^	0.42	0.66		240	
**[***n***-Bu**_**4**_**N][B(C**_**6**_**F**_**5**_**)**_**4**_**]**^a^	0.51	0.78	1.07	270	290
**5** **in: [***n***-Bu**_**4**_**N][BF**_**4**_**]**	0.47	0.64		170	
**[***n***-Bu**_**4**_**N][PF**_**6**_**]**	0.50	0.71		210	
**[***n***-Bu**_**4**_**N][SbF**_**6**_**]**	0.50	0.62	0.71	120	90
**[***n***-Bu**_**4**_**N][PF**_**6**_**]**^a^^,^^b^	0.47	0.70		230	
**[***n***-Bu**_**4**_**N][B(C**_**6**_**F**_**5**_**)**_**4**_**]**^a^	0.53	0.81	1.08^c^	280	270

a 0.25 mM analytes’ concentration and 25 mM supporting electrolyte.

b MeCN/CH_2_Cl_2_ 10:1 (V/V).

c Value from measurements at 250 mV/s scan rate.

d Δ*E*_3_ = *E*_p,a4_ – *E*_p,a3_ [mV]; *br* = broad.

e Potentials are reported against the Me_10_Fc/Me_10_Fc^+^ redox couple.

**Figure 4:**
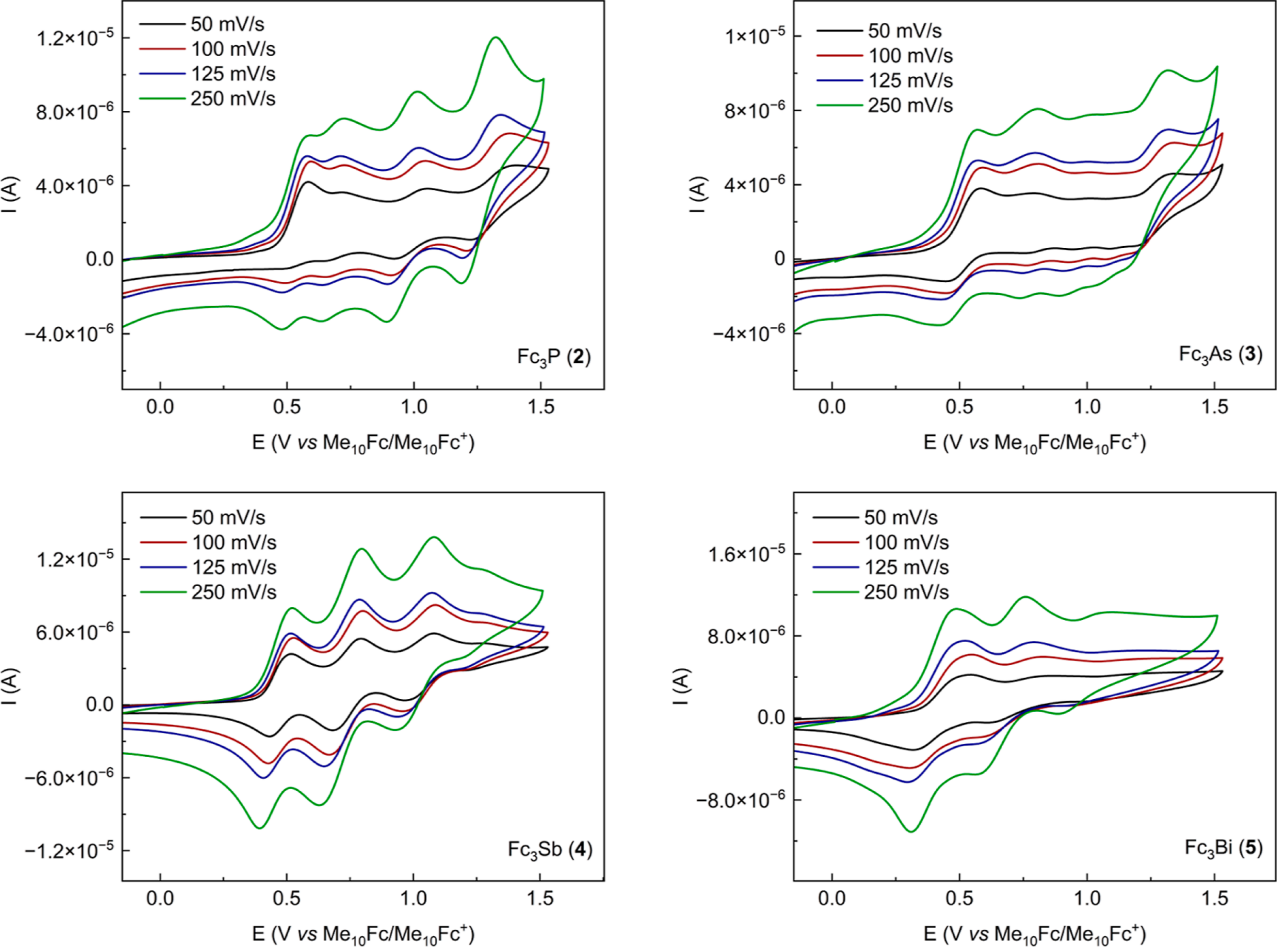
Cyclic voltammograms of **2**–**5** (0.25 mM) in dichloromethane at room temperature (298 K), [*n*-Bu_4_N][B(C_6_F_5_)_4_] (25 mM) as supporting electrolyte and different scan rates (50, 100, 125, and 250 mV/s), potential reported against Me_10_Fc/Me_10_Fc^+^. Plotting convention: IUPAC [direction of the scan: toward positive potentials (to the right); initial potential: −0.1 V].

Compounds **3**–**5** exhibit similar behavior in the studied electrolytes with minor differences. The first oxidations are reversible regardless of the chosen electrolyte, although all three compounds behave best in [*n*-Bu_4_N][SbF_6_]. They show three clear, quasi-reversible oxidation events and smaller Δ*E*_1_ values when compared to other supporting electrolytes (Table 2). On the other hand, peak-to-peak separation in [*n*-Bu_4_N][B(C_6_F_5_)_4_] appears greater, and the oxidations occur at more positive potentials as an effect of low ion pairing capability and therefore low ability to stabilize the monocations (Table 2 and Figure 4).^52^

The trend appears to be comparable for compounds **3**–**5**, with smaller Δ*E* values (both Δ*E*_1_ and Δ*E*_2_) for the compact anions, SbF_6_^–^ < BF_4_^–^ < PF_6_^–^ < [B(C_6_F_5_)_4_]^−^. This effect demonstrates the poorer donor properties of the [B(C_6_F_5_)_4_]^−^ anion and the significant contribution of the electrostatic interactions to the large Δ*E* values that can be observed.

Besides the influence of the electrolyte anion, the solvent donor properties have a significant influence on the stability of the **3**^**+**^ monocation, where the Δ*E*_1_ (240 mV) in *n*-Bu_4_N][PF_6_]/CH_2_Cl_2_ is larger than Δ*E*_1_ (160 mV) in MeCN. For compounds **4** and **5**, the effect of the solvent is minor, which indicates that the stabilization through ion pairing is more important than through solvation.

The thermodynamic stability of the MV compounds, expressed by the comproportionation constant *K*_c_, was determined for **3**, **4**, and **5** in [*n*-Bu_4_N][PF_6_] and [*n*-Bu_4_N][B(C_6_F_5_)_4_] in CH_2_Cl_2_ (Table 3). The values of *K*_c_ increase significantly when the MV species bear electrostatic interactions or charge delocalization, and a value above four (the statistical value) indicates an increase in the stability of the MV state.^13^ The values in [*n*-Bu_4_N][PF_6_] (*K*_c1_) do not follow a particular trend, as opposed to *K*_c2_ (in [*n*-Bu_4_N][B(C_6_F_5_)_4_]), where the values increase significantly from **3** to **5**. The big differences between the two electrolytes are also a consequence of electrostatic contribution.

**Table 3:** Intervalence Interaction Strength Data of the Cations [Fc_3_E]^+^ (E = As, Sb, and Bi) Calculated from the IVCT Band Obtained after Electrochemical One Electron Oxidation of the Bulk Analyte in CH_2_Cl_2_ and [*n*-Bu_4_N][PF_6_] as Supporting Electrolyte at Room Temperature (298 K)

	*K*_c1_^a^	*K*_c2_^a^	**α**	Δ**ν**_**max**_**[cm**^**–1**^**]**	ε_max_ L·mol^–1^·cm^–1^	Δ**ν**_1/2_**[cm**^**–1**^**]**	Δ**ν**_1/2_**(Hush) [cm**^**–1**^**]**	***H***_***ab***_**[cm**^**–1**^**]**
**[3]**^+^	11,394	5231	0.039	5260	112	11916	3405	205
**[4]**^+^	16,815	36622	0.019	8044	82	6382	4243	154
**[5]**^+^	3545	54046	0.019	8236	100	5506	4306	158

a Comproportionation constant, *K*_c_ = exp {Δ*E*_1_*F*/*RT*}, where *F*/*RT* takes the value 38.92 V^–1^ at 298 K.^13^ The Δ*E*_1_ value (V) is extracted from the CV measurements in CH_2_Cl_2_ and [*n*-Bu_4_N][PF_6_] as SE for *K*_c1_ and [*n*-Bu_4_N][B(C_6_F_5_)_4_] as SE for *K*_c2_ at room temperature (298 K).

### Spectroelectrochemistry

Since Δ*E* and *K*_c_ are not a quantitative representation of the electronic coupling,^45,53^ we proceeded to further investigate the internuclear interactions using spectroelectrochemical techniques. The measurements were performed in CH_2_Cl_2_ with [*n*-Bu_4_N][PF_6_] as the supporting electrolyte (the experiments are described below). The neutral compounds Fc_3_E **2**–**5** do not show absorption bands in the NIR region. Upon one electron oxidation, MV Fe(II)/Fe(III) monocations were expected for **3**–**5**. The first oxidation of **2** was performed at high voltage (*E*_apl_ = 1.0 V) and did not result in absorbance in the NIR region, most likely because of the charge delocalization and and/or potential phosphine oxidation. However, an increase in absorbance in the 550–850 nm region (see Supporting Information), that can be attributed to LMCT, was observed. These results are in accordance with those reported previously in a NIR spectroscopic study of multiferrocenyl compounds.^32^ The absence of the IVCT band in the Fc_3_P^+^ [**2**]^**+**^ monocation and its presence in the oxidized species Fc_3_PO^+^ (reported in CH_2_Cl_2_/[Et_4_N][ClO_4_]) were previously attributed to the difference between the two systems when taking into account the dp−π orbital overlap of phosphorus and the cyclopentadienyl rings.

**Figure 5:**
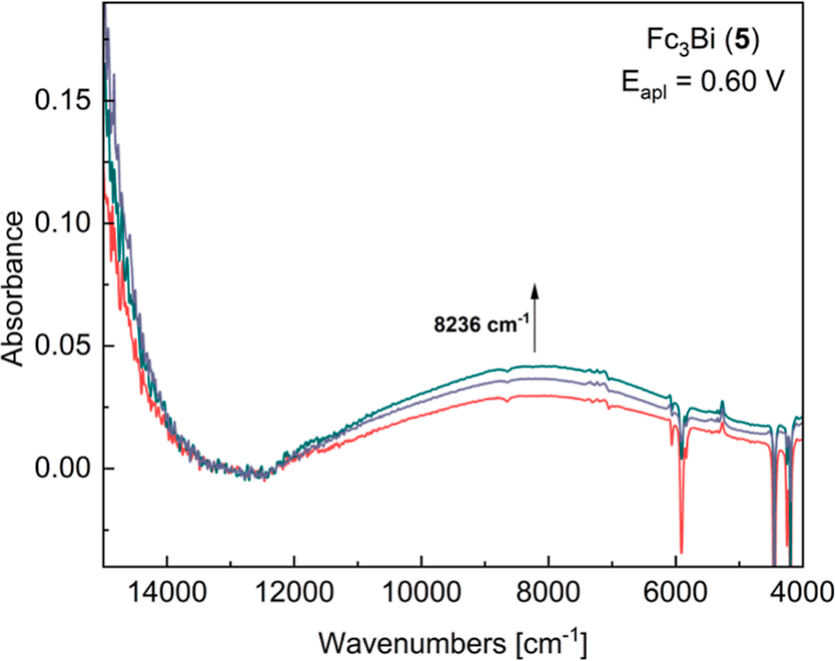
NIR absorption spectra of Fc_3_Bi (**5**) at different times during bulk oxidation (600, 1200, 1800, and 2400 s; applied voltage of 0.60 V for the first oxidation); negative bands due to CH_2_Cl_2_ overtones.

After electrochemical oxidation, the freshly obtained cations [**3**]^**+**^, [**4**]^**+**^, and [**5**]^**+**^ showed a broad NIR band each, characteristic for the electronic intermetallic interactions (Figure 5). These cations were characterized using a three-state model. The physical parameters of the IVCT excitations were determined from the Gaussian fit of the experimental spectra by applying a modification of the Hush formula.^15,16,54^ All three compounds present weak intervalence interactions with low extinction coefficients, which is consistent with weak internuclear coupling (*H*_*ab*_) values (Table 3). Although the degree of the electronic communication is relatively small (154–205 cm^–1^), the values are higher than the one of [Fc_3_CH]^+^ (24 cm^–1^)^32^ and comparable to the [Fc_2_EMe_2_]^+^ [E = C (**V**), Si (**VI**), and Ge(**VII**)] complexes (*H*_*ab*_ = 153–337 cm^–1^).^27^ Notably, [**4**]^**+**^ and [**5**]^**+**^ exhibit similar electronic couplings and reorganization energies (Δν_max_ = λ), while for [**3**]^**+**^, the energy is higher and the IVCT band is wider. The theoretical peak width at half height [Δν_1/2_ (Hush)] values and their correlation with the experimental ones are also affected: compounds [**4**]^**+**^ and [**5**]^**+**^ present smaller differences between the Δν_1/2_ values; the experimental value for [**3**]^**+**^ is roughly three times larger than the theoretical one.

When the degree of the electronic coupling in the order of [**3**^**+**^] > [**5**^**+**^] ≥ [**4**^**+**^] (as *H*_*ab*_ is an estimated, not absolute value) and geometry similarities are taken into consideration, a through-space operative mechanism would be plausible. While the through-bond interaction could bring a smaller contribution to the CT mechanism, it cannot be entirely excluded. Noteworthy, the IVCT bands disappear in all the cases when the second oxidation is achieved.

Besides the presence of the IVCT band in [**3**]^**+**^ (ε_628_ = 680 L·mol^–1^·cm^–1^), [**4**]^**+**^ (ε_625_ = 500 L·mol^–1^·cm^–1^), and [**5**]^**+**^ (ε_626_ = 580 L·mol^–1^·cm^–1^), additional LMCT bands could be observed in the 626–628 nm region, with a small shoulder at *ca*. 573 nm (see Supporting Information) characteristic to the Fe(III) in ferrocenyl-containing compounds. The absorbance of the initial neutral compounds increases as well and shows a small bathochromic (“red”) shift.

### (TD)-DFT Calculations

Time-dependent density functional theory (TD-DFT) calculations were performed for compounds **1**–**5** and the predicted absorption maxima are consistent with the experimental observation (Figure 2 and Table S3). Furthermore, the computed transitions have low oscillator strengths, which correlate with the experimentally observed low molar absorptivities. The respective excitations are composed of multiple orbital transitions with similar contributions, which makes a visualization impractical even with the natural transition orbital approach. Generally, the involved orbitals show little contribution from the pnictogens and indicate that the absorption stems mostly from the ferrocenyl units.

**Figure 6:**
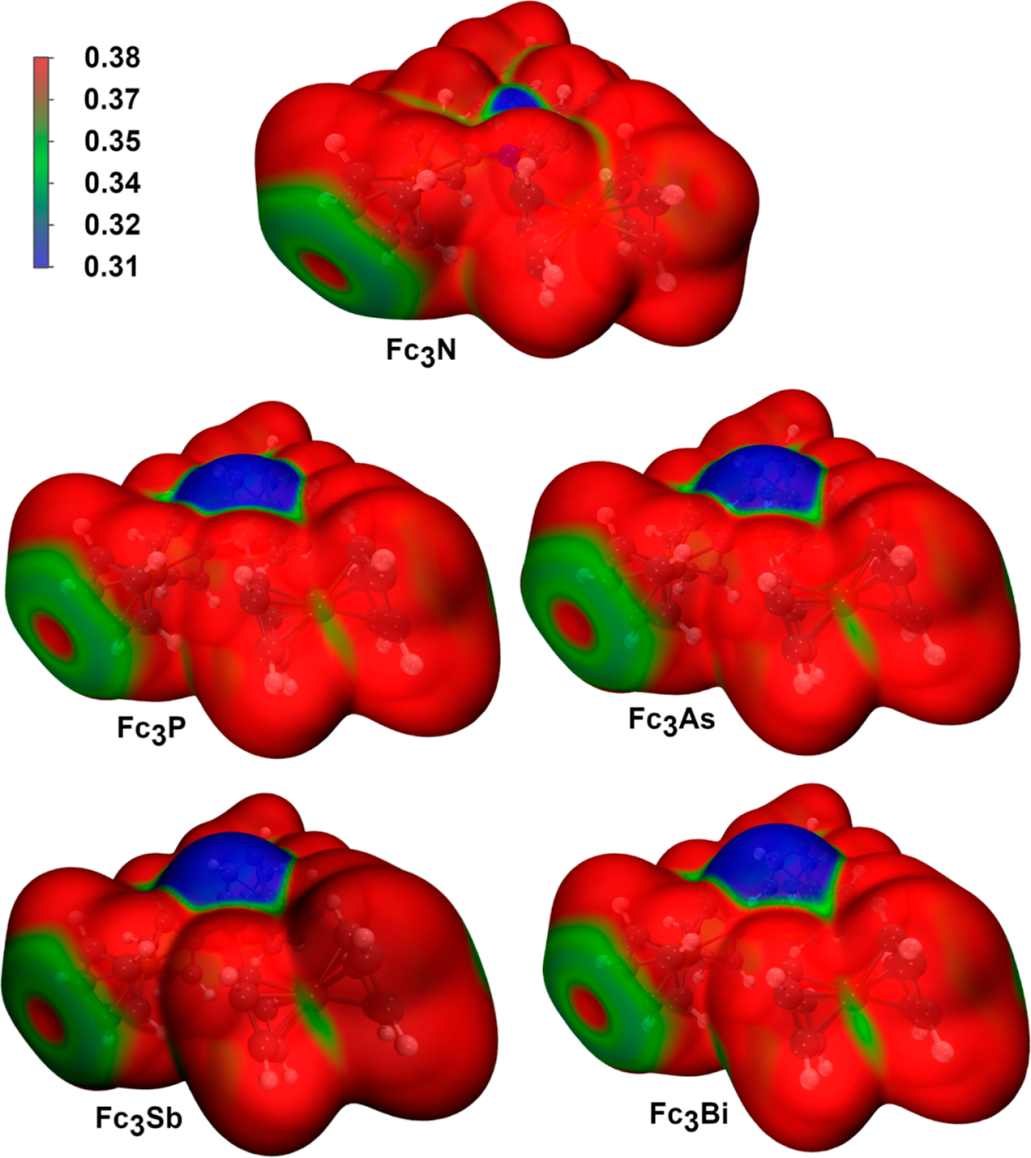
ALIE on 0.0005 a. u. electron density iso-surface of **1**–**5**.

The average local ionization energy (ALIE) is the energy required to remove an electron at a given locus of a molecule.^55^ The lowest value correlates with the location where the electrons are least tightly held. Analysis of the ALIE of **1**–**5** revealed that the most loosely bound electrons reside at the pnictogens, suggesting that initial oxidation most likely occurs at the pnictogens (Figure 6). Due to the increasing pyramidal arrangement, this effect is more pronounced for the heavier pnictogens.

Finally, we attempted the geometry optimization of the cations [**1**]^+^–[**5**]^+^ in the gas-phase geometry at the B3PW91/6-311+G(2df,p) level of theory; however, only for [**1**]^+^ and [**3**]^+^, convergence was reached. Compared to that of **1**, the spatial arrangement of [**1**]^+^ is nearly trigonal planar, whereas the degree of pyramidalization of [**3**]^+^ is nearly the same as in **3**.

**Figure 7:**
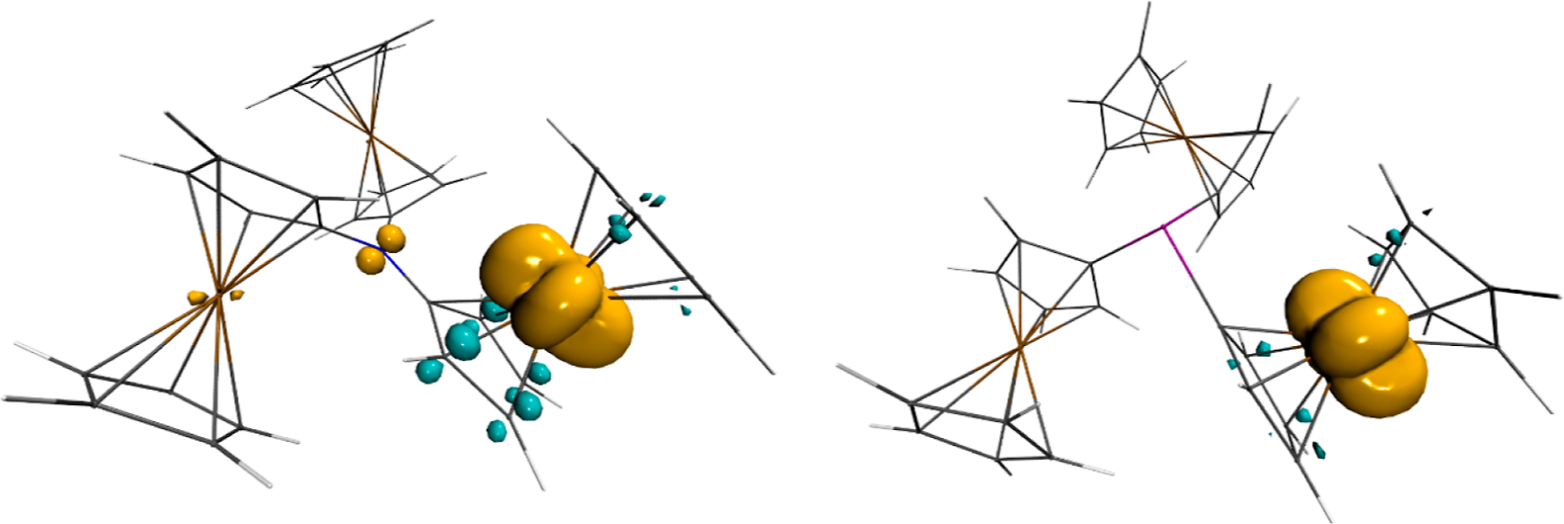
Spin densities of [**1**]^+^ (left) and [**3**]^+^ (right). Representation at +0.005/–0.005 e bohr^–3^ (orange/cyan).

For both compounds, oxidation induced an asymmetry in the E–C bond lengths. In [**1**]^+^, one N–C bond length (1.345 Å) is significantly shorter than the other two (1.417 and 1.425 Å). In [**3**]^+^, one As–C bond length (1.969 Å) is significantly longer than the other two values (1.922 and 1.926 Å). These observations suggest discrete Fe(II) and Fe(III) sites rather than pnictogen-centered radical cations, which was indeed confirmed by the calculation of the spin densities (Figure 7).

Only for [**1**]^+^, a small fraction of the spin density is situated at the nitrogen atom, whereas the vast majority of the spin density of [**1**]^+^ and [**3**]^+^ is located at the iron atom of one ferrocenyl moiety.

## Conclusions

Structural, spectroelectrochemical, and computational evidence argue that the degree of through-space electronic interaction of Fc moieties in Fc_3_E (E = As, Sb, and Bi) upon one electron oxidation is weak but not negligible in pnictogen-linked ferrocenyls. Extended electrochemical studies show the importance of cation stabilization through ion pairing with the electrolyte anion in Fc_3_Sb (**4**) and Fc_3_Bi (**5**), while in Fc_3_As (**3**) the solvent donor properties play an additional role. In a similar manner as Fc_2_EMe_2_ compounds of group 14 elements,^27^ the present study allows systematic comparison of *H*_*ab*_ and α values. While in Fc_3_E (E = P, As, Sb, and Bi) the E–C bonds increase when going down the group and the spatial proximity between the Fc sites increases as well (as there is more space to accommodate three ferrocenyl units), the geometries of the compounds are similar, and a through-space mechanism is most probable. While through-bond coupling might not play an important role in the CT mechanism, it cannot be excluded. The presence of IVCT bands in [Fc_3_CH]^+^ and [Fc_4_B]^+^ could indeed be explained by a through-space mechanism as a result of the more crowded environment around the central atom; the difference between the Fc_3_P (**2**) and Fc_3_PO does not lie in geometrical dissimilarities.^32,33^ As an IVCT band upon oxidation was observed only in the latter compound, the result could be explained by the charge delocalization to the phosphorus atom; thus, the measured compound was a byproduct. This observation highlights the importance of a thorough electrochemical and spectroscopic analysis. Lastly, the observed weak interactions in the heavier derivatives ask for structural changes that can increase orbital energy overlap and, therefore, electronic coupling in similar complexes.

## Experimental Section

Unless otherwise stated, all reactions and purifications were performed under an argon atmosphere using anhydrous solvents. The reagents used in this work including ferrocene, AsCl_3_, SbCl_3_, BiCl_3_, and *n*-BuLi were obtained commercially and used as received. FcBr,^55^ Fc_3_P,^31^ and [*n*-Bu_4_N][B(C_6_F_5_)_4_]^52^ were prepared following the procedures described in the literature. Anhydrous dichloromethane, tetrahydrofuran, hexane, and acetonitrile were collected from an SPS800 mBraun solvent purification system and stored over 3 Å molecular sieves. Diethyl ether was dried by heating at reflux over Na/benzophenone under an argon atmosphere. Deuterated solvents were degassed and dried over 3 Å molecular sieves under argon.

Unless noted otherwise, NMR spectra were recorded at room temperature on a Bruker AVANCE 600 MHz spectrometer. ^1^H and ^13^C{^1^H} spectra are reported on the δ scale (ppm) and are referenced against those of SiMe_4_. ^1^H and ^13^C{^1^H} chemical shifts are reported to the residual peak of the solvent (CDHCl_2_ 5.32 ppm for CD_2_Cl_2_) in the ^1^H NMR spectra and to the peak of the deuterated solvent (CD_2_Cl_2_ 53.84 ppm) in the ^13^C{^1^H} NMR spectra.^56^ The assignment of the ^1^H and ^13^C{^1^H} signals was made in accordance with the COSY, HMBC, and HSQC spectra.

The ESI HRMS spectra were measured on a Bruker Impact II spectrometer. Dichloromethane, acetonitrile, or dichloromethane/acetonitrile solutions (*c* = 1 × 10^–5^ mol·L^–1^) were injected directly into the spectrometer at a flow rate of 3 μL·min^–1^. Nitrogen was used both as a drying gas and for nebulization with flow rates of approximately 5 L·min^–1^ and a pressure of 5 psi. Pressure in the mass analyzer region was usually about 1 × 10^–5^ mbar. Spectra were collected for 1 min and averaged. The nozzle-skimmer voltage was adjusted individually for each measurement.

UV–vis absorption spectra were recorded on a VWR UV-1600PC spectrophotometer and analyzed using Spectragryph software.^57^ UV–vis absorption spectra for the spectroelectrochemical experiments were recorded on a SPECORD 210 PLUS Double Beam UV–vis spectrophotometer, Analytik Jena (±0.1 nm accuracy). NIR spectra were recorded on an FTIR-4600 (0.7 cm resolution) Jasco spectrometer. Spectroelectrochemical measurements were carried out on a Model 760c electrochemical workstation (CHInstruments).

For all electrochemical experiments, spectroscopic grade solvents were employed. All the supporting electrolytes were used as received, except for [*n*-Bu_4_N][B(C_6_F_5_)_4_] (vide supra). CV studies were performed in a V-tube electrochemical cell. The CV data was recorded at room temperature (ca. 25 °C) and at −80 °C using a Autolab PGSTAT 101 (Metrohm) electrochemical workstation. A three-electrode configuration was used with a glassy carbon (CHInstruments CHI104) acting as a working electrode (WE), a Pt wire acting as a counter electrode (CE), and an Ag/AgCl pseudo reference electrode (pRE). Before every measurement, the CE was polished to a mirror-like appearance with diamond paste (1 μm), carefully rinsed with deionized water, sonicated, and then rinsed with HPLC-grade acetone. The decamethylferrocene/decamethylferrocenium redox couple (Me_10_Fc/Me_10_Fc^+^, *c* = 1 mM) was used as internal standard for the measurements in CH_2_Cl_2_, except for the ones with [*n*-Bu_4_N][B(C_6_F_5_)_4_] (25 mM) as supporting electrolyte. In the latter arrangement, as well as in the measurements in acetonitrile, the cyclic voltammograms were referenced externally with the Me_10_Fc/Me_10_Fc^+^ redox couple (*c* = 0.25 mM). The analytes were measured as 1 mM solutions in CH_2_Cl_2_, and [*n*-Bu_4_N][BF_4_], [*n*-Bu_4_N][PF_6_], and [*n*-Bu_4_N][SbF_6_] (0.1 M) were used as supporting electrolytes. The measurements in acetonitrile/dichloromethane (MeCN/CH_2_Cl_2_ 10:1 V/V) employed lower concentrations of analytes (0.25 mM) and [*n*-Bu_4_N][PF_6_] (25 mM). The solutions were degassed with argon prior to measurement. The starting potential for all the CV measurements is 0.1 V, while the direction of the initial scan is oxidative (goes to positive potentials). All the measurements were performed at the following scan rates: 50, 100, 125, and 250 mV/s. The IUPAC plotting convention was used for all of the CV illustrations.

Bulk electrolysis experiments were performed under constant potential in a H-cell, divided by porous glass frit, granulate 3 (medium), while slowly purging argon through (using septa to keep oxygen and water out). Periodically, volumes of ∼0.3 mL were taken out, transferred to 0.1 cm path length quartz cuvettes, and UV–vis spectra were recorded. The NIR measurements were performed in 1 cm path length quartz cuvettes. Analytes’ concentrations of 5 mM in anhydrous CH_2_Cl_2_ containing 0.1 M [*n*-Bu_4_N][PF_6_] were prepared and degassed. A carbon mesh WE, platinum wire CE, and Ag/AgCl pRE were employed. The bulk electrolysis voltages were as follows: 1.0 V for Fc_3_P (**2**), 0.75 V for Fc_3_As (**3**), 0.70 V for Fc_3_Sb (**4**), and 0.60 V for Fc_3_Bi (**5**). The redox processes (oxidation, then reduction, multiple times) were also followed in the same setup, this time using the AvaSpec-ULS2048CL-EVO-RS-UA to evaluate the changes in the visible region.

### Synthesis and Characterization of Fc_3_As (**3**)

A solution of bromoferrocene (1.00 g, 3.77 mmol) in Et_2_O (15 mL) was cooled to −80 °C, and *n*-BuLi (1.60 mL, 4.00 mmol) was added to it dropwise. The reaction mixture was stirred at low temperature for ca. 40 min and then warmed to 0 °C. After 30 min at 0 °C, the orange suspension was cooled to −60 °C, and *n*-hexane (10 mL) was added. The now-yellow suspension was stirred at low temperature for 10 min and filtered to give FcLi as a bright orange solid. At the same temperature, FcLi was washed with 5 more mL of *n*-hexane and then dissolved in THF (10 mL). The suspension was cooled to −80 °C, and AsCl_3_ (85.0 μL, 1.03 mmol) was added slowly. After 1.5 h of stirring at −80 °C, the cooling bath was removed. The reaction mixture was stirred for 45 min at room temperature, and the solvent was removed *in vacuo* to dryness. Under ambient conditions, 10 mL CH_2_Cl_2_ was added to the dark yellow solid, and then the suspension was filtered over Celite to remove the inorganic salts. The solvent was removed under reduced pressure, and the resulting solid was washed with 3 × 5 mL of *n*-hexane and with additional 5 mL of MeCN. The light-yellow solid was dried under reduced pressure, resulting in 83% yield (0.54 g, 0.86 mmol). Single crystals suitable for X-ray diffraction were obtained by liquid-to-liquid diffusion (layering a solution of 3 in CH_2_Cl_2_ with *n*-hexane). **Mp** = 163–164 °C (decomp.). ^**1**^**H NMR (600 MHz, CD**_**2**_**Cl**_**2**_**)**: δ = 4.28 (m, 6H, As-Cp), 4.12 (m, 6H, As-Cp), 4.06 (s, 15H, Cp). ^**13**^**C{**^**1**^**H} NMR (151 MHz, CD**_**2**_**Cl**_**2**_**)**: δ = 79.2 (s, *i*-C), 73.1 (s, As-Cp), 70.2 (s, As-Cp), and 69.2 (s, Cp). **HRMS ESI (***m***/***z***)**: [M]^+^ calcd for C_30_H_27_Fe_3_As, 629.9371; found, 629.9358.

### Synthesis and Characterization of Fc_3_Sb (**4**)

To a precooled (−80 °C) suspension of FcLi (1.31 g, 6.60 mmol) in THF (30 mL) was added a solution of SbCl_3_ (0.46 g, 2.00 mmol) in THF (10 mL). The reaction mixture was allowed to warm up to room temperature over the course of 2 h. All volatiles were evaporated to dryness, and the next steps of purification were performed under ambient conditions. CH_2_Cl_2_ (35 mL) was added, and the suspension was filtered over Celite. The brown solid was washed with additional CH_2_Cl_2_ (5 mL) and dried under reduced pressure. The resulting solid was washed with MeCN (5 × 4 mL) and dried under vacuum. The product **4** was obtained as a yellow solid (1.09 g, 1.61 mmol, 81%). **Mp** = 212–213 °C (decomp.). ^**1**^**H NMR (600 MHz, CD**_**2**_**Cl**_**2**_**):** δ = 4.33 (m, 6H, Sb-Cp), 4.15 (t, ^3^*J*(^1^H–^1^H) = 2 Hz, 6H, Sb-Cp), 4.05 (s, 15H, Cp). ^**13**^**C{**^**1**^**H} NMR (151 MHz, CD**_**2**_**Cl**_**2**_**):** δ = 75.1 (s, Sb-Cp), 71.2 (s, Sb-Cp), 68.9 (s, Cp). **HRMS ESI (***m***/***z***):** [M]^+^ calcd for C_30_H_27_Fe_3_Sb, 675.9201; found, 675.9197.

### Synthesis and Characterization of Fc_3_Bi (**5**)

To a precooled (−80 °C) suspension of FcLi (1.34 g, 6.60 mmol) in THF (20 mL), BiCl_3_ (0.63 g, 2.00 mmol) was added as a solid. The reaction mixture was allowed to warm up to room temperature over the course of 3 h. All volatiles were evaporated to dryness, and the next steps of purification performed under ambient conditions. CH_2_Cl_2_ (40 mL) was added, and the suspension was filtered over Celite. The brown solid was washed with additional CH_2_Cl_2_ (2 × 10 mL) and dried under reduced pressure. The resulting solid was washed with an acetone/CH_2_Cl_2_ mixture (9:1, volume ratio) (2 × 5 mL) and MeCN (3 mL) and dried under vacuum. The product **5** was obtained as an orange solid (0.97 g, 1.27 mmol, 63%). **Mp** = 193–194 °C (decomp.). ^**1**^**H NMR (600 MHz, CD**_**2**_**Cl**_**2**_**):** δ = 4.30 (s, br, 6H, Bi-Cp), 4.23 (s, br, 6H, Bi-Cp), 4.08 (s, 15H, Cp). ^**13**^**C{**^**1**^**H} NMR (151 MHz, CD**_**2**_**Cl**_**2**_**):** δ = 77.0 (s, Bi-Cp), 71.8 (s, Bi-Cp), 68.8 (s, Cp). **HRMS ESI (***m***/***z***):** [M]^+^ calcd for C_30_H_27_Fe_3_Bi, 763.9961; found, 763.9952.

### X-ray Crystallography

Single crystals of **2**–**5**, suitable for X-ray structure determination, were obtained from liquid-to-liquid diffusion of a solution of the analyte in CH_2_Cl_2_ or CHCl_3_ and *n*-hexane. Intensity data were collected on a Bruker Venture D8 diffractometer at 100 K with graphite-monochromated Cu-Kα (1.5418 Å) for **2**·CH_2_Cl_2_ and Mo-Kα (0.7107 Å) radiation for **3**·CHCl_3_, **4**, and **5**, respectively. All structures were solved by direct methods and refined based on F^2^ by use of the SHELX program package as implemented in OLEX2 version 1.5.^58^ All crystals were affected by merohedral twinning and were refined applying the same twin law (−1, 0, 0, −1, 0, 0, 0, 0, 1). All non-hydrogen atoms were refined using anisotropic displacement parameters. Hydrogen atoms attached to carbon atoms were included in geometrically calculated positions by using a riding model. Crystal and refinement data are collected in Tables S1 and S2. Figures were created using DIAMOND.^59^ Crystallographic data for the structural analyses have been deposited with the Cambridge Crystallographic Data Centre, CCDC numbers 2352723–2352726. Copies of this information may be obtained free of charge from the Director, CCDC, 12 Union Road, Cambridge CB2 1EZ, UK (Fax: +44-1223-336,033); e-mail: deposit@ccdc.cam.ac.uk or http://www.ccdc.cam.ac.uk.

### Computational Methodology

Geometry optimizations of Fc_3_E (E = N, P, As, Sb, and Bi) as well as Fc_3_N^+^ were performed using DFT with the B3PW91^60^ functional and the 6-311+G(2df,p)^61^ basis set (for H, C, N, P, As, and Fe) as well as the cc-pVTZ-PP basis set (for Sb and Bi).^62^ The basis sets as well as the effective core potentials for the Sb and Bi atoms were obtained from the Basis Set Exchange Library.^63^ Subsequent frequency analyses confirmed the obtained structures for Fc_3_E (E = N, P, Sb, or Bi) to be local minima on the potential energy surfaces. Despite all efforts, a small negative frequency of −6.1637 cm^–1^ remained in the optimized geometry of Fc_3_As. Dispersion effects were modeled using Grimme’s GD3BJ parameters.^64^ The vertical excitation energies of the 20 lowest singlet states were predicted by TD-DFT computations using the level of theory stated above. All geometry optimizations were performed with the Gaussian16 software.^65^

The ALIE^66^ analysis was performed with Multiwfn_3.8.^67^ Geometry superposition figures, ALIE iso-surfaces, and spin densities are displayed with VMD.^68^

## Data Availability

The data underlying this study are available in the published article and the accompanying Supporting Information.

## Supporting Information Available

Characterization data (^1^H and ^13^C NMR and UV–vis), additional CVs and spectroelectrochemistry data (UV–vis and FT-NIR), crystallographic structures and refinement data, and details of the TD-DFT calculations (PDF)
Combined DFT-optimized coordinates (XYZ)

## Supplementary Material

gg4c00034_si_001.pdf

gg4c00034_si_002.xyz

## Dedication

Dedicated to Professor Konrad Seppelt on the occasion of his 80th birthday.
