# Supplementary material for: Electronic Coupling in Triferrocenylpnictogens
Source: ACS Org Inorg Au. 2024 Aug 17;4(5):545–56. doi: 10.1021/acsorginorgau.4c00034 (PMC11450725; doi:10.1021/acsorginorgau.4c00034)
Supplement: Supplementary file 1 — gg4c00034_si_001.pdf [file gg4c00034_si_001.pdf]

Supporting Information

Electronic Coupling in Triferrocenylpnictogens

Corina Stoian,[a] Fawaz Al Hussein,[a] Wesley R. Browne,[b] Emanuel Hupf,[a*] Jens Beckmann[a*]

[a] *Institute for Inorganic Chemistry and Crystallography, Faculty of Biology and Chemistry.*

University of Bremen, Leobener Straße 7, 28359 Bremen, Germany

[b]*Molecular Inorganic Chemistry, Stratingh Institute for Chemistry, Faculty of Science and Engineering, University of Groningen, Nijenborgh 4, 9747 Groningen, AG, The Netherlands*

[*] Correspondence to Emanuel Hupf (E-mail: emanuel.hupf@uni-bremen.de) and Jens Beckmann (E-mail: j.beckmann@uni-bremen.de)

Table of Contents

Experimental procedures 4
General information 4
1D NMR spectra 7
Fc_3As (3) 7
Fc_3Sb (4) 9
Fc_3Bi (5) 11
2D NMR spectra 13
UV-vis absorption spectroscopy 22
Cyclic Voltammetry 26
Spectroelectrochemistry 54
Crystallographic data 66
(TD)-DFT Computations 72
References 73

Experimental procedures

General information

Unless otherwise stated, all the reactions, manipulations work-up and purifications were performed under inert argon atmosphere using anhydrous solvents. The reagents used in this work including ferrocene, $AsCl_3$, and *n*-BuLi were obtained commercially and used as received. FcBr[S1], Fc_3P[S2], and [*n*-Bu_4N][$B(C_6F_5)_4$][S3] were prepared following the procedures described in the literature. Anhydrous dichloromethane, tetrahydrofuran, hexane, and acetonitrile were collected from an SPS800 mBraun solvent purification system and stored over 3 Å molecular sieves. Diethyl ether was dried by heating at reflux over Na/benzophenone under argon atmosphere. Deuterated solvents were degassed and dried over 3 Å molecular sieves under argon.

Unless otherwise noted, NMR spectra were recorded at room temperature on a Bruker Avance 600 MHz spectrometer. ^1H and $^{13}C\{^1H\}$ spectra are reported on the δ scale (ppm) and are referenced against $SiMe_4$. ^1H and $^{13}C\{^1H\}$ chemical shifts are reported to the residual peak of the solvent ($CDHCl_2$ 5.32 ppm for CD_2Cl_2) in the ^1H NMR spectra, and to the peak of the deuterated solvent (CD_2Cl_2 53.84 ppm) in the $^{13}C\{^1H\}$ NMR spectra.[S4] The assignment of the ^1H and $^{13}C\{^1H\}$ signals was made in accordance with the COSY, HMBC and HSQC spectra.

The ESI HRMS spectra were measured on a Bruker Impact II spectrometer. Dichloromethane, acetonitrile, or dichloromethane/acetonitrile solutions ($c = 1 \cdot 10^{-5}$ $mol \cdot L^{-1}$) were injected directly into the spectrometer at a flow rate of 3 $\mu L \cdot min^{-1}$. Nitrogen was used both as a drying gas and for nebulization with flow rates of approximately 5 $L \cdot min^{-1}$ and a pressure of 5 psi. Pressure in the mass analyser region was usually about $1 \cdot 10^{-5}$ mbar. Spectra were collected for 1 min and averaged. The nozzle-skimmer voltage was adjusted individually for each measurement.

UV-vis absorption spectra were recorded on a VWR UV-1600PC spectrophotometer, and analysed using Spectragryph software.[S5] UV-vis absorption spectra for the spectroelectrochemical experiments were recorded on a SPECORD 210 PLUS Double Beam UV-vis Spectrophotometer, Analytik Jena (± 0.1 nm accuracy). NIR spectra were recorded on a FTIR-4600 (0.7 cm resolution) Jasco spectrometer.

Spectroelectrochemical measurements were carried out on a Model 760c Electrochemical Workstation (CHInstruments).

For all electrochemical experiments spectroscopy grade solvents were employed. All the supporting electrolytes were used as received, except for [n-Bu_4N][$B(C_6F_5)_4$] (*vide supra*). Cyclic voltammetry studies were performed in a V-tube electrochemical cell. The CV data was recorded at room temperature (*ca.* 25 °C) and at −80 °C using a Autolab PGSTAT 101 (Metrohm) Electrochemical Workstation. A three-electrode configuration was used with a glassy carbon (CHInstruments CHI104) acting as working electrode (WE), a Pt wire acting as counter electrode (CE) and an Ag/AgCl pseudo reference electrode (pRE). Before every measurement, the counter electrode was polished to a mirror-like appearance with diamond paste (1µm) and carefully rinsed with deionized water, sonicated, and then rinsed with HPLC grade acetone. The decamethylferrocene/decamethylferrocenium redox couple ($Me_{10}Fc/Me_{10}Fc^+$, c = 1 mM) was used as internal standard for the measurements in CH_2Cl_2, except for the ones with [n-Bu_4N][$B(C_6F_5)_4$] (25 mM) as supporting electrolyte. In the latter arrangement, as well as in the measurements in acetonitrile, the cyclic voltammograms were referenced externally with $Me_{10}Fc/Me_{10}Fc^+$ redox couple (c = 0.25 mM). The analytes were measured as 1 mM solutions in CH_2Cl_2, and [n-Bu_4N][BF_4], [n-Bu_4N][PF_6], [n-Bu_4N][SbF_6] (0.1 M) were used as supporting electrolytes. The measurements in acetonitrile/dichloromethane (MeCN:CH_2Cl_2 10:1 V:V) employed lower concentrations of analytes (0.25 mM), and [n-Bu_4N][PF_6] (25 mM). The solutions were degassed with argon prior to measurement. The starting potential for all the CV measurements is – 0.1 V, while the direction of the initial scan is oxidative (goes to positive potentials). All the measurements were performed at the following scan rates: 50m/s, 100 mV/s, 125 mV/s and 250 mV/s. The IUPAC plotting convention was used for all CV illustrations.

Bulk electrolysis experiments were done under constant potential in a H-cell, divided by porous glass frit, granulate 3 (medium), while slowly purging argon through (using septa to keep oxygen and water out). Periodically, volumes of ~ 0.3 mL were taken out, transferred to 0.1 cm pathlength quartz cuvettes, and UV-vis spectra were recorded. The NIR measurements were performed in 1 cm pathlength quartz cuvettes.

Analytes' concentration of 5 mM in anhydrous CH_2Cl_2 containing 0.1 M [*n*-Bu_4N][PF_6] were prepared and degassed. A carbon mesh working electrode, platinum wire counter electrode, and Ag/AgCl pseudo reference electrode were employed. The bulk electrolysis voltages were as follows: 1.0 V for Fc_3P (**2**), 0.75V for Fc_3As (**3**), 0.70 V for Fc_3Sb (**4**) and 0.60 V for Fc_3Bi (**5**). The redox processes (oxidation followed by reduction) were also followed in the same set-up, this time using the AvaSpec-ULS2048CL-EVO-RS-UA to evaluate the changes in the visible region.

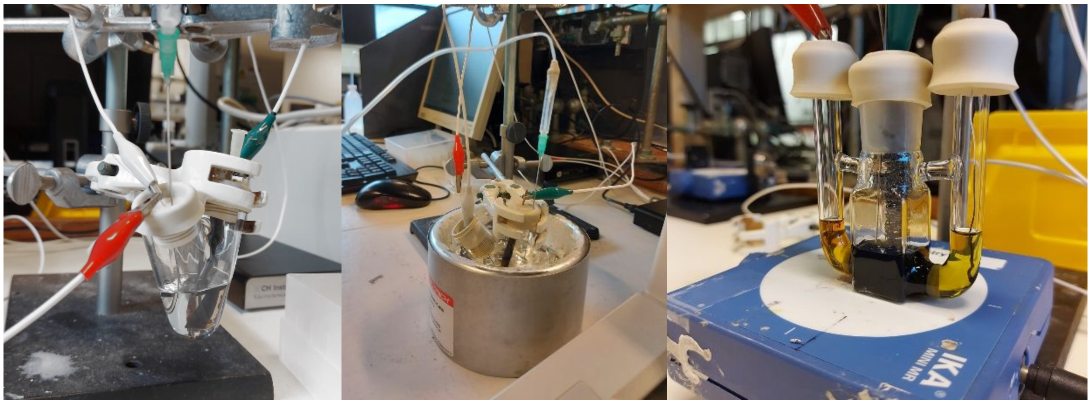

Figure S1. From left to right: a) cyclic voltammetry (CV) set up; b) low temperature CV set up; c) bulk electrolysis set up.

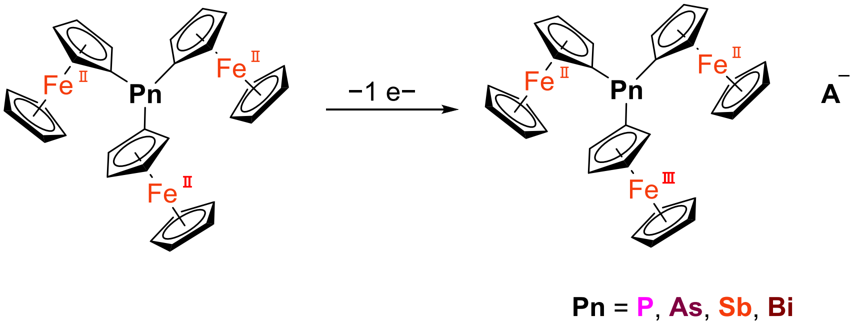

Figure S2. Targeted compounds for one-electron oxidation.

1D NMR spectra

Fc_3As (3)

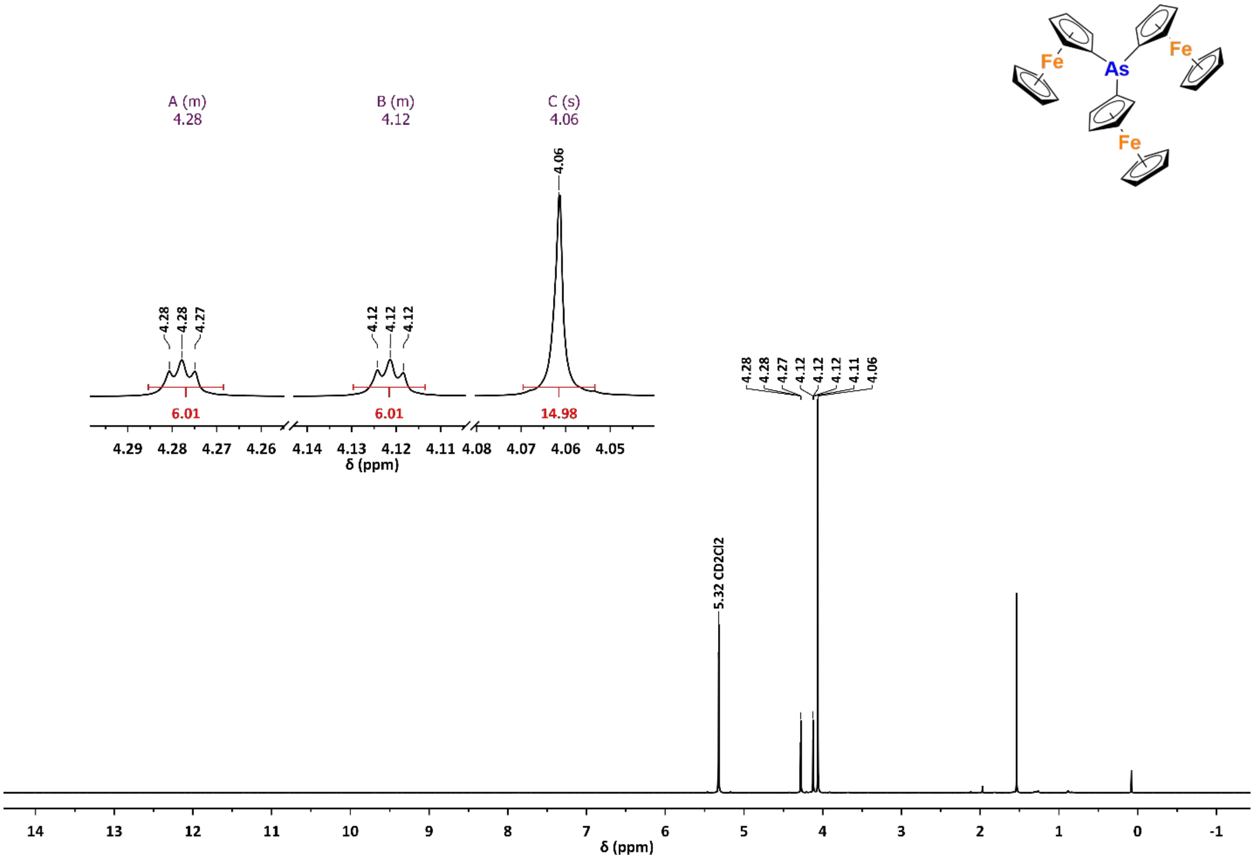

Figure S3. ^1H NMR (CD_2Cl_2, 600 MHz) spectrum of **3**.

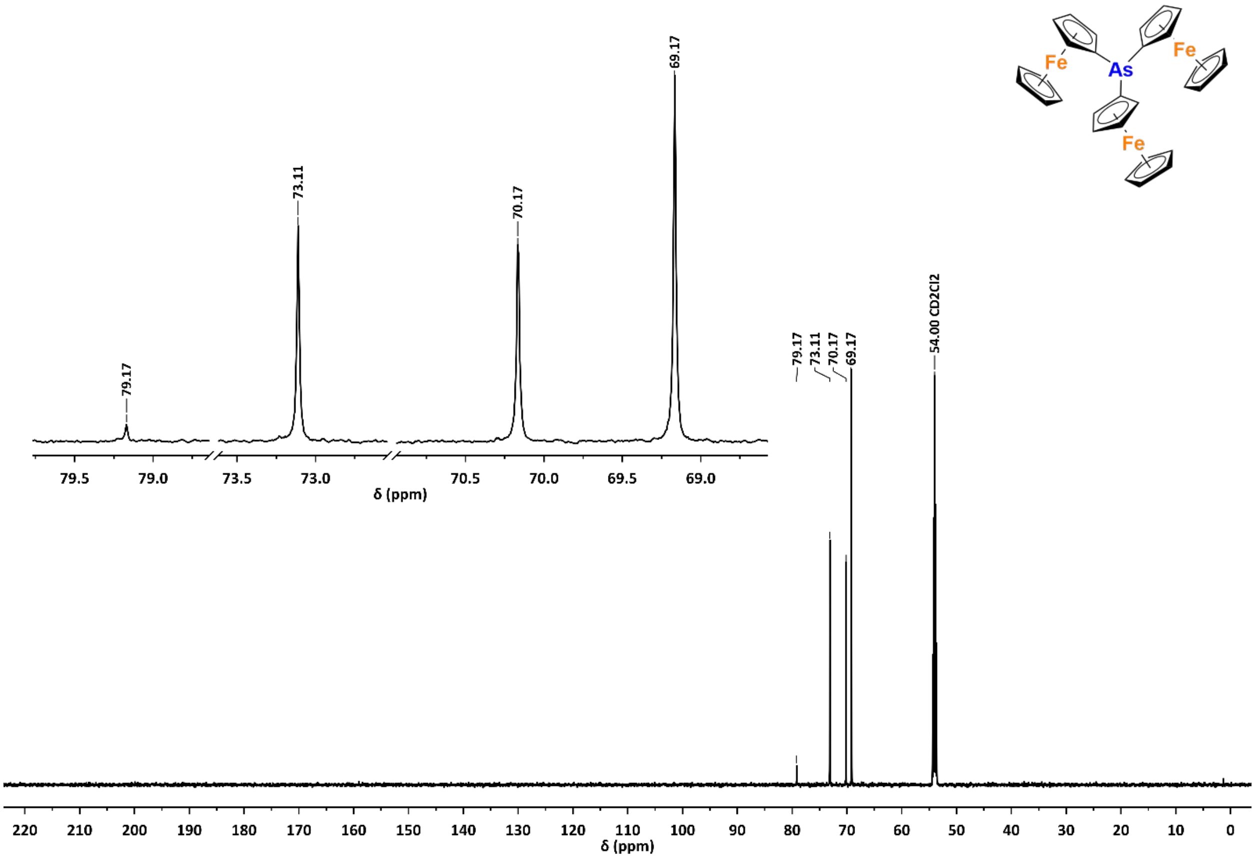

Figure S4. $^{13}C\{^1H\}$ NMR (CD_2Cl_2, 151 MHz) spectrum of **3**.

Fc_3Sb (4)

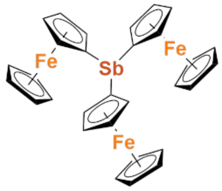

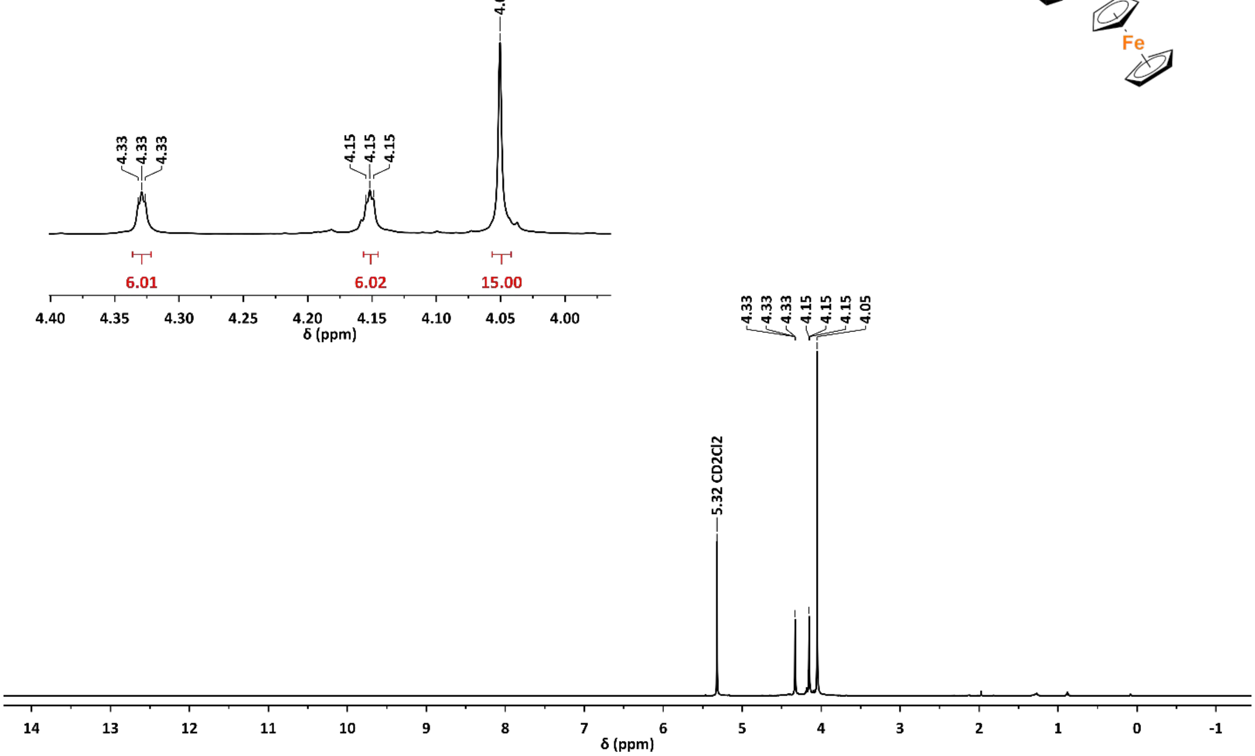

Figure S5. ^1H NMR (CD_2Cl_2, 600 MHz) spectrum of **4**.

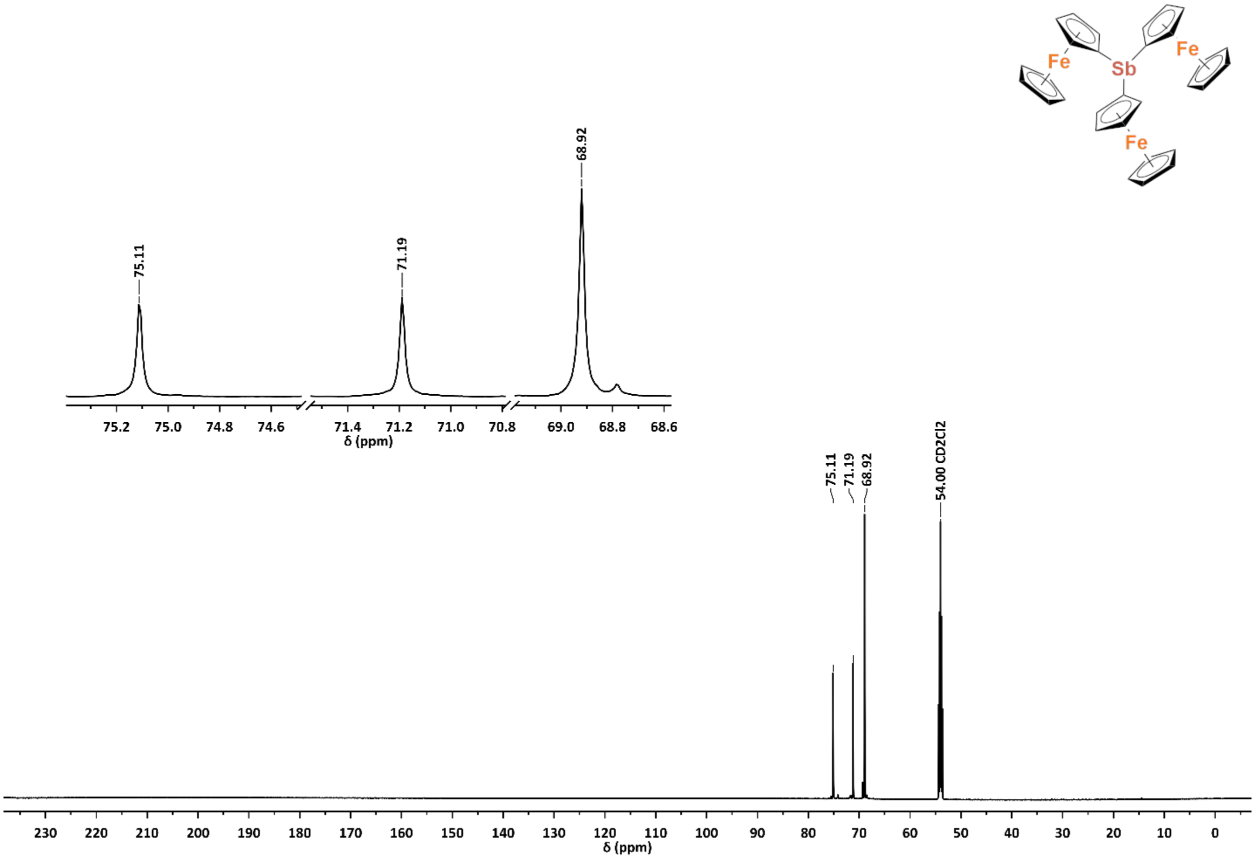

Figure S6. $^{13}C\{^{1}H\}$ NMR (CD_2Cl_2, 151 MHz) spectrum of **4**.

Fc_3Bi (5)

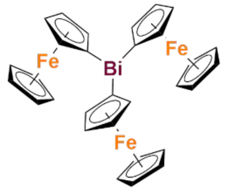

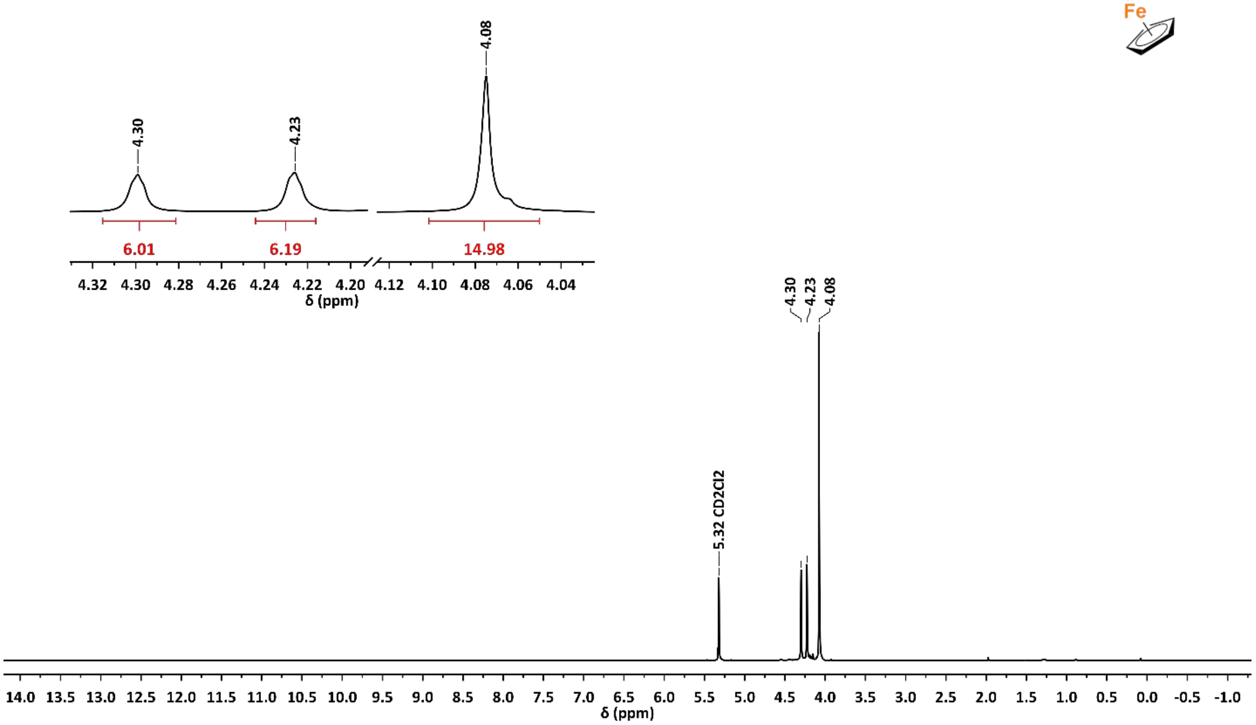

Figure S7. ^1H NMR (CD_2Cl_2, 600 MHz) spectrum of **5**.

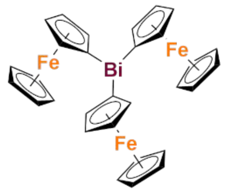

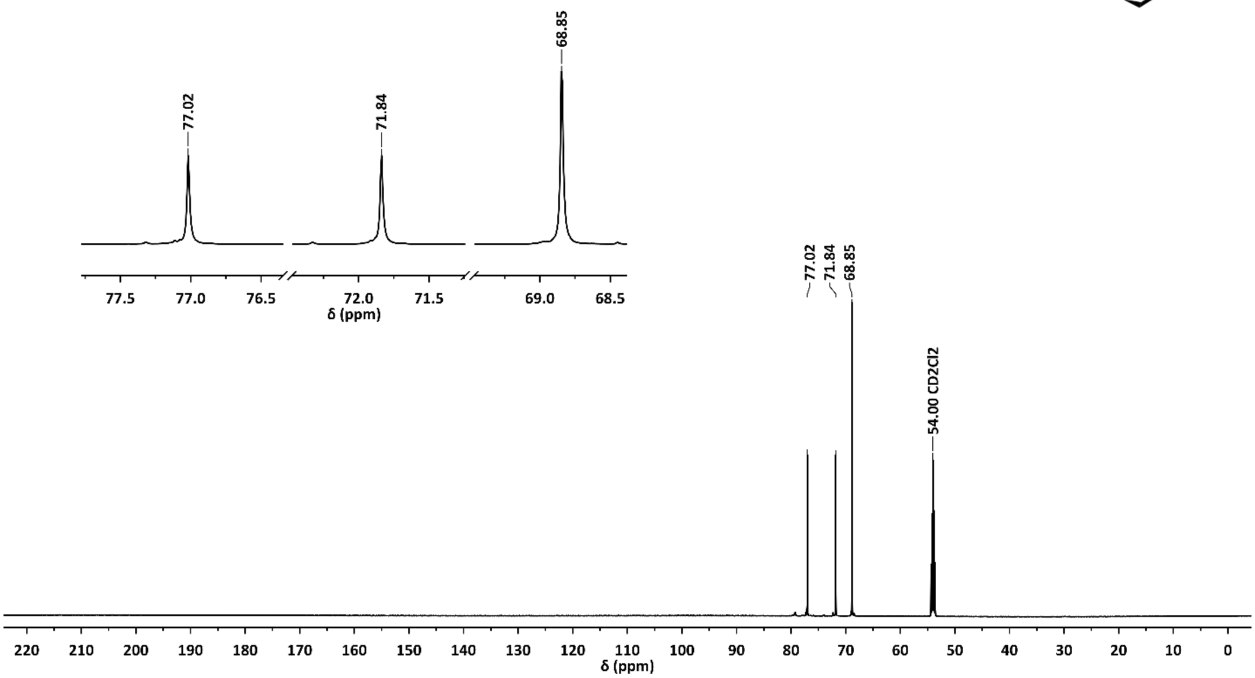

Figure S8. $^{13}C\{^{1}H\}$ NMR (CD_2Cl_2, 151 MHz) spectrum of **5**.

2D NMR spectra

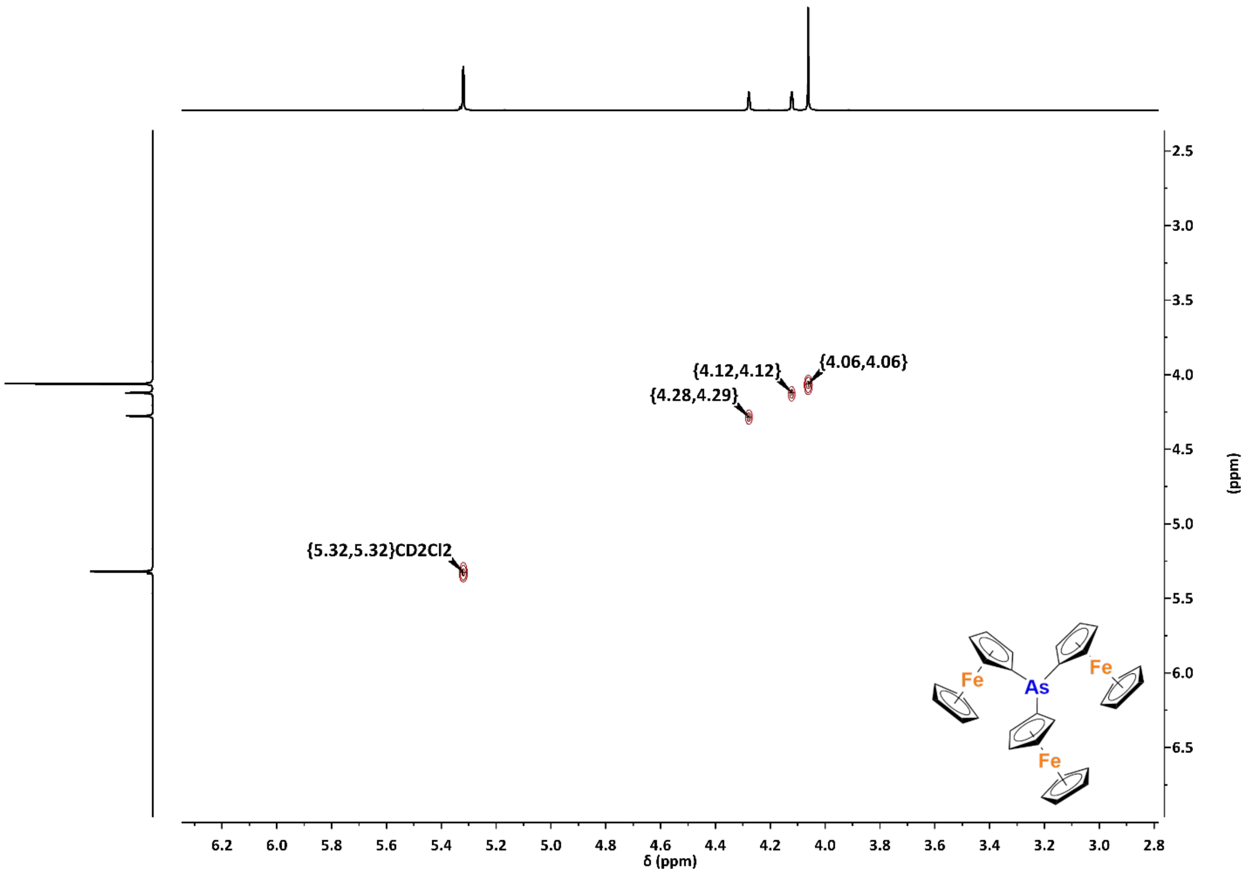

Figure S9. COSY NMR (CD_2Cl_2) spectrum of **3**.

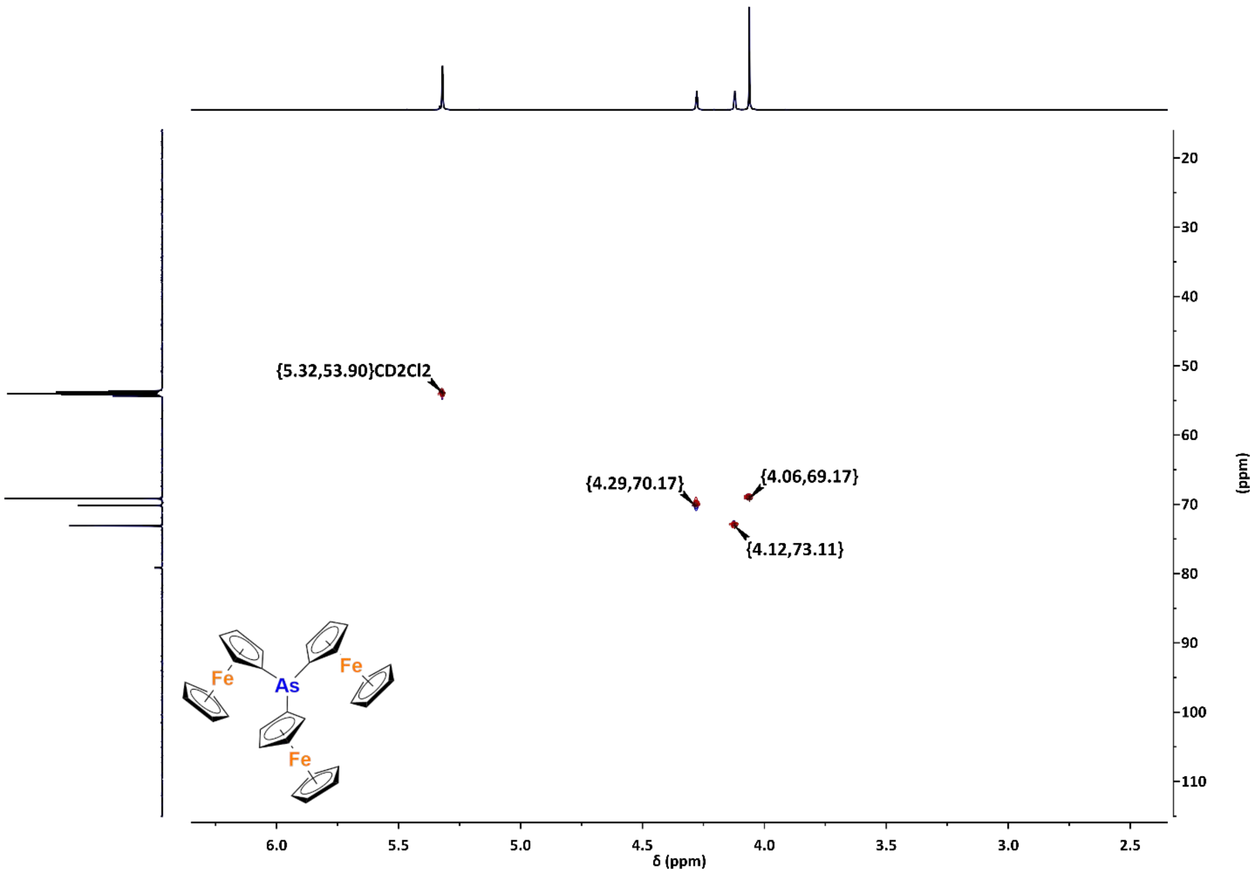

Figure S10. HSQC NMR (CD_2Cl_2) spectrum of **3**.

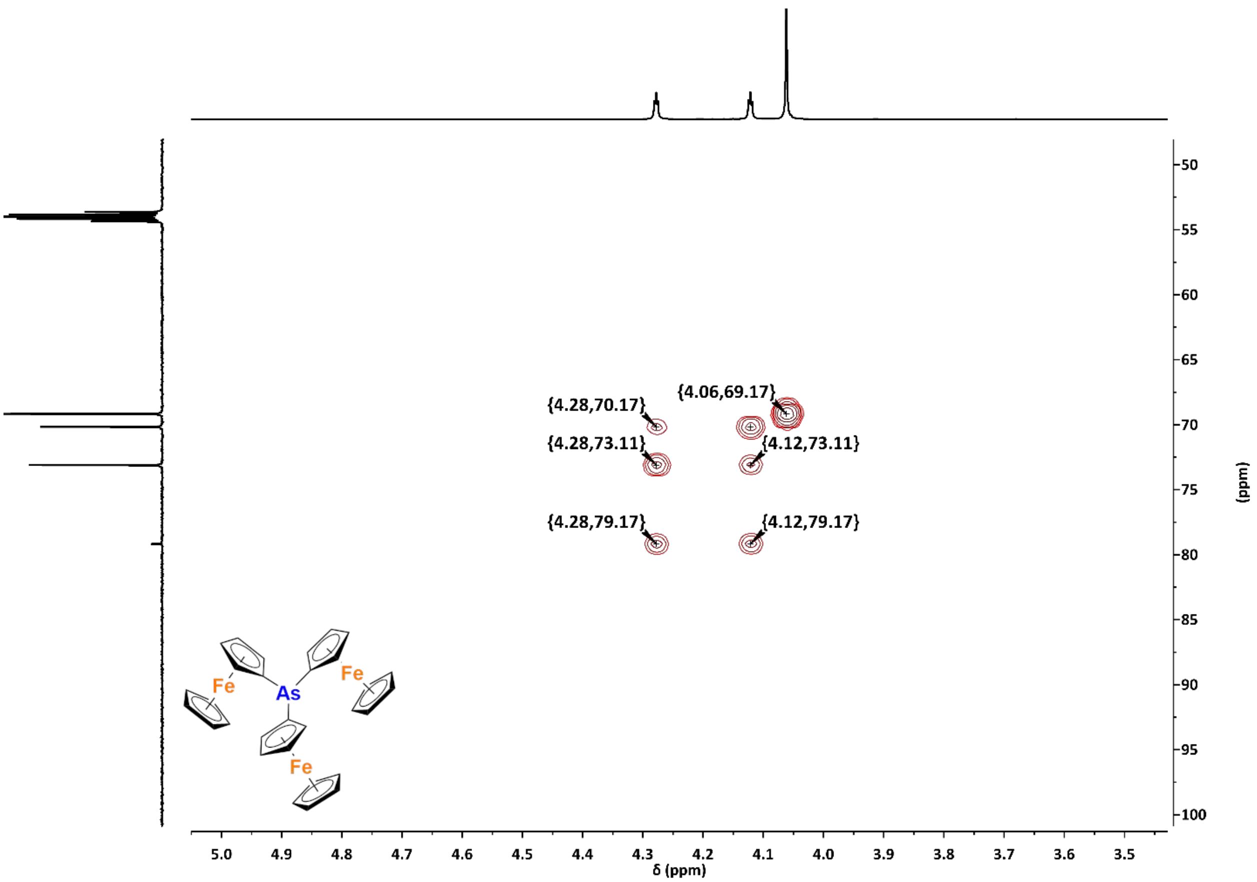

Figure S11. HMBC NMR (CD_2Cl_2) spectrum of **3**.

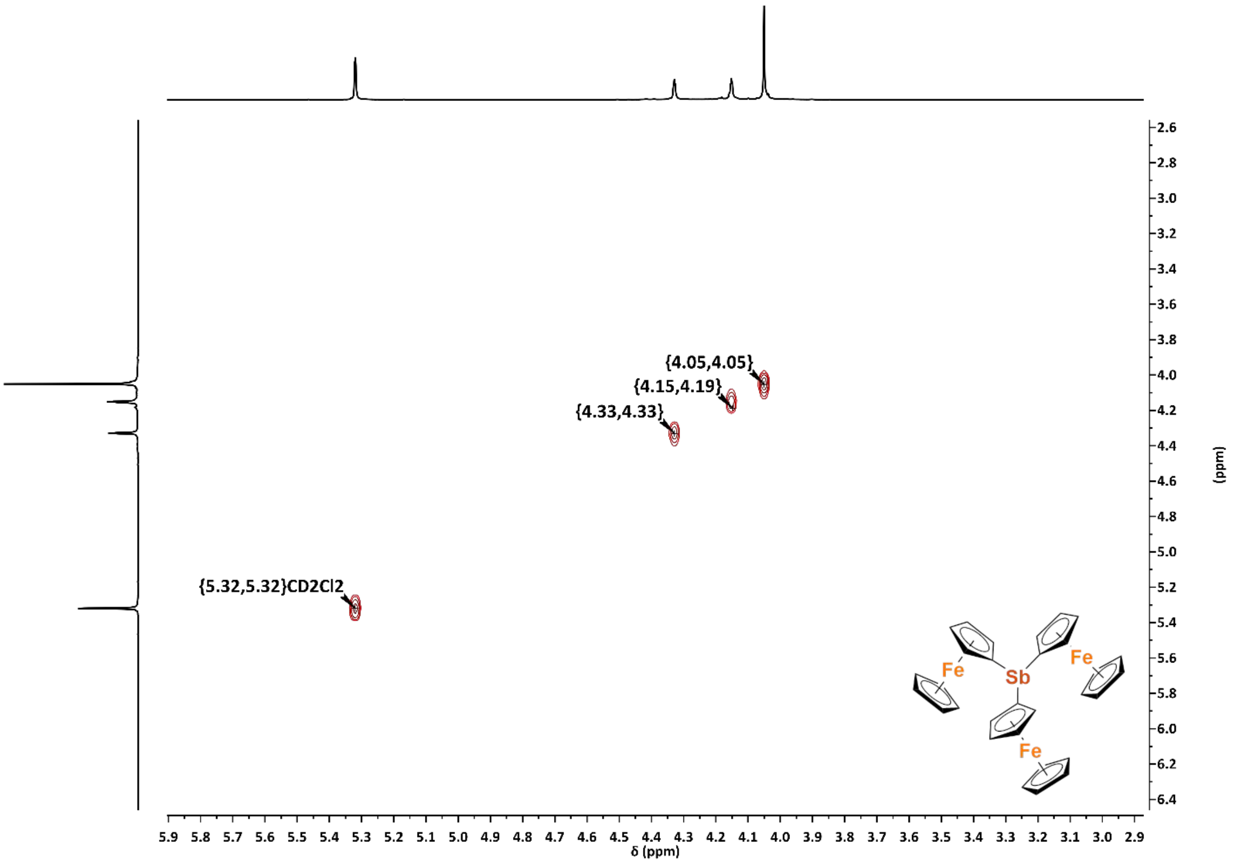

Figure S12. COSY NMR (CD_2Cl_2) spectrum of **4**.

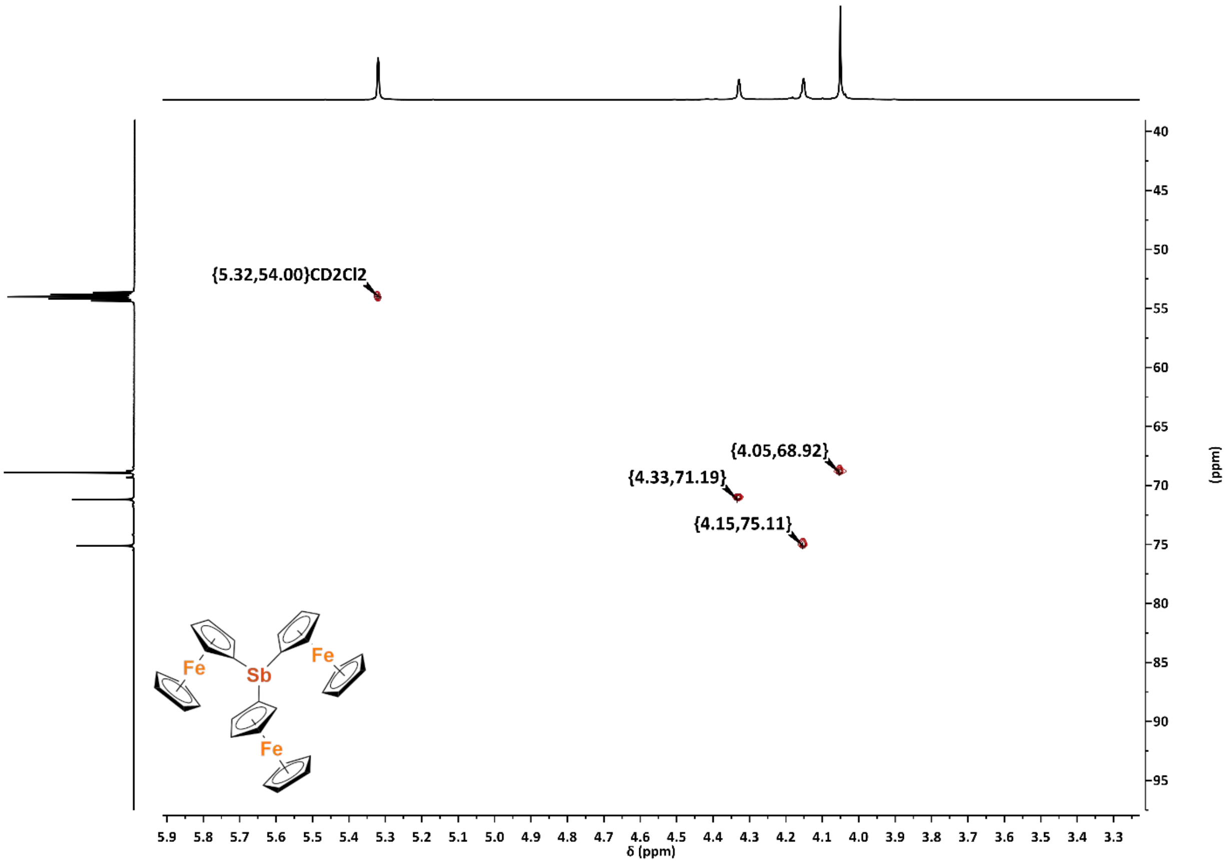

Figure S13. HSQC NMR (CD_2Cl_2) spectrum of **4**.

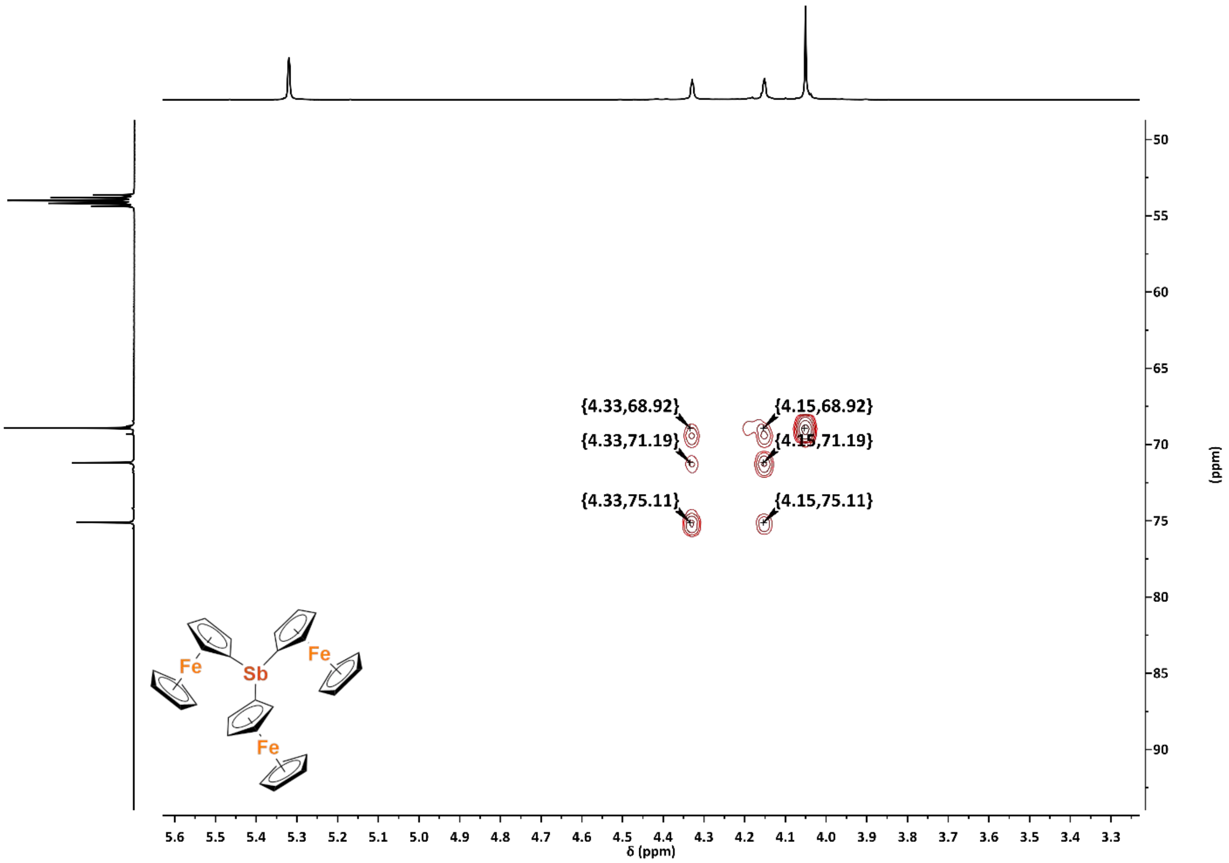

Figure S14. HMBC NMR (CD_2Cl_2) spectrum of **4**.

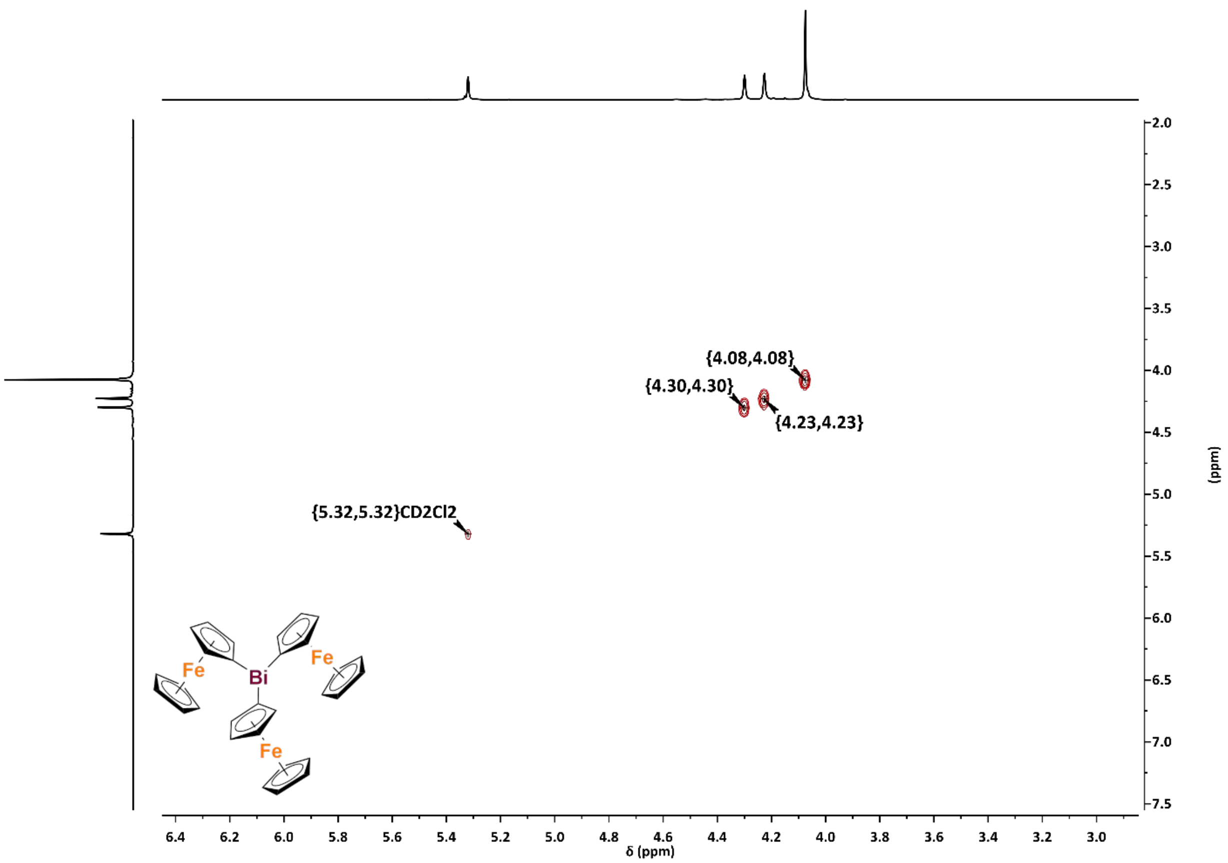

Figure S15. COSY NMR (CD_2Cl_2) spectrum of **5**.

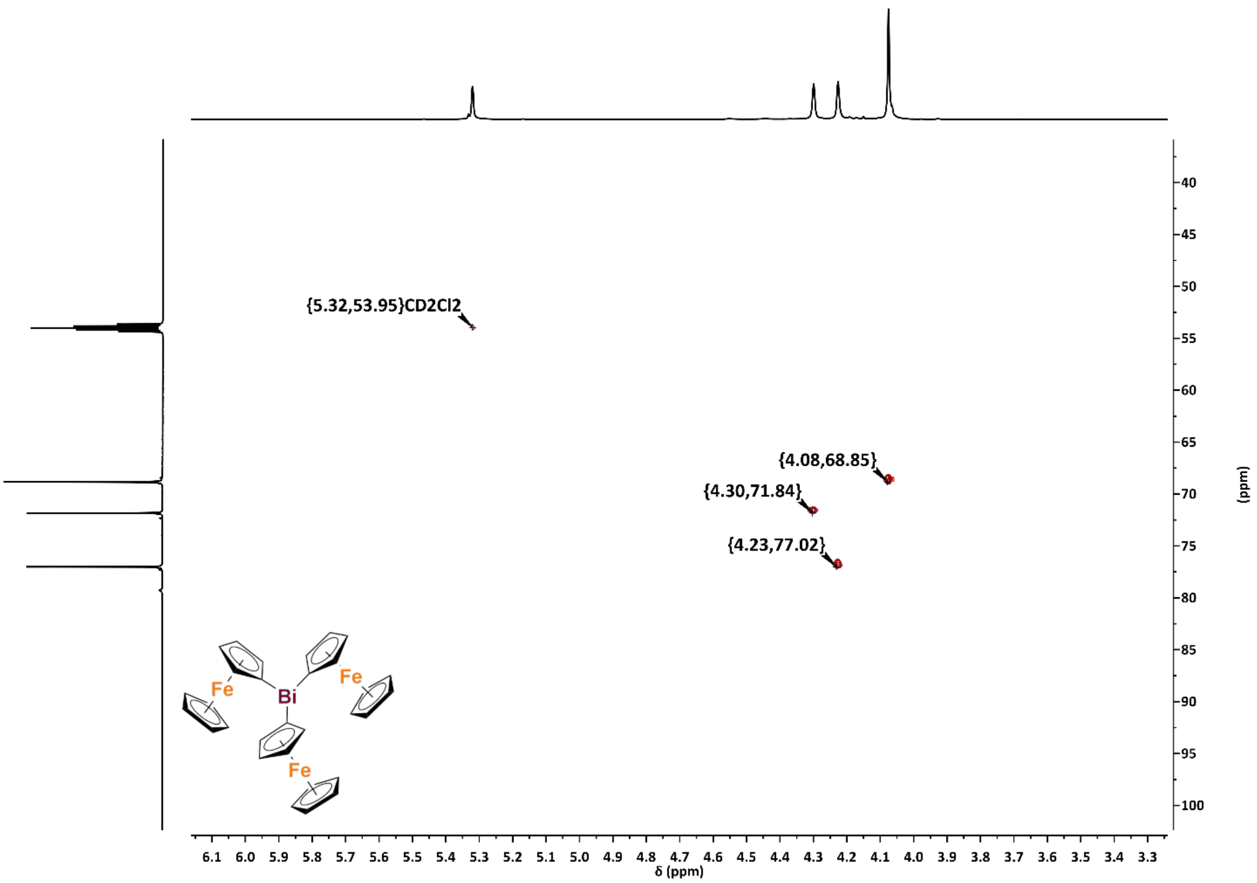

Figure S16. HSQC NMR (CD_2Cl_2) spectrum of **5**.

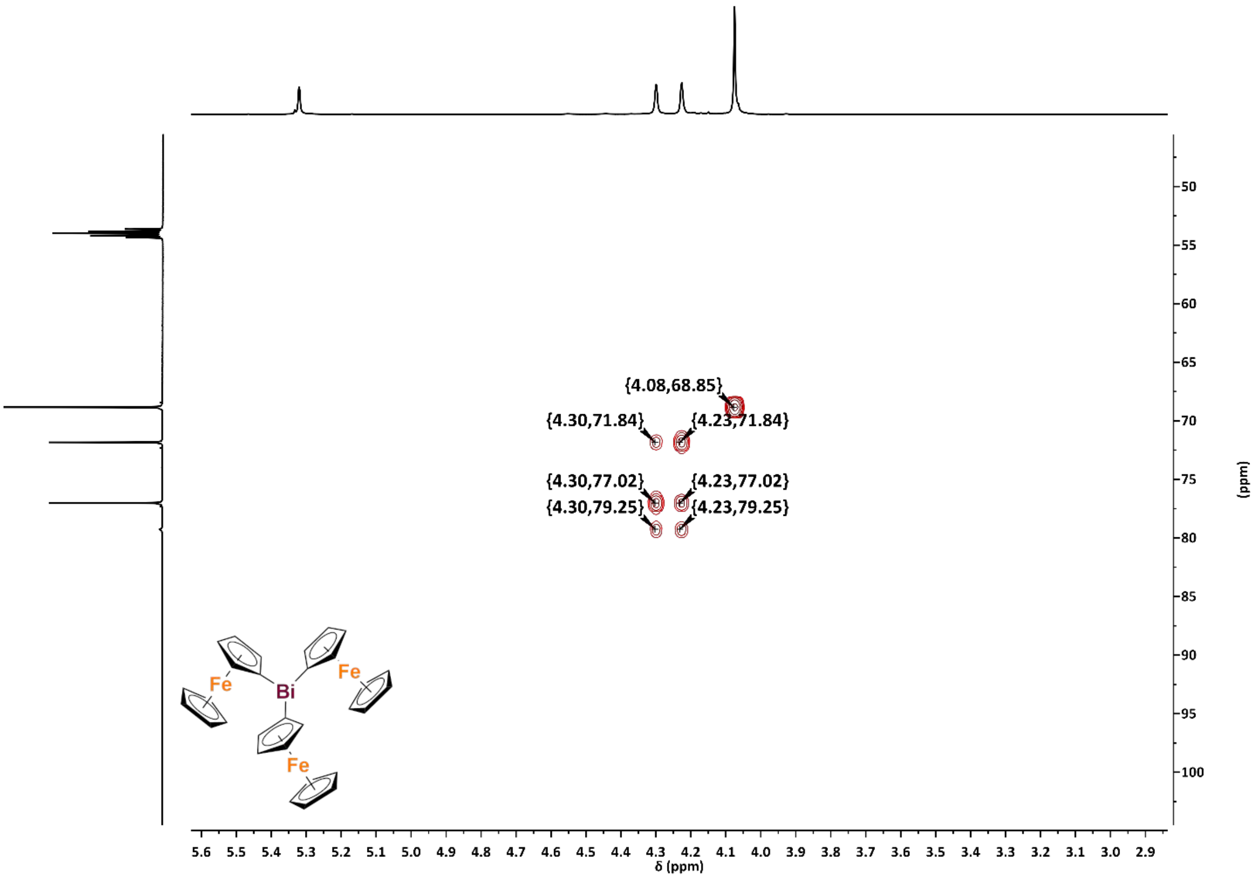

Figure S17. HMBC NMR (CD_2Cl_2) spectrum of **5**.

UV-vis absorption spectroscopy

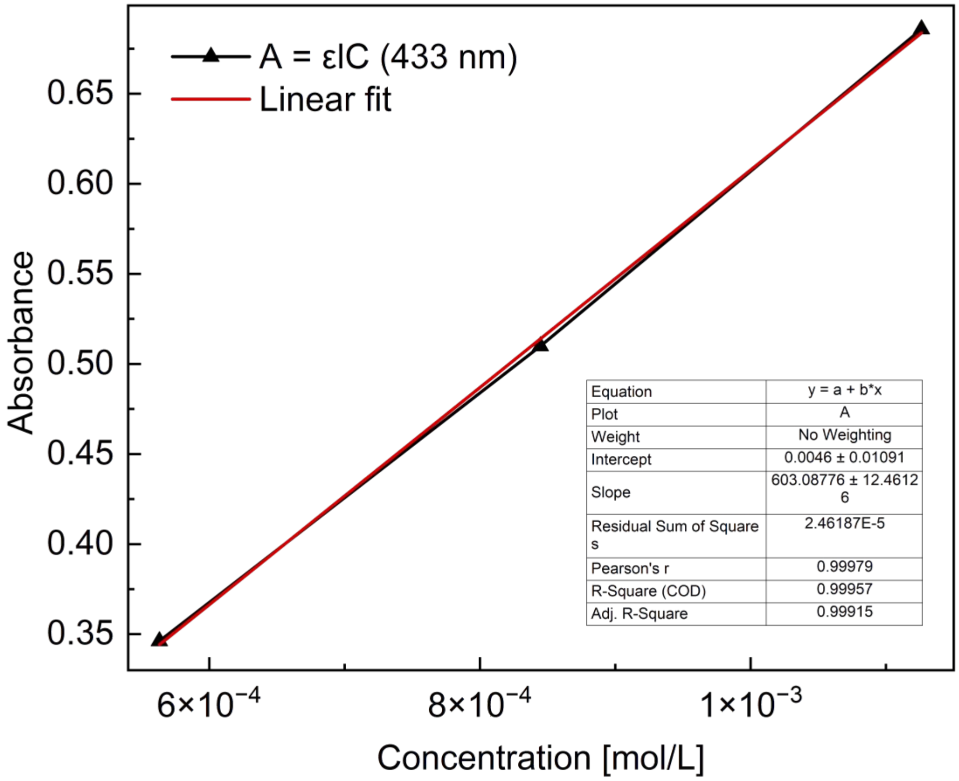

Figure S18. Concentration–absorbance dependence at 433 nm, absorption maximum of Fc_3P (**2**) in CH_2Cl_2. Molar absorptivity in the presented range (ε_{433} = 603 cm^{-1} M^{-1}) was determined using the Lambert–Beer law.

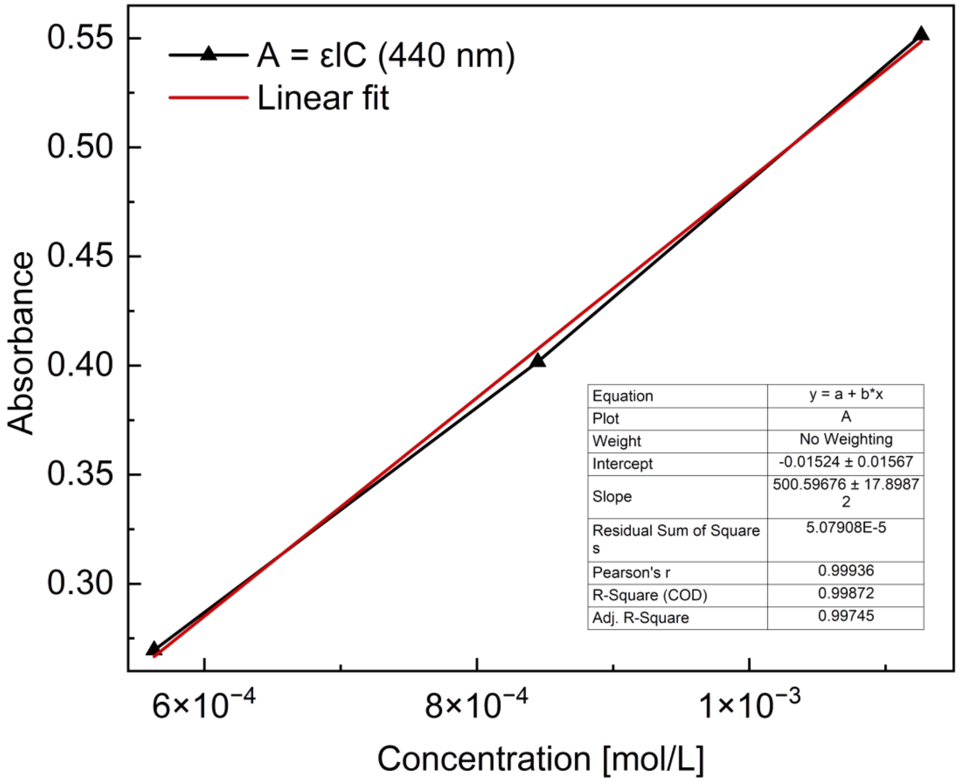

Figure S19. Concentration–absorbance dependence at 440 nm, absorption maximum of Fc_3As (**3**) in CH_2Cl_2. Molar absorptivity in the presented range (ε_{440} = 501 cm^{-1} M^{-1}) was determined using the Lambert–Beer law.

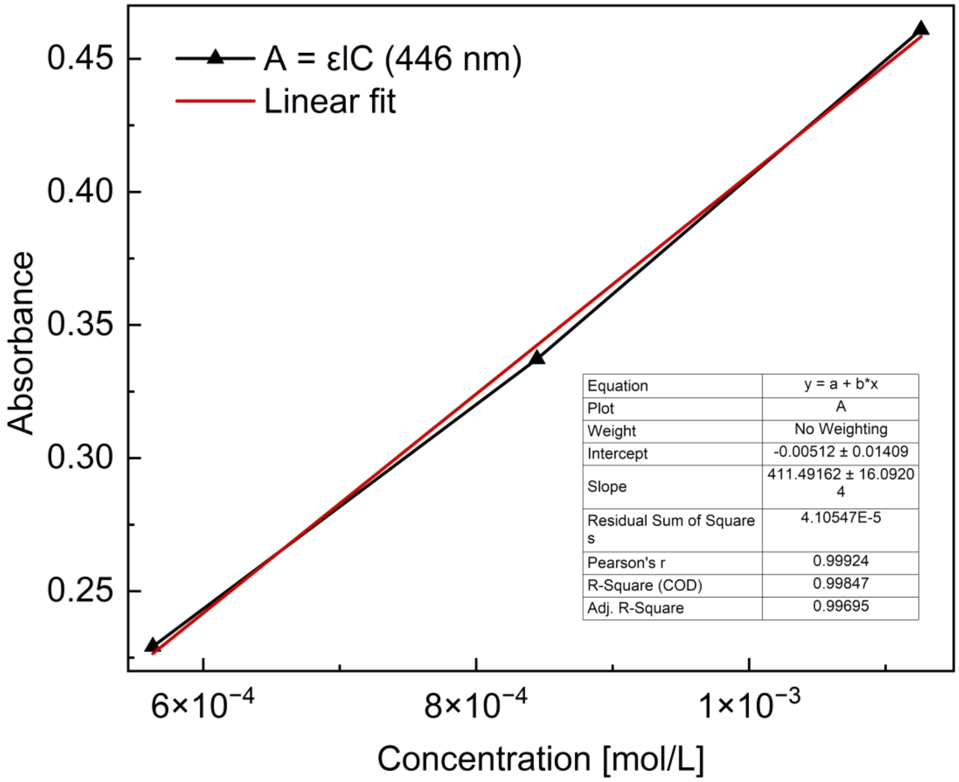

Figure S20. Concentration–absorbance dependence at 446 nm, absorption maximum of Fc_3Sb (**4**) in CH_2Cl_2. Molar absorptivity in the presented range (ε_{446} = 411 cm^{-1} M^{-1}) was determined using the Lambert–Beer law.

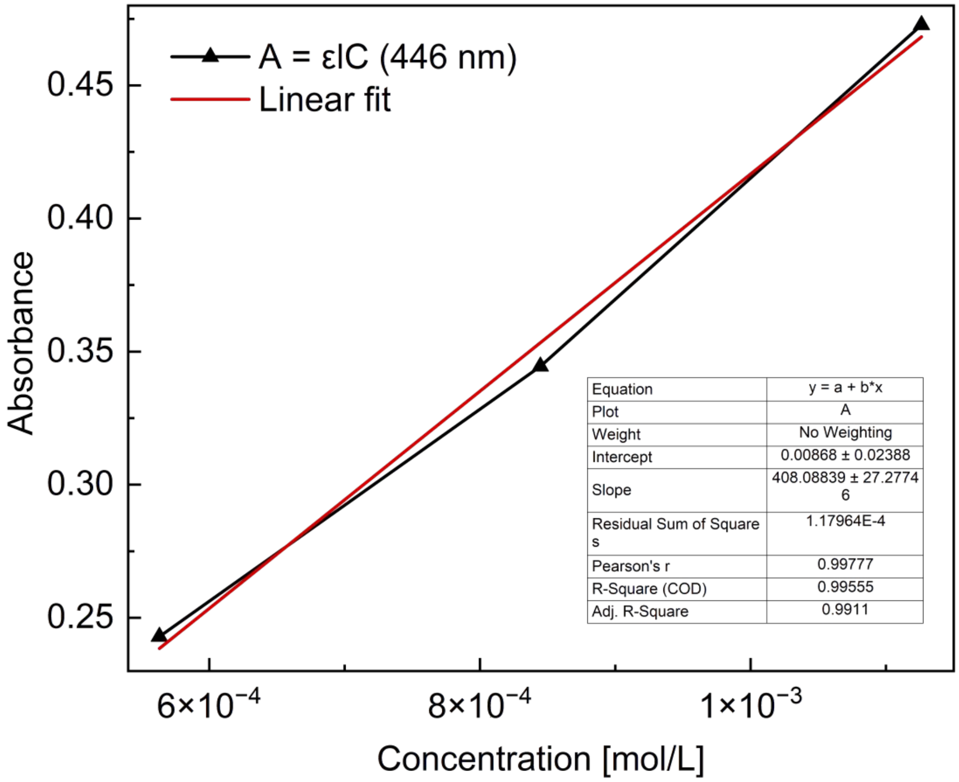

Figure S21. Concentration–absorbance dependence at 446 nm, absorption maximum of Fc_3Bi (**5**) in CH_2Cl_2.Molar absorptivity in the presented range (ε_{446} = 408 cm^{-1} M^{-1}) was determined using the Lambert–Beer law.

Cyclic Voltammetry

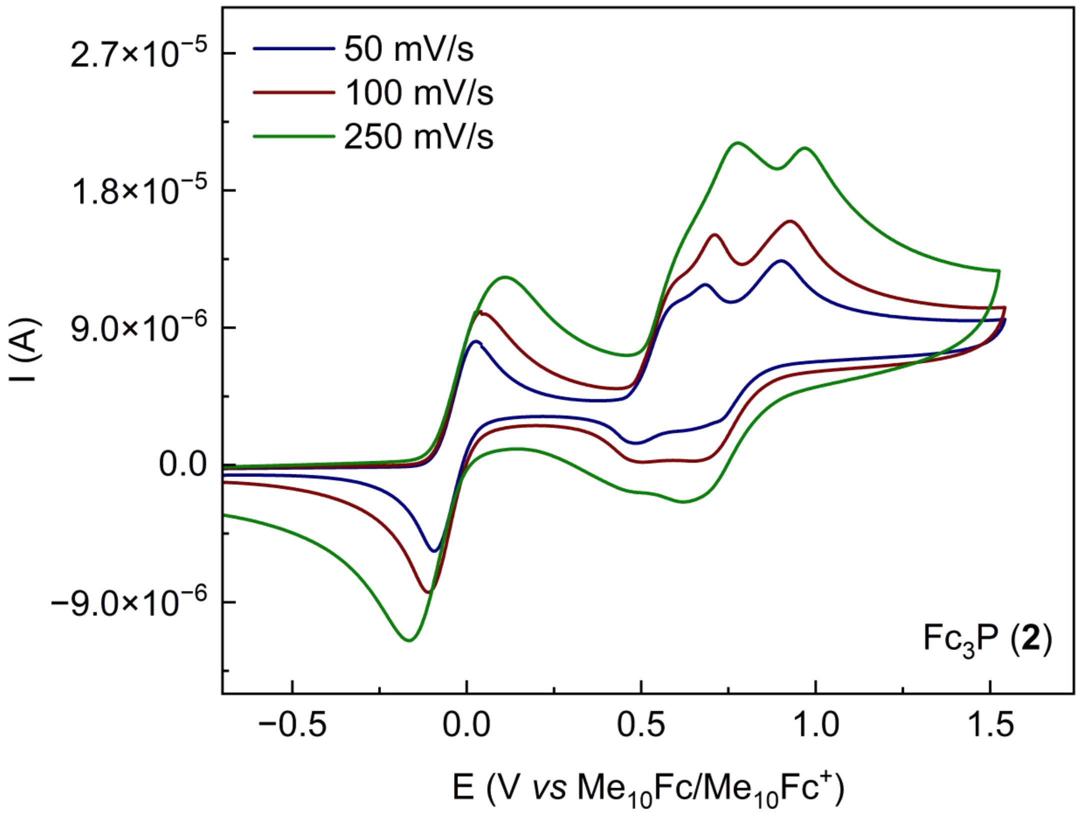

Figure S22. Cyclic voltammograms of **2** (1 mM) in **dichloromethane** at −80 °C (**193 K**), [*n*-Bu_4N][**BF_4**] (0.1 M) as supporting electrolyte and different scan rates (50 mV/s, 100 mV/s, and 250 mV/s), potential reported against $Me_{10}Fc/Me_{10}Fc^+$ ($Me_{10}Fc$ was added to the solution).

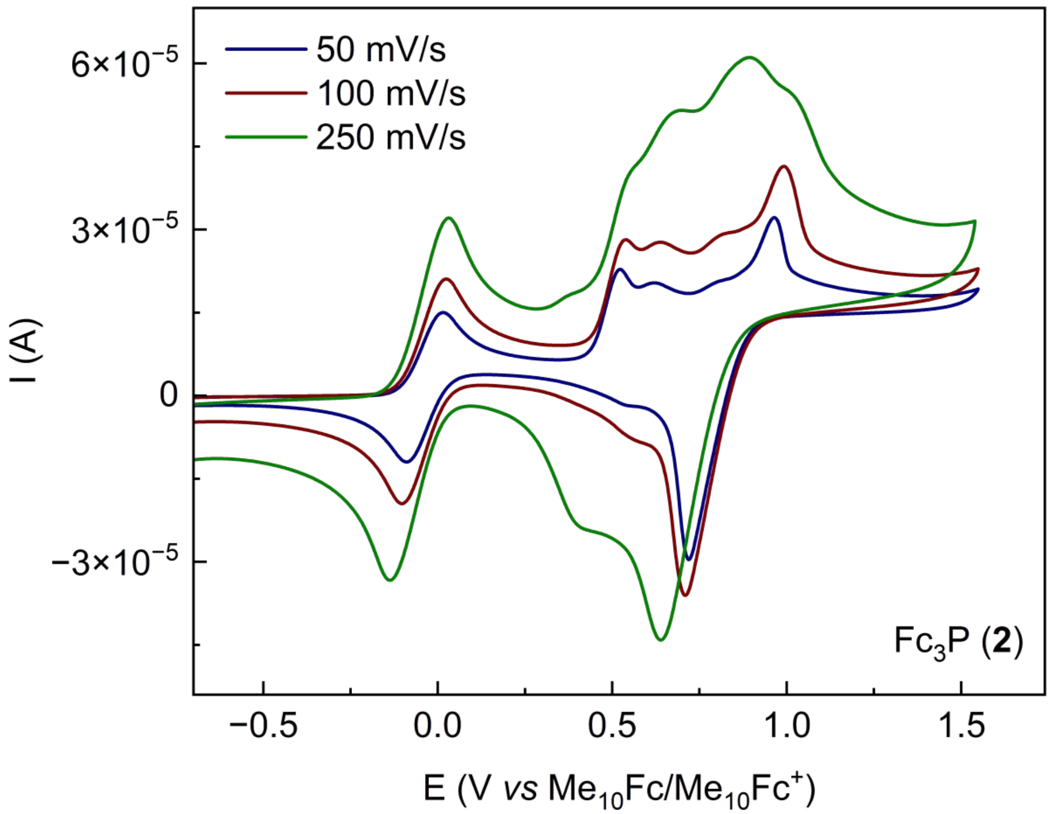

Figure S23. Cyclic voltammograms of **2** (1 mM) in **dichloromethane** at room temperature (**298 K**), [*n*-Bu_4N][$\mathbf{BF_4}$] (0.1 M) as supporting electrolyte and different scan rates (50 mV/s, 100 mV/s, and 250 mV/s), potential reported against $Me_{10}Fc/Me_{10}Fc^+$ ($Me_{10}Fc$ was added to the solution).

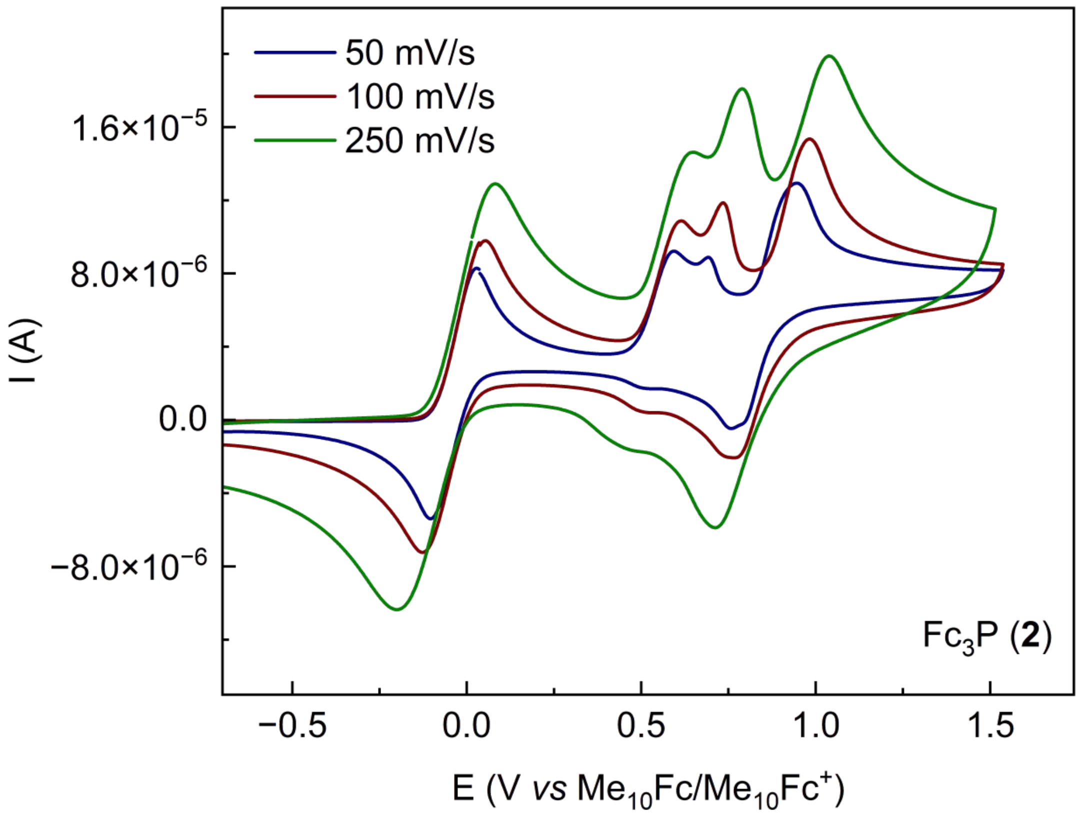

Figure S24. Cyclic voltammograms of **2** (1 mM) in **dichloromethane** at −80 °C (**193 K**), [*n*-Bu_4N][**PF_6**] (0.1 M) as supporting electrolyte and different scan rates (50 mV/s, 100 mV/s, and 250 mV/s), potential reported against $Me_{10}Fc/Me_{10}Fc^+$ ($Me_{10}Fc$ was added to the solution).

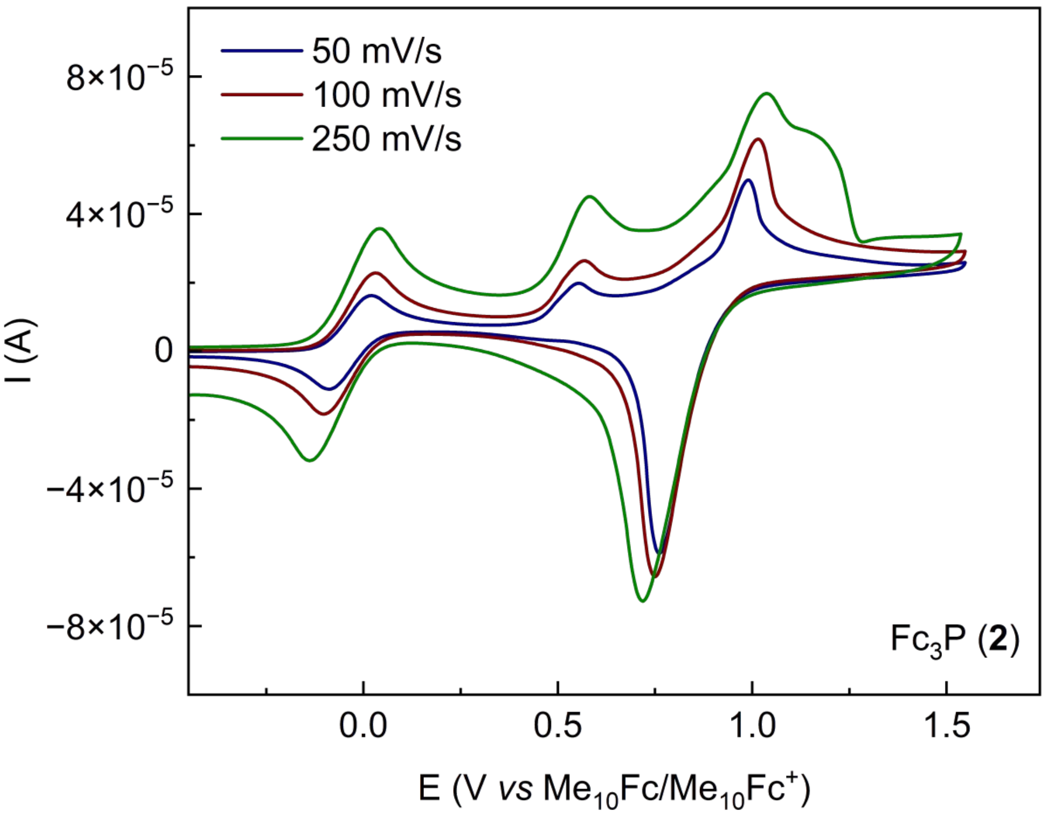

Figure S25. Cyclic voltammograms of **2** (1 mM) in **dichloromethane** at room temperature (**298 K**), [*n*-Bu_4N][$\mathbf{PF_6}$] (0.1 M) as supporting electrolyte and different scan rates (50 mV/s, 100 mV/s, and 250 mV/s), potential reported against $Me_{10}Fc/Me_{10}Fc^+$ ($Me_{10}Fc$ was added to the solution).

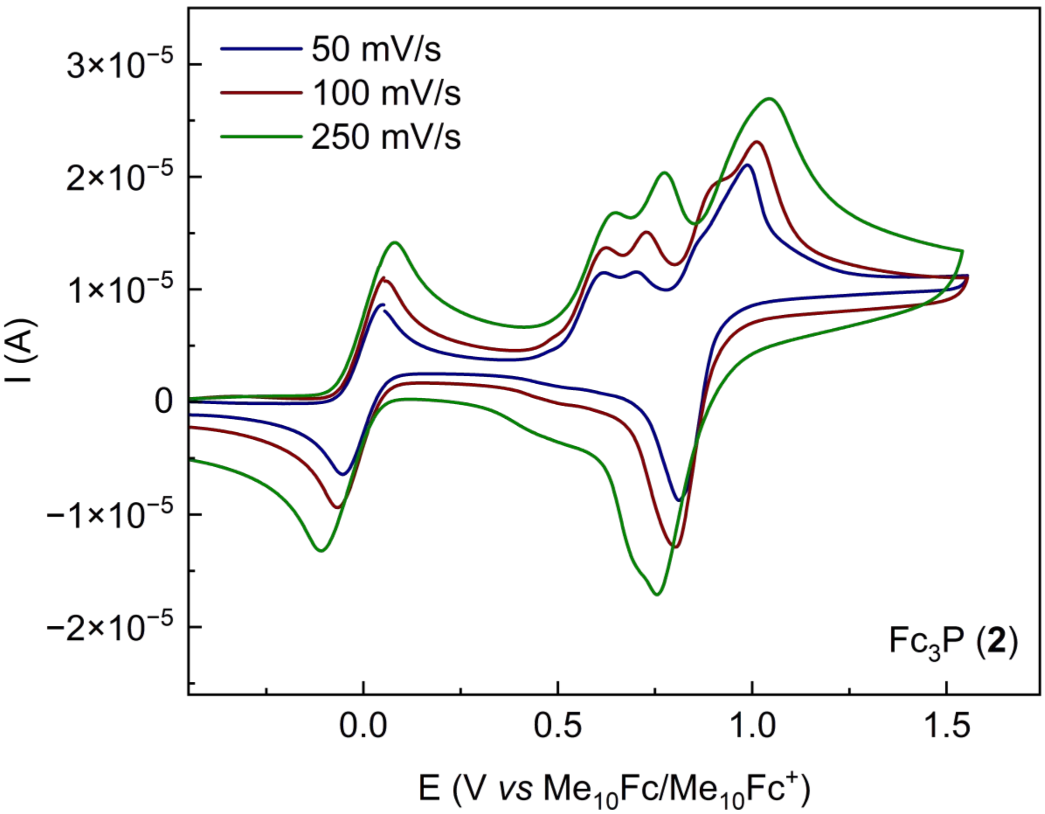

Figure S26. Cyclic voltammograms of **2** (1 mM) in **dichloromethane** at −80 °C (**193 K**), [*n*-Bu_4N][$\mathbf{SbF_6}$] (0.1 M) as supporting electrolyte and different scan rates (50 mV/s, 100 mV/s, and 250 mV/s), potential reported against $Me_{10}Fc/Me_{10}Fc^+$ ($Me_{10}Fc$ was added to the solution).

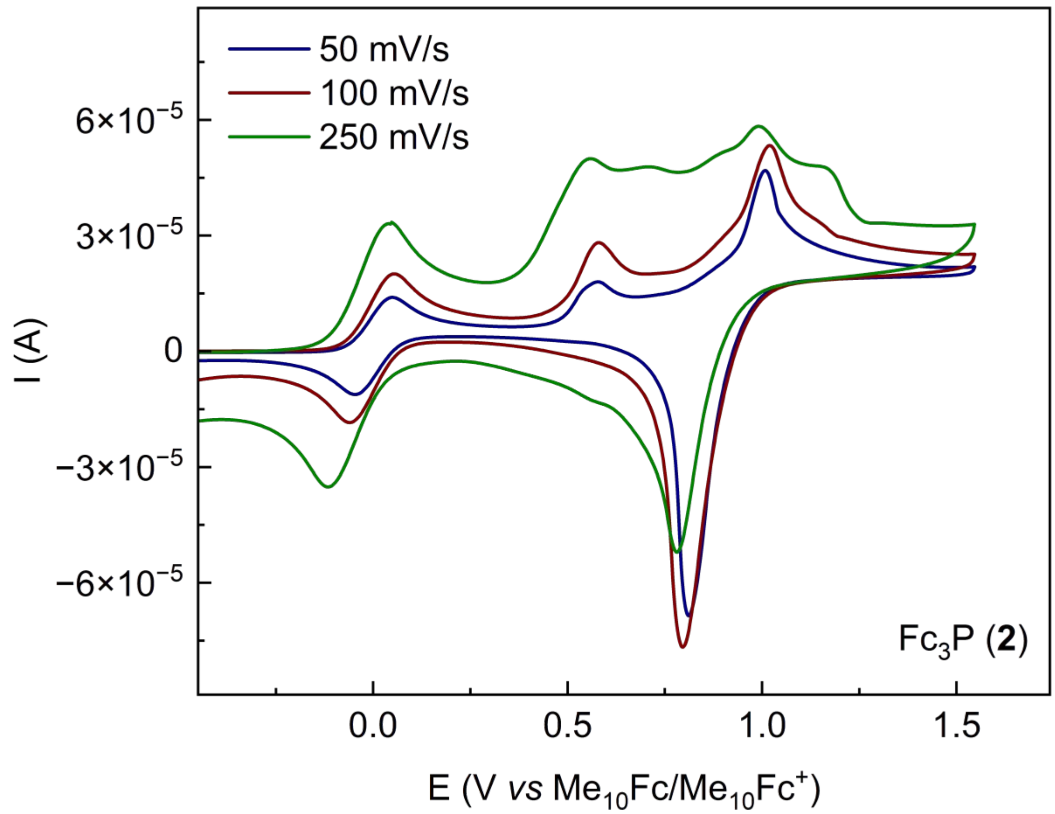

Figure S27. Cyclic voltammograms of **2** (1 mM) in **dichloromethane** at room temperature (**298 K**), [*n*-Bu_4N][**SbF_6**] (0.1 M) as supporting electrolyte and different scan rates (50 mV/s, 100 mV/s, and 250 mV/s), potential reported against $Me_{10}Fc/Me_{10}Fc^+$ ($Me_{10}Fc$ was added to the solution).

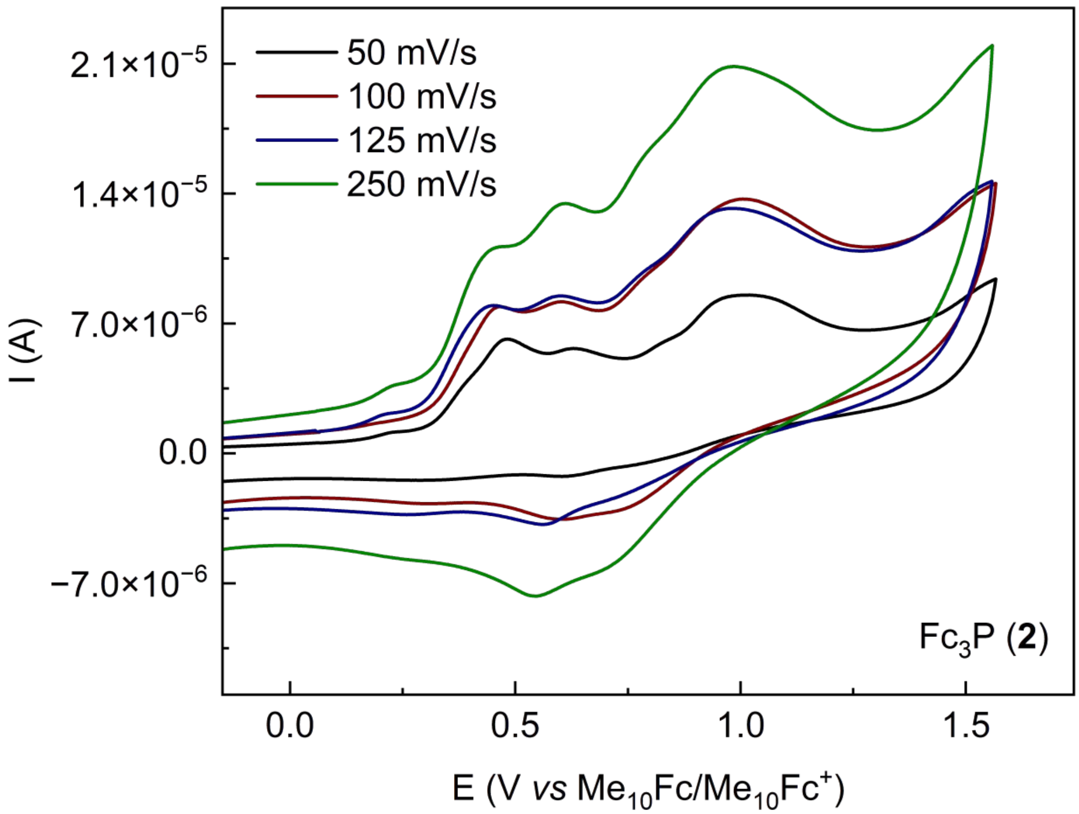

Figure S28. Cyclic voltammograms of **2** (0.25 mM) in **MeCN:CH_2Cl_2** (10:1, V:V) at room temperature (**298 K**), [*n*-Bu_4N][**PF_6**] (25 mM) as supporting electrolyte and different scan rates (50 mV/s, 100 mV/s, and 250 mV/s), potential reported against $Me_{10}Fc/Me_{10}Fc^+$.

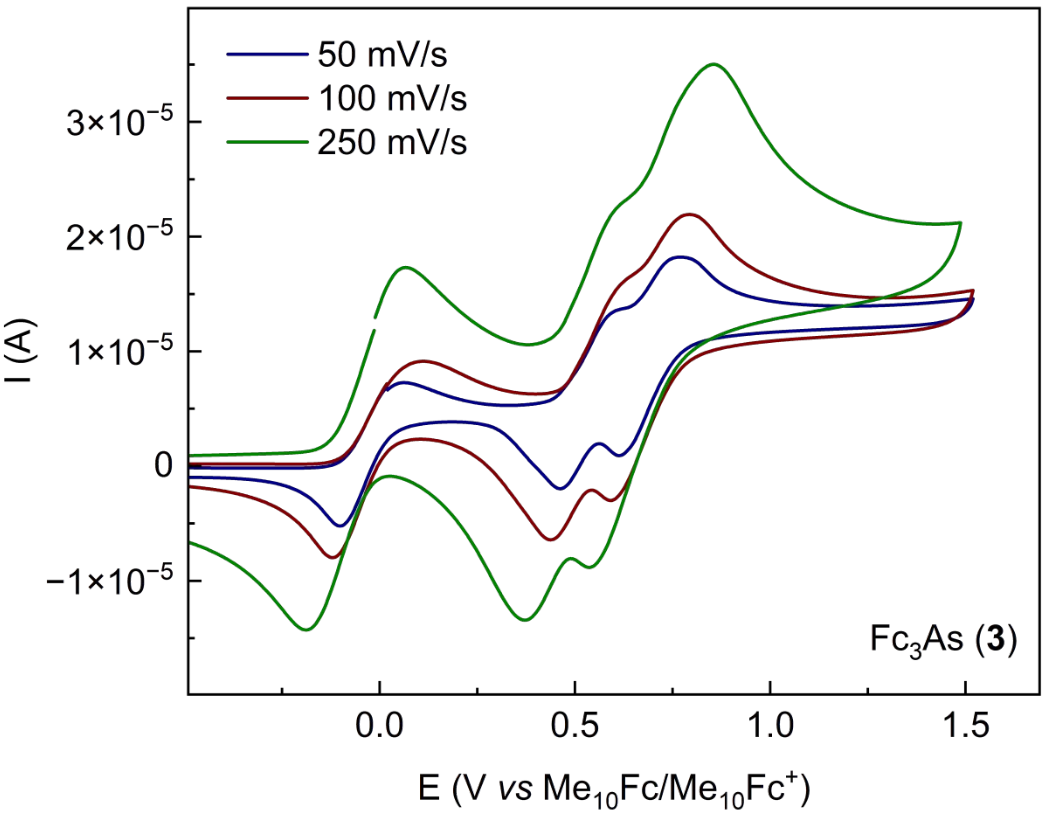

Figure S29. Cyclic voltammograms of **3** (1 mM) in **dichloromethane** at −80 °C (**193 K**) [*n*-Bu_4N][$\mathbf{BF_4}$] (0.1 M) as supporting electrolyte and different scan rates (50 mV/s, 100 mV/s, and 250 mV/s), potential reported against $Me_{10}Fc/Me_{10}Fc^+$ ($Me_{10}Fc$ was added to the solution).

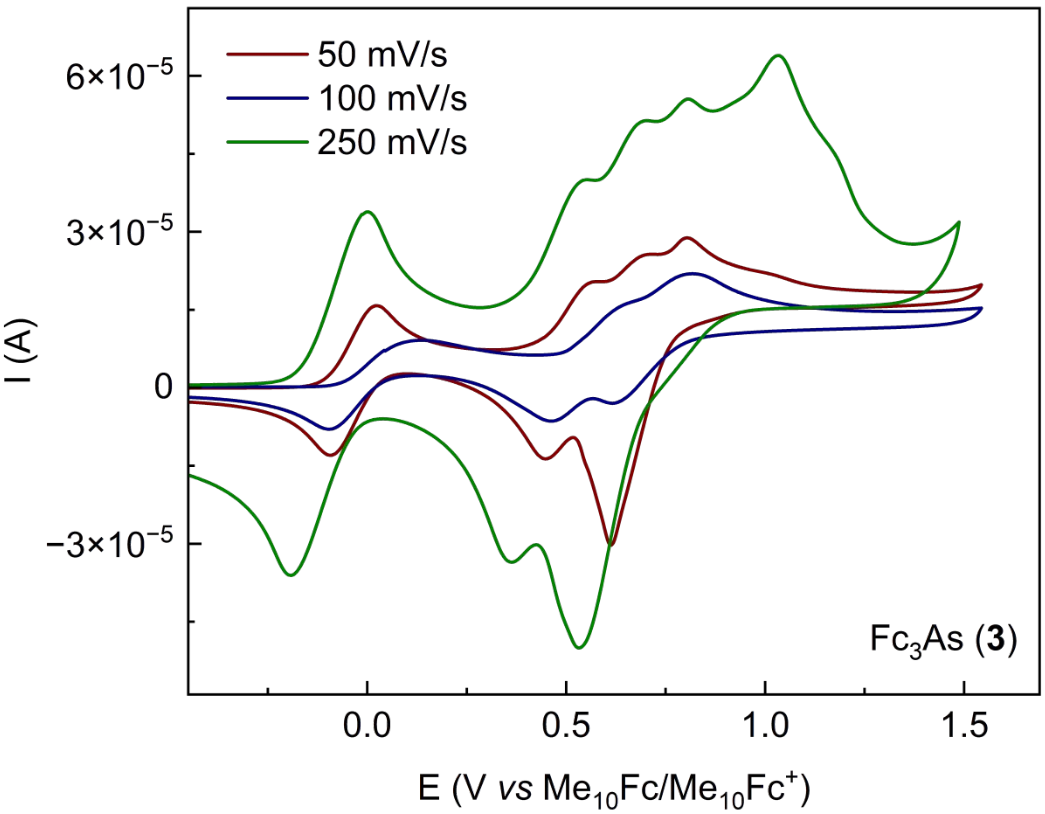

Figure S30. Cyclic voltammograms of **3** (1 mM) in **dichloromethane** at room temperature (**298 K**), [*n*-Bu_4N][$\mathbf{BF_4}$] (0.1 M) as supporting electrolyte and different scan rates (50 mV/s, 100 mV/s, and 250 mV/s), potential reported against $Me_{10}Fc/Me_{10}Fc^+$ ($Me_{10}Fc$ was added to the solution).

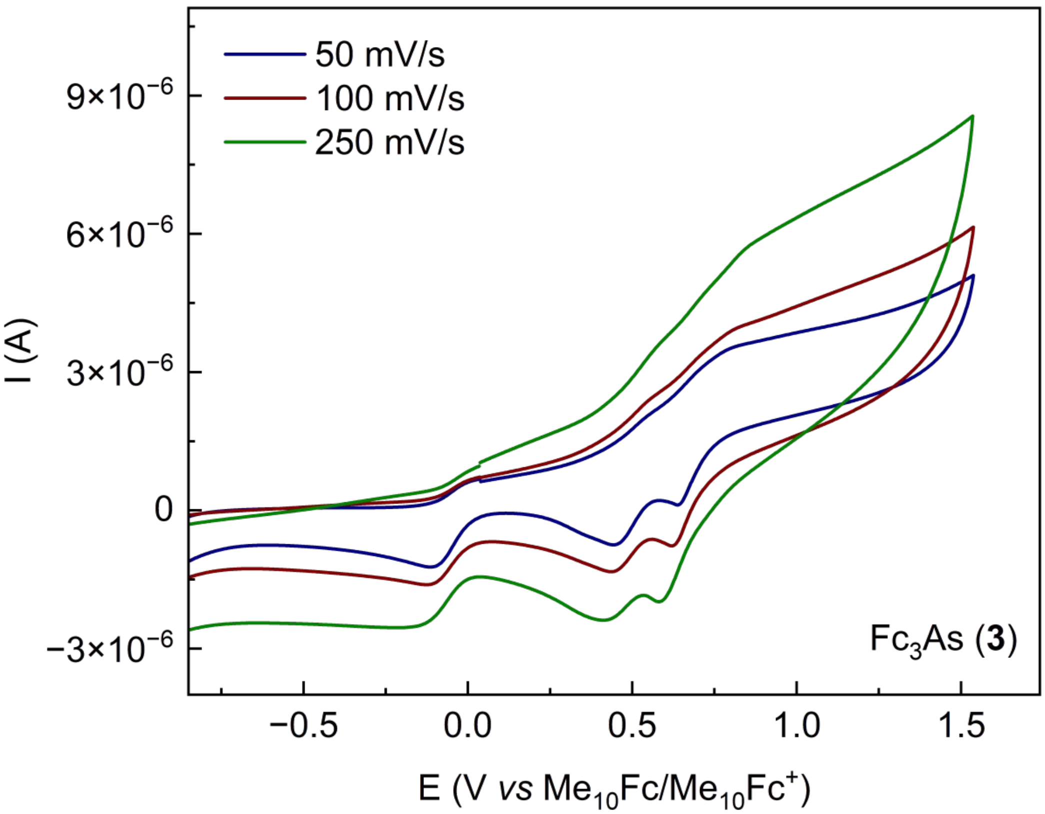

Figure S31. Cyclic voltammograms of **3** (1 mM) in **dichloromethane** at −80 °C (**193 K**) [*n*-Bu_4N][$\mathbf{PF_6}$] (0.1 M) as supporting electrolyte and different scan rates (50 mV/s, 100 mV/s, and 250 mV/s), potential reported against $Me_{10}Fc/Me_{10}Fc^+$ ($Me_{10}Fc$ was added to the solution).

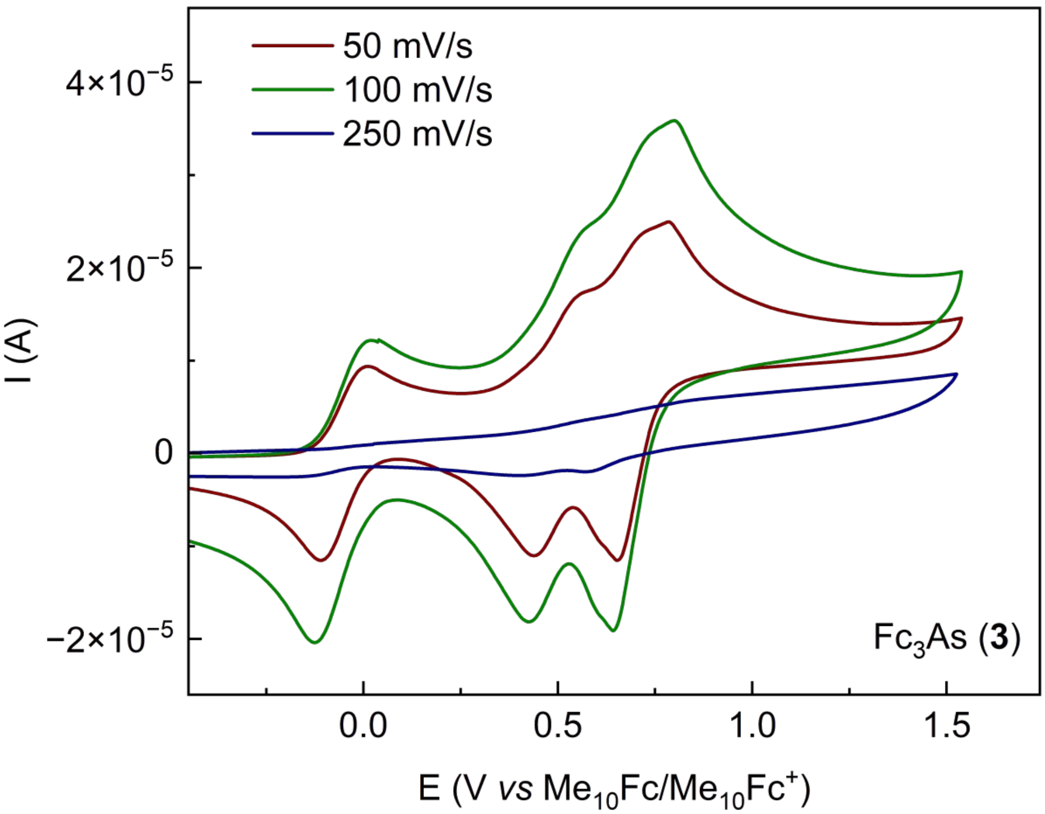

Figure S32. Cyclic voltammograms of **3** (1 mM) in **dichloromethane** at room temperature (**298 K**), [*n*-Bu_4N][$\mathbf{PF_6}$] (0.1 M) as supporting electrolyte and different scan rates (50 mV/s, 100 mV/s, and 250 mV/s), potential reported against $Me_{10}Fc/Me_{10}Fc^+$ ($Me_{10}Fc$ was added to the solution).

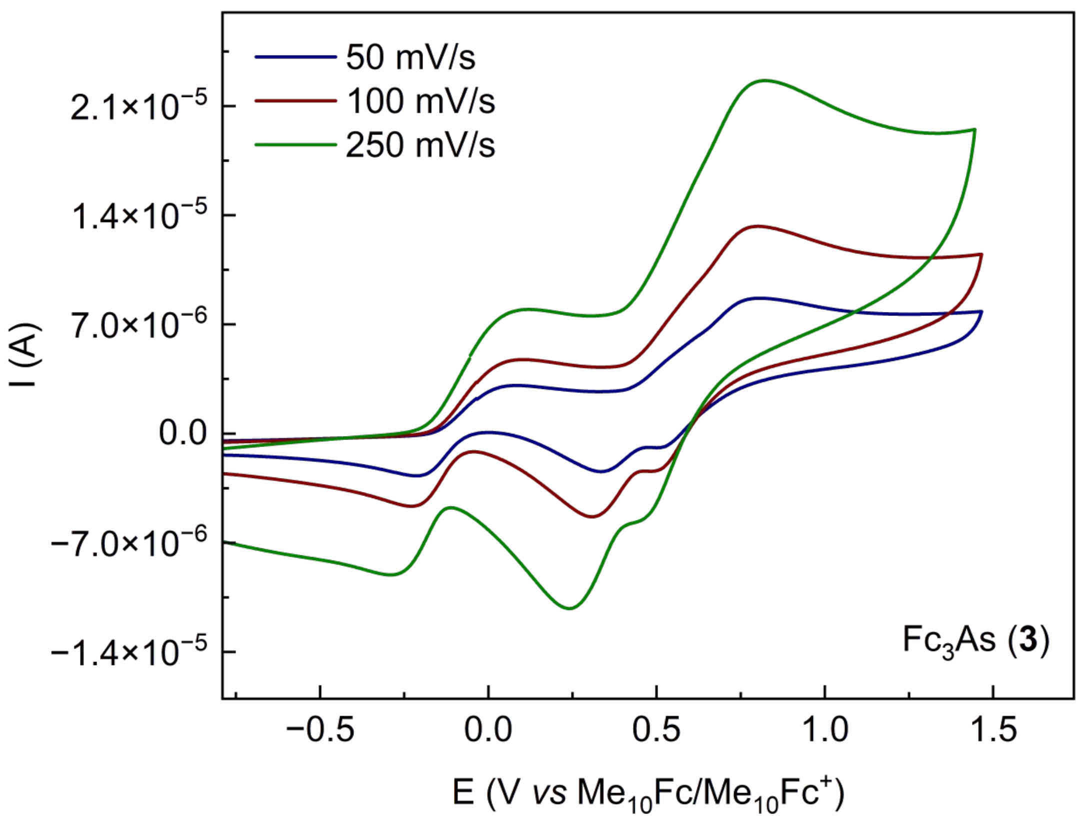

Figure S33. Cyclic voltammograms of **3** (1 mM) in **dichloromethane** at −80 °C (**193 K**) [*n*-Bu_4N][**SbF_6**] (0.1 M) as supporting electrolyte and different scan rates (50 mV/s, 100 mV/s, and 250 mV/s), potential reported against $Me_{10}Fc/Me_{10}Fc^+$ ($Me_{10}Fc$ was added to the solution).

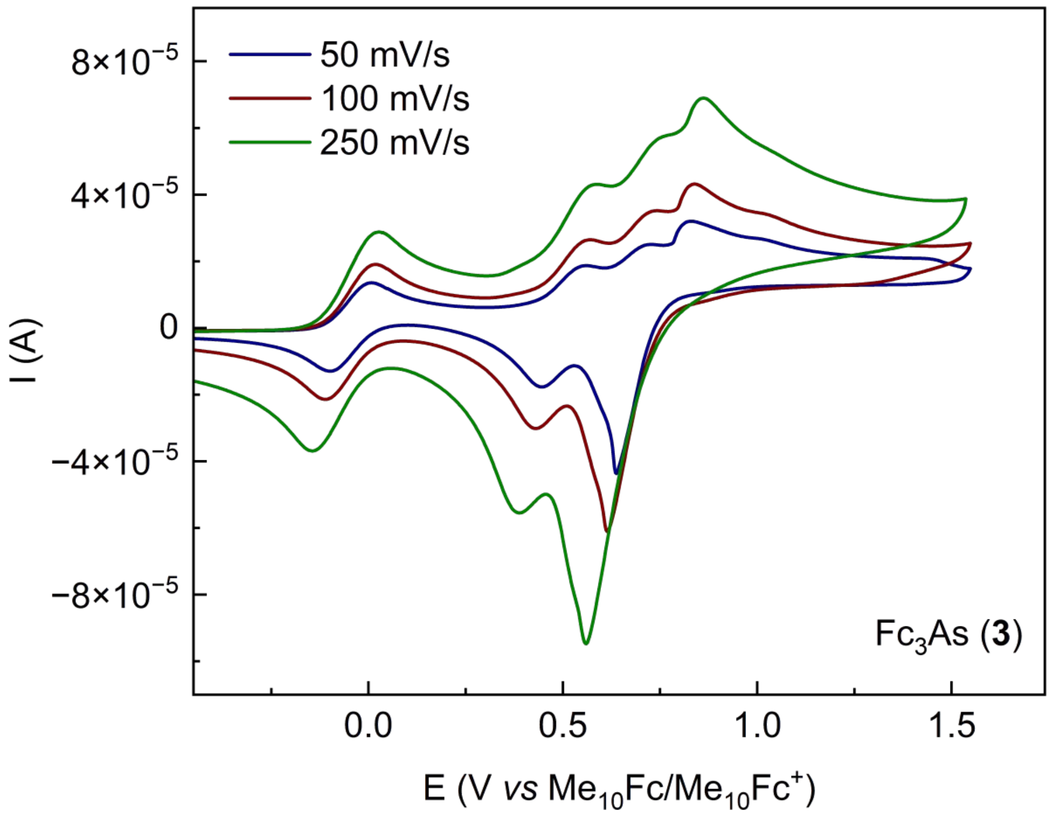

Figure S34. Cyclic voltammograms of **3** (1 mM) in **dichloromethane** at room temperature (**298 K**), [*n*-Bu_4N][**SbF_6**] (0.1 M) as supporting electrolyte and different scan rates (50 mV/s, 100 mV/s, and 250 mV/s), potential reported against $Me_{10}Fc/Me_{10}Fc^+$ ($Me_{10}Fc$ was added to the solution).

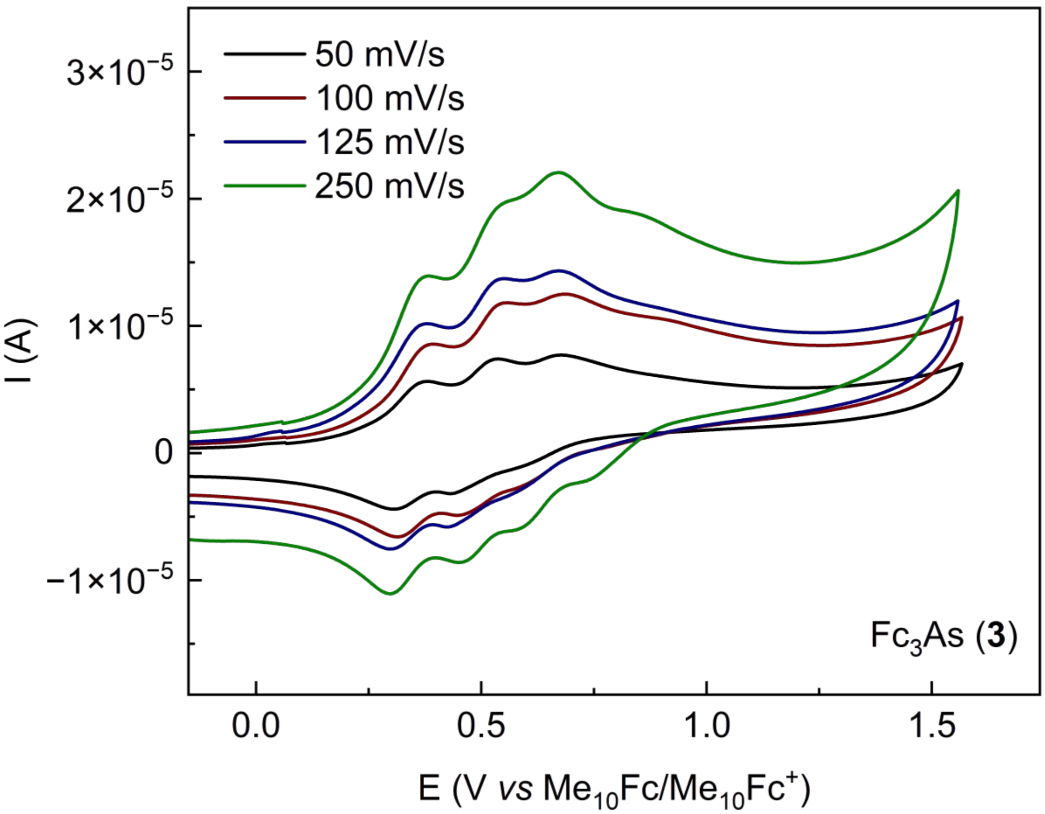

Figure S35. Cyclic voltammograms of **3** (0.25 mM) in **MeCN:CH_2Cl_2** (10:1, V:V) at room temperature (**298 K**), [*n*-Bu_4N][**PF_6**] (25 mM) as supporting electrolyte and different scan rates (50 mV/s, 100 mV/s, and 250 mV/s), potential reported against $Me_{10}Fc/Me_{10}Fc^+$.

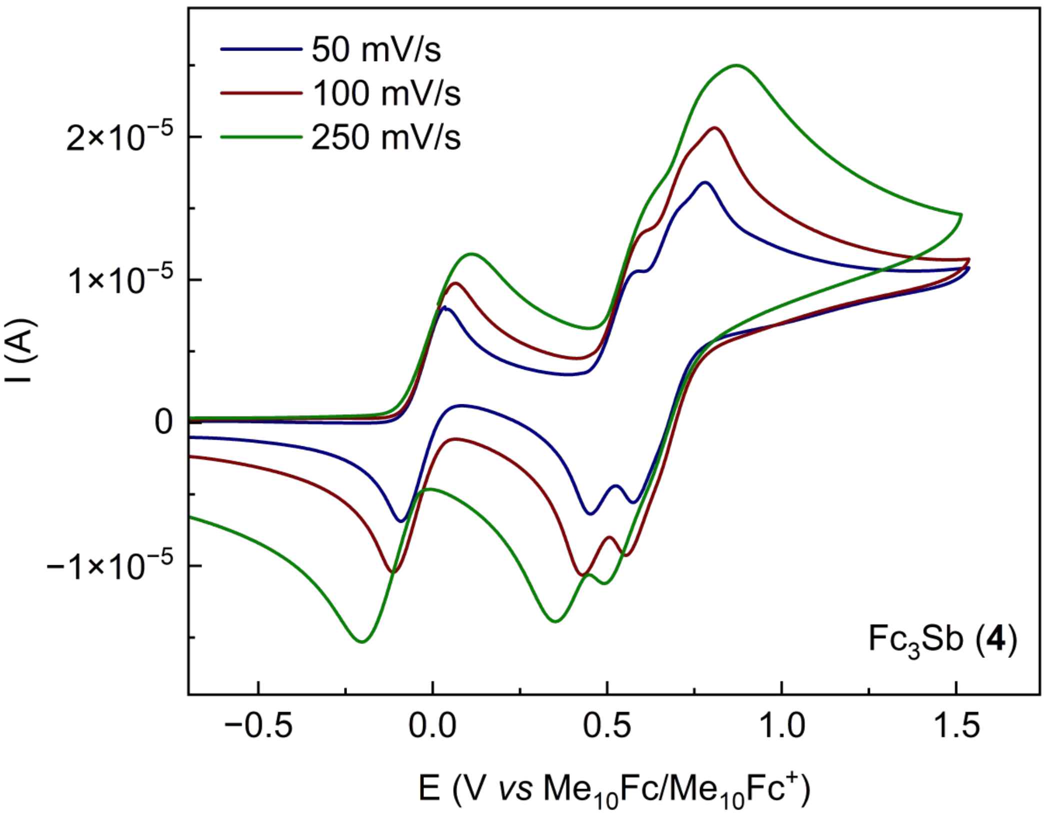

Figure S36. Cyclic voltammograms of **4** (1 mM) in **dichloromethane** at −80 °C (**193 K**) [*n*-Bu_4N][**BF_4**] (0.1 M) as supporting electrolyte and different scan rates (50 mV/s, 100 mV/s, and 250 mV/s), potential reported against $Me_{10}Fc/Me_{10}Fc^+$ ($Me_{10}Fc$ was added to the solution).

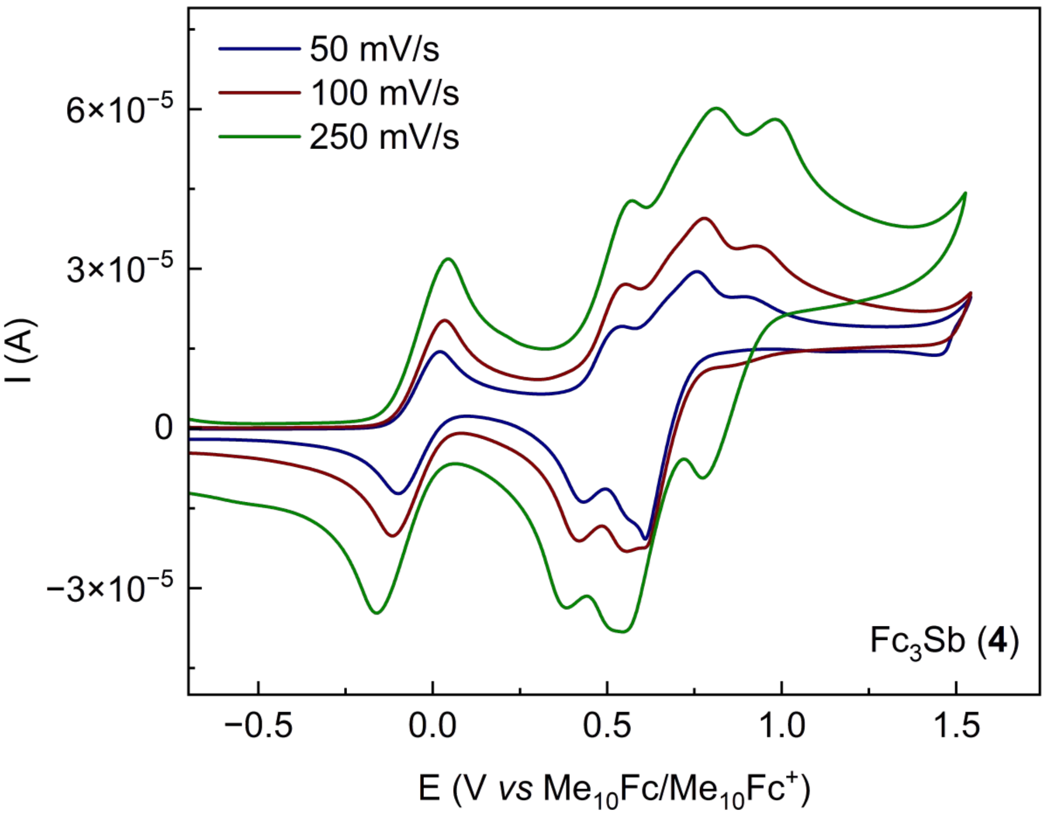

Figure S37. Cyclic voltammograms of **4** (1 mM) in **dichloromethane** at room temperature (**298 K**), [*n*-Bu_4N][$\mathbf{BF_4}$] (0.1 M) as supporting electrolyte and different scan rates (50 mV/s, 100 mV/s, and 250 mV/s), potential reported against $Me_{10}Fc/Me_{10}Fc^+$ ($Me_{10}Fc$ was added to the solution).

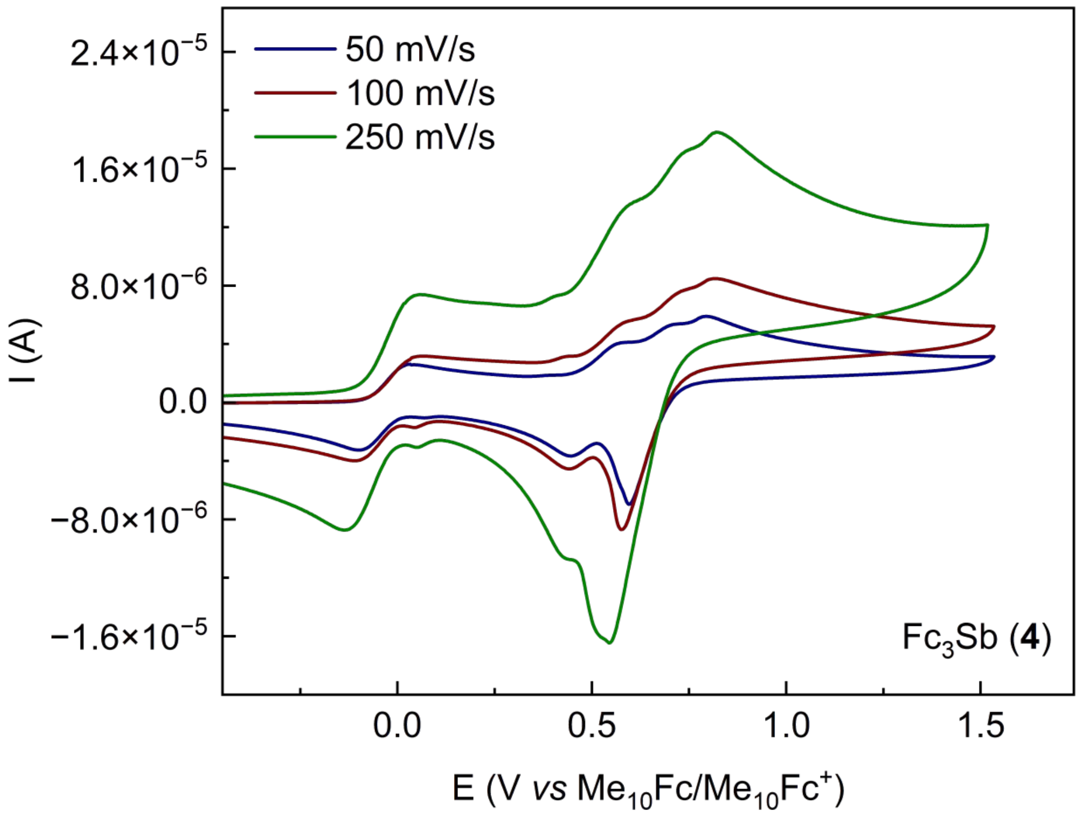

Figure S38. Cyclic voltammograms of **4** (1 mM) in **dichloromethane** at −80 °C (**193 K**) [*n*-Bu_4N][$\mathbf{PF_6}$] (0.1 M) as supporting electrolyte and different scan rates (50 mV/s, 100 mV/s, and 250 mV/s), potential reported against $Me_{10}Fc/Me_{10}Fc^+$ ($Me_{10}Fc$ was added to the solution).

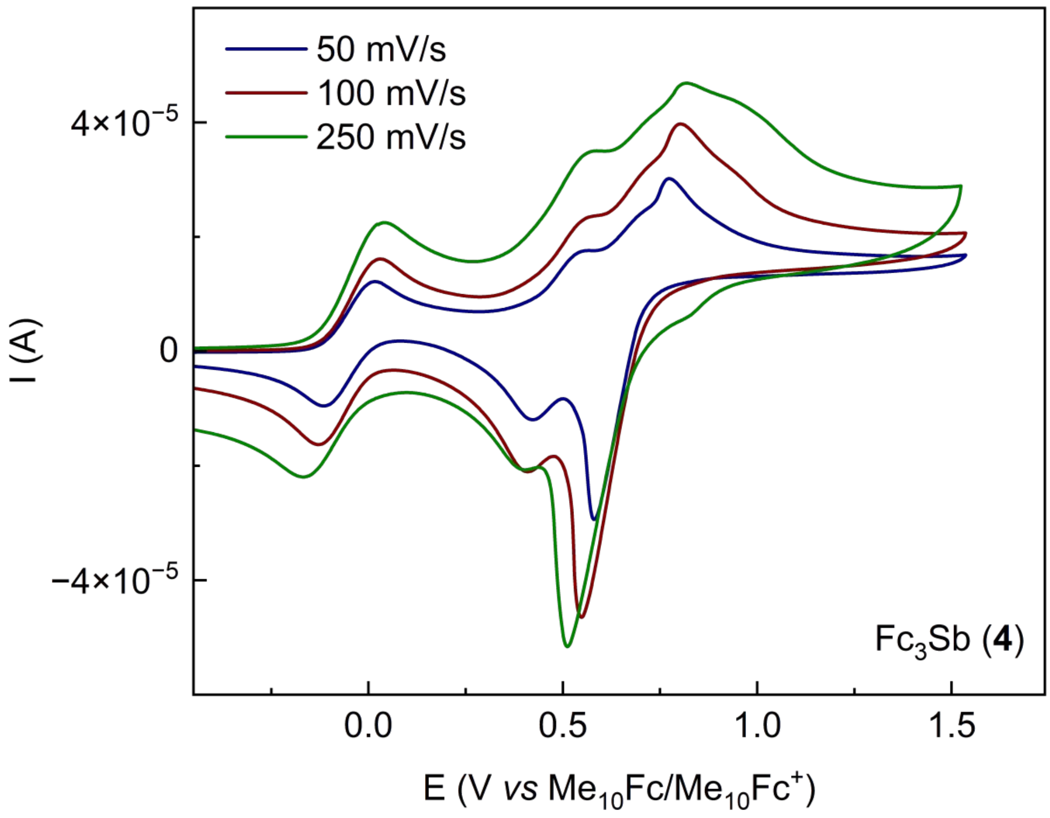

Figure S39. Cyclic voltammograms of **4** (1 mM) in **dichloromethane** at room temperature (**298 K**), [*n*-Bu$_4$N][**PF$_6$**] (0.1 M) as supporting electrolyte and different scan rates (50 mV/s, 100 mV/s, and 250 mV/s), potential reported against Me$_{10}$Fc/Me$_{10}$Fc$^+$ (Me$_{10}$Fc was added to the solution).

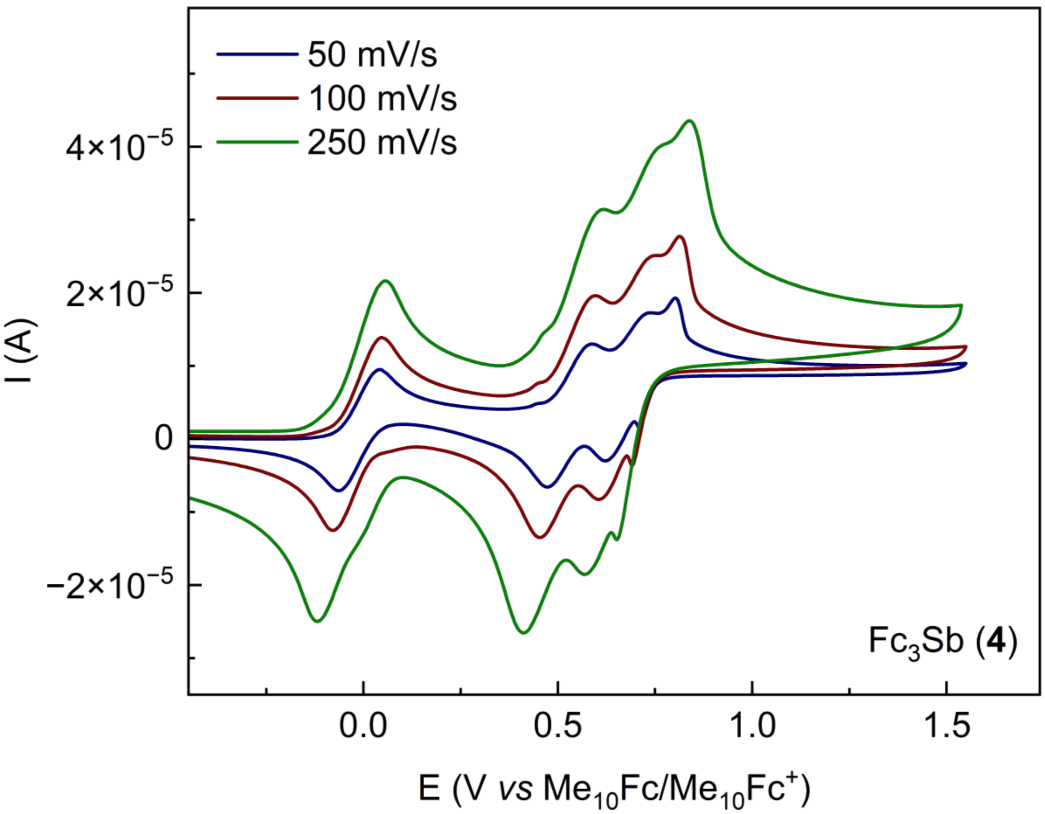

Figure S40. Cyclic voltammograms of **4** (1 mM) in **dichloromethane** at −80 °C (**193 K**) [*n*-Bu_4N][**SbF_6**] (0.1 M) as supporting electrolyte and different scan rates (50 mV/s, 100 mV/s, and 250 mV/s), potential reported against $Me_{10}Fc/Me_{10}Fc^+$ ($Me_{10}Fc$ was added to the solution).

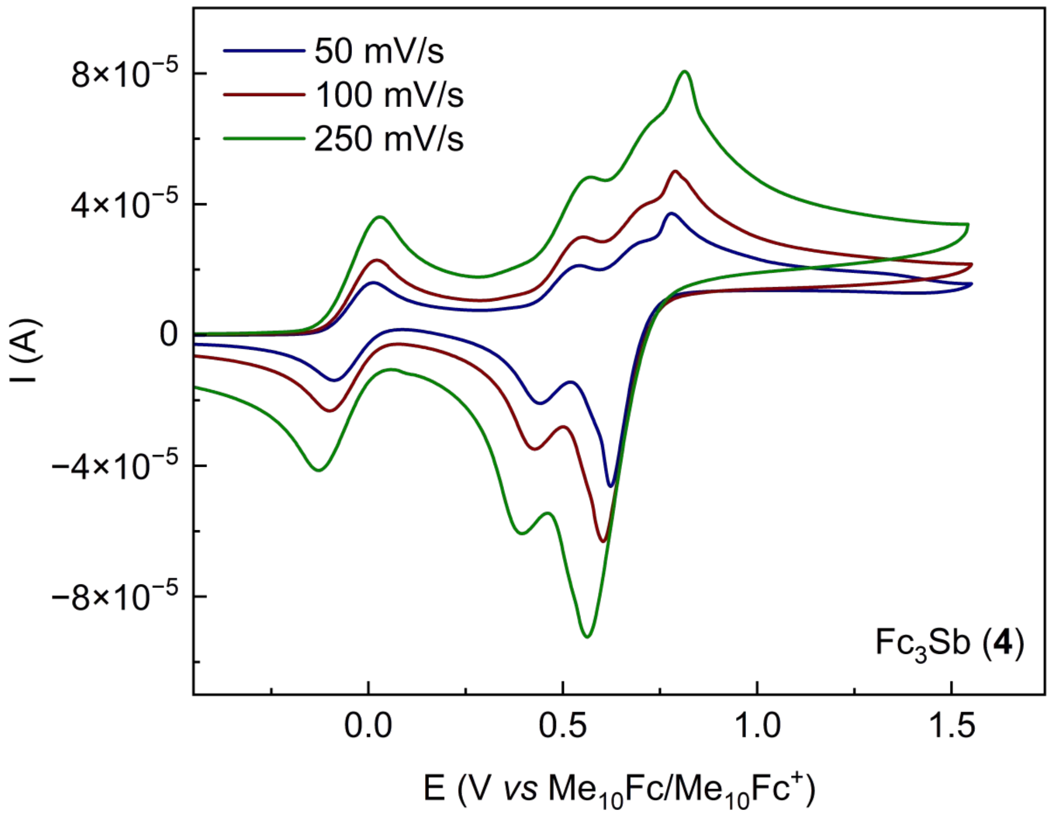

Figure S41. Cyclic voltammograms of **4** (1 mM) in **dichloromethane** at room temperature (**298 K**), [*n*-Bu_4N][**SbF_6**] (0.1 M) as supporting electrolyte and different scan rates (50 mV/s, 100 mV/s, and 250 mV/s), potential reported against $Me_{10}Fc/Me_{10}Fc^+$ ($Me_{10}Fc$ was added to the solution).

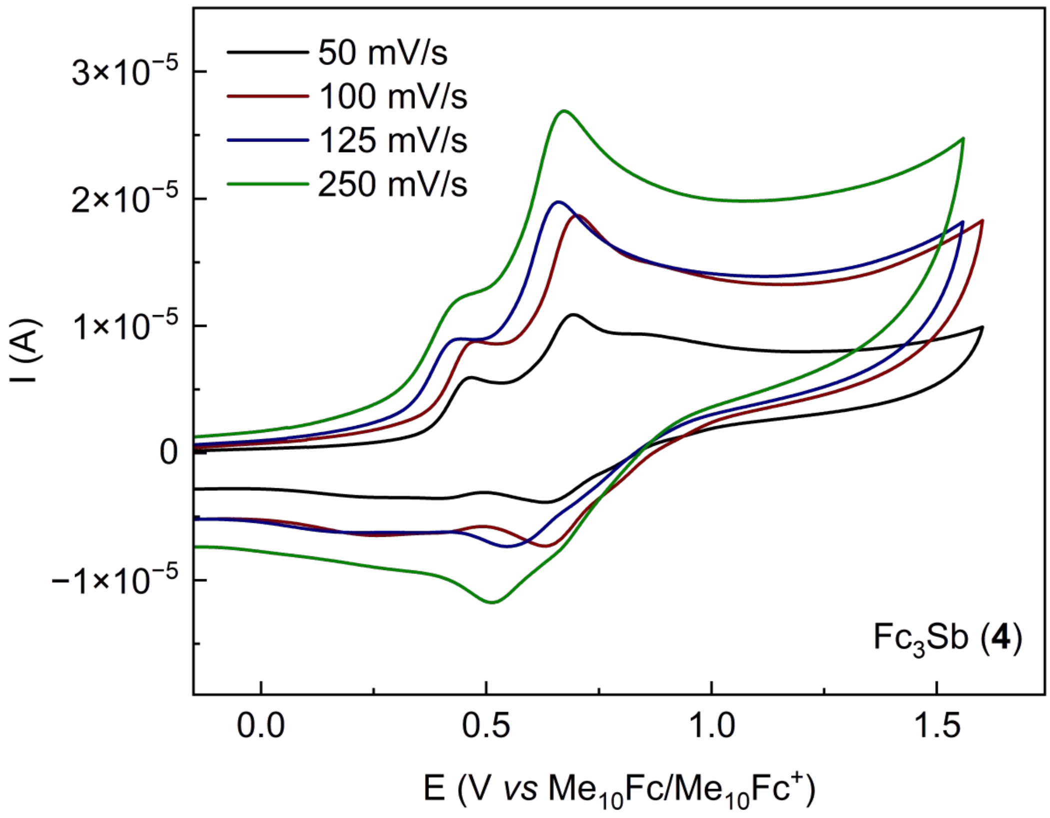

Figure S42. Cyclic voltammograms of **4** (0.25 mM) in **MeCN:CH_2Cl_2** (10:1, V:V) at room temperature (**298 K**), [*n*-Bu_4N][**PF_6**] (25 mM) as supporting electrolyte and different scan rates (50 mV/s, 100 mV/s, and 250 mV/s), potential reported against $Me_{10}Fc/Me_{10}Fc^+$.

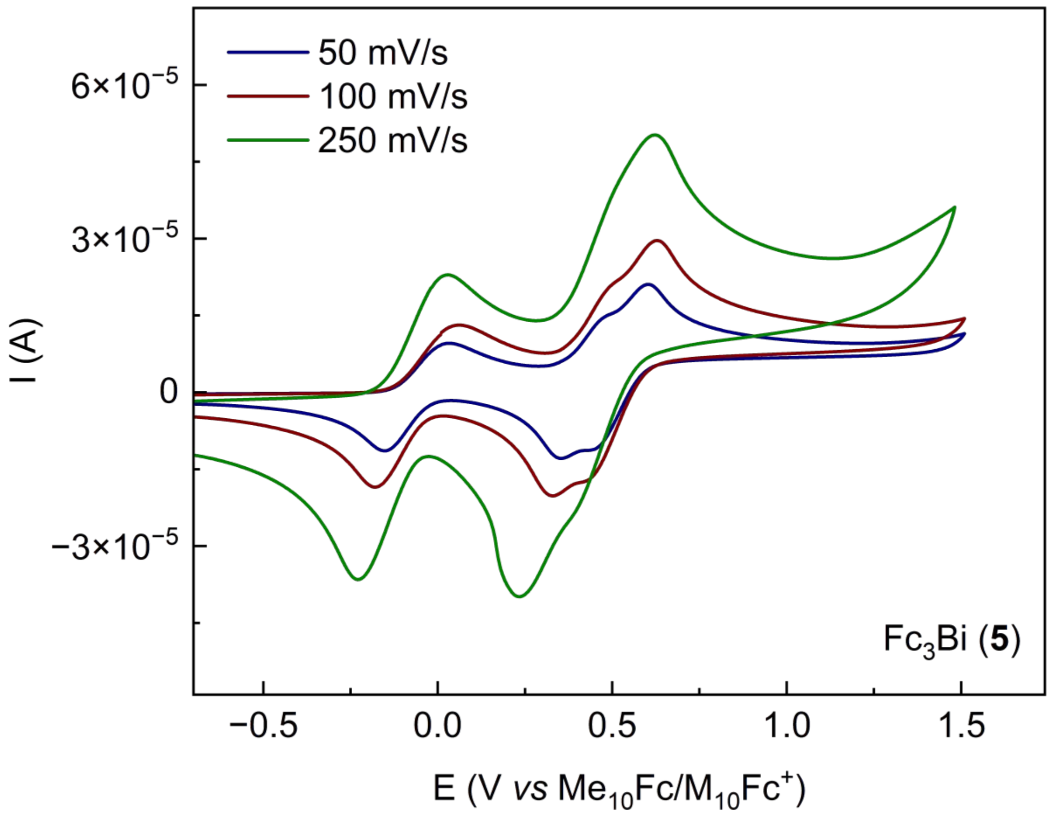

Figure S43. Cyclic voltammograms of **5** (1 mM) in **dichloromethane** at −80 °C (**193 K**) [*n*-Bu_4N][$\mathbf{BF_4}$] (0.1 M) as supporting electrolyte and different scan rates (50 mV/s, 100 mV/s, and 250 mV/s), potential reported against $Me_{10}Fc/Me_{10}Fc^+$ ($Me_{10}Fc$ was added to the solution).

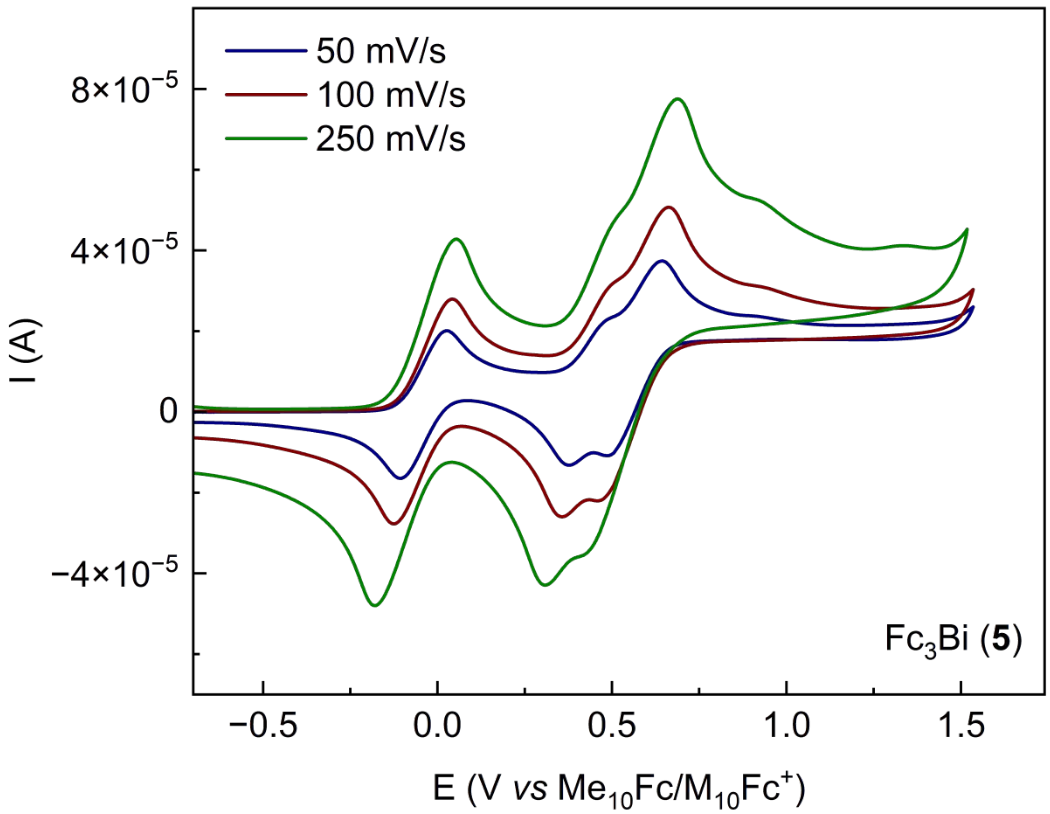

Figure S44. Cyclic voltammograms of **5** (1 mM) in **dichloromethane** at room temperature (**298 K**), [*n*-Bu_4N][$\mathbf{BF_4}$] (0.1 M) as supporting electrolyte and different scan rates (50 mV/s, 100 mV/s, and 250 mV/s), potential reported against $Me_{10}Fc/Me_{10}Fc^+$ ($Me_{10}Fc$ was added to the solution).

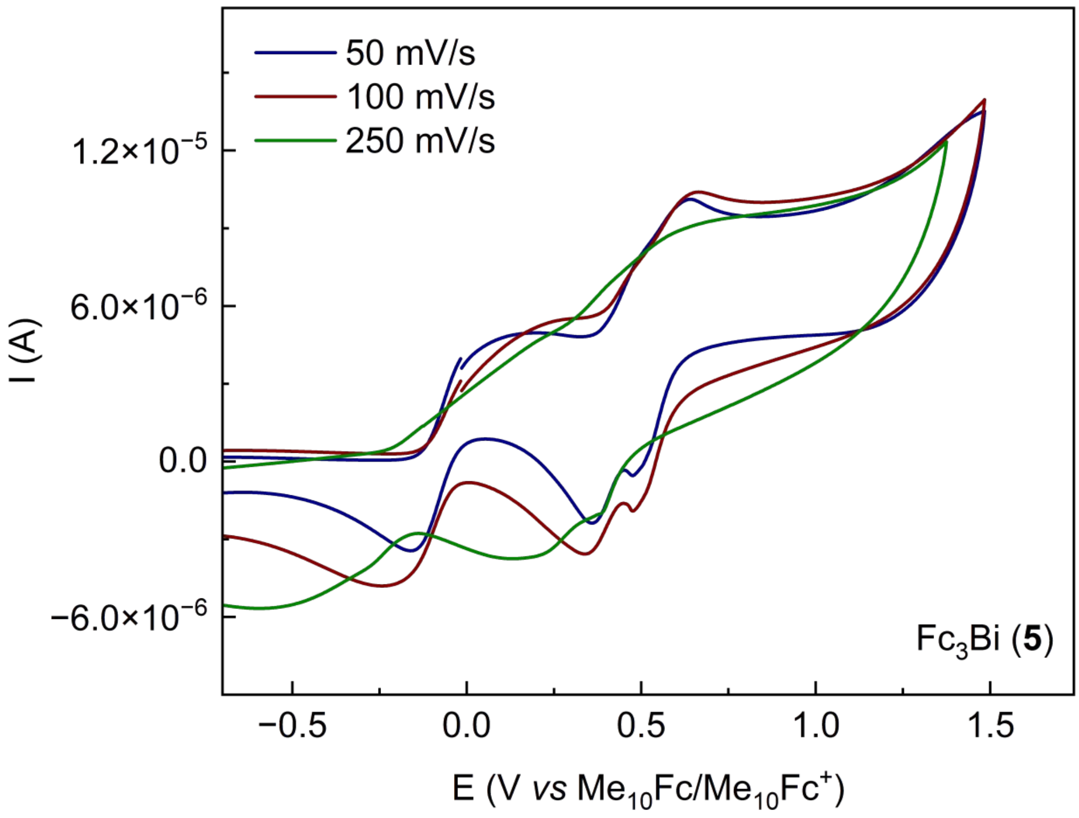

Figure S45. Cyclic voltammograms of **5** (1 mM) in **dichloromethane** at −80 °C (**193 K**) [*n*-Bu_4N][$\mathbf{PF_6}$] (0.1 M) as supporting electrolyte and different scan rates (50 mV/s, 100 mV/s, and 250 mV/s), potential reported against $Me_{10}Fc/Me_{10}Fc^+$ ($Me_{10}Fc$ was added to the solution).

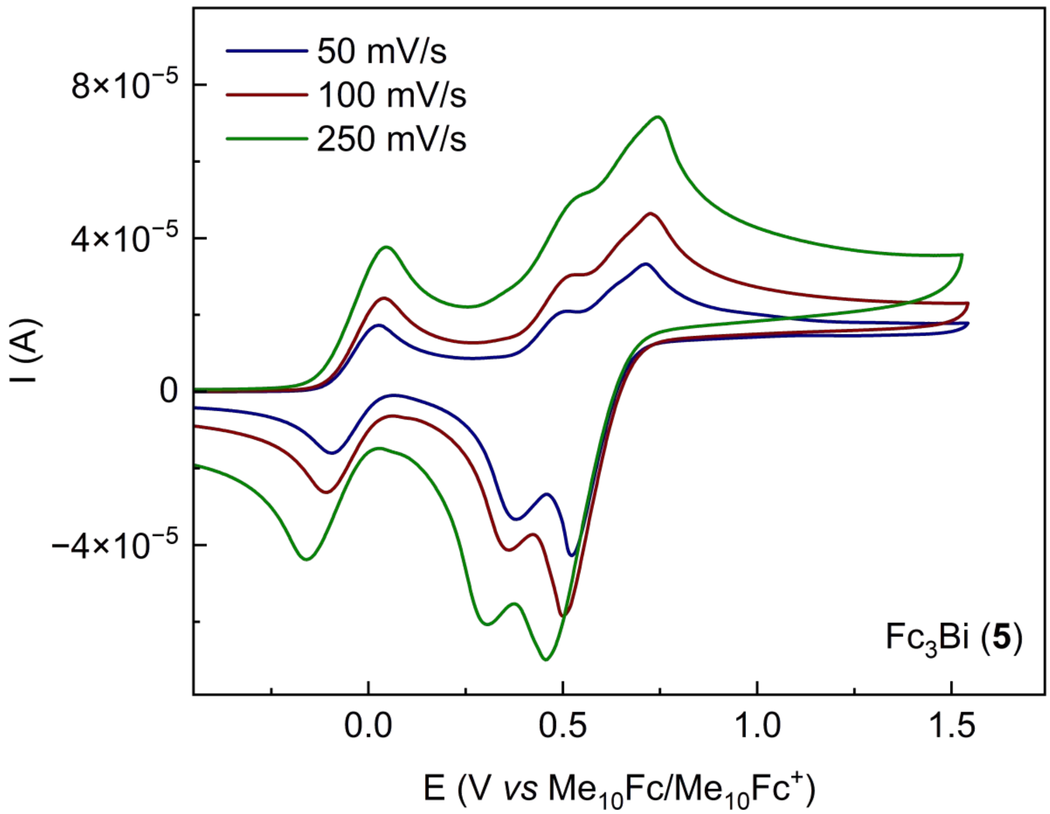

Figure S46. Cyclic voltammograms of **5** (1 mM) in **dichloromethane** at room temperature (**298 K**), [*n*-Bu_4N][$\mathbf{PF_6}$] (0.1 M) as supporting electrolyte and different scan rates (50 mV/s, 100 mV/s, and 250 mV/s), potential reported against $Me_{10}Fc/Me_{10}Fc^+$ ($Me_{10}Fc$ was added to the solution).

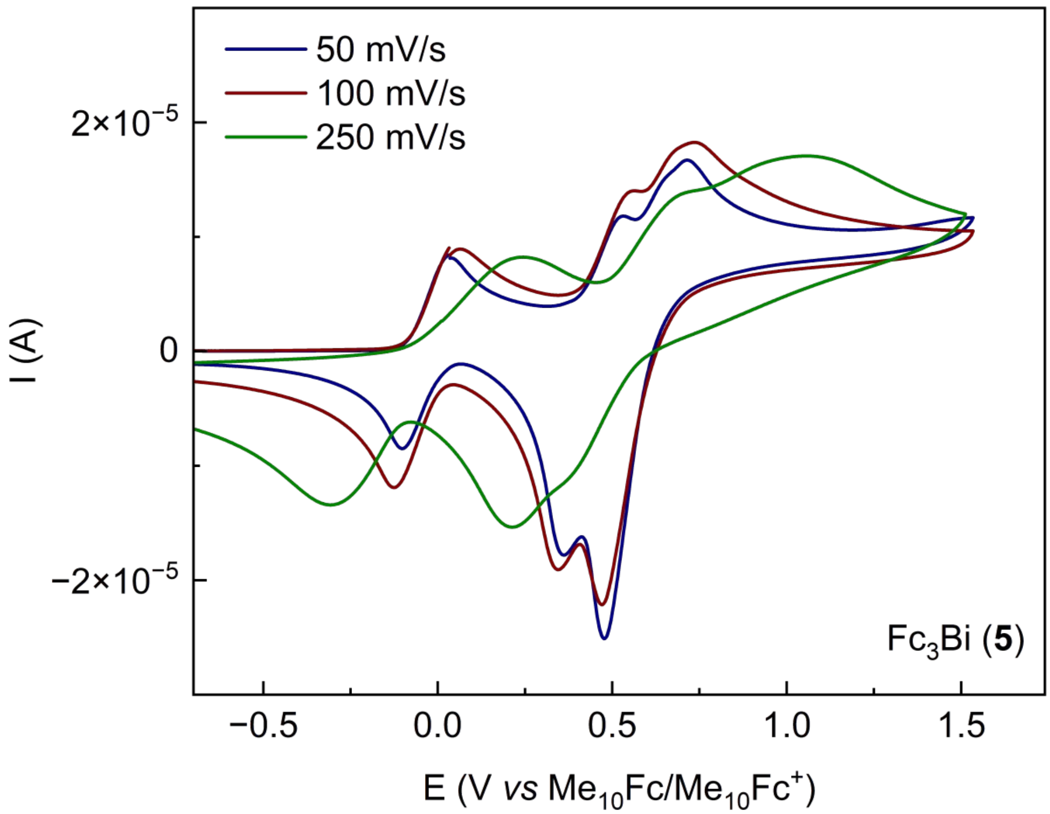

Figure S47. Cyclic voltammograms of **5** (1 mM) in **dichloromethane** at −80 °C (**193 K**) [*n*-Bu_4N][**SbF_6**] (0.1 M) as supporting electrolyte and different scan rates (50 mV/s, 100 mV/s, and 250 mV/s), potential reported against $Me_{10}Fc/Me_{10}Fc^+$ ($Me_{10}Fc$ was added to the solution).

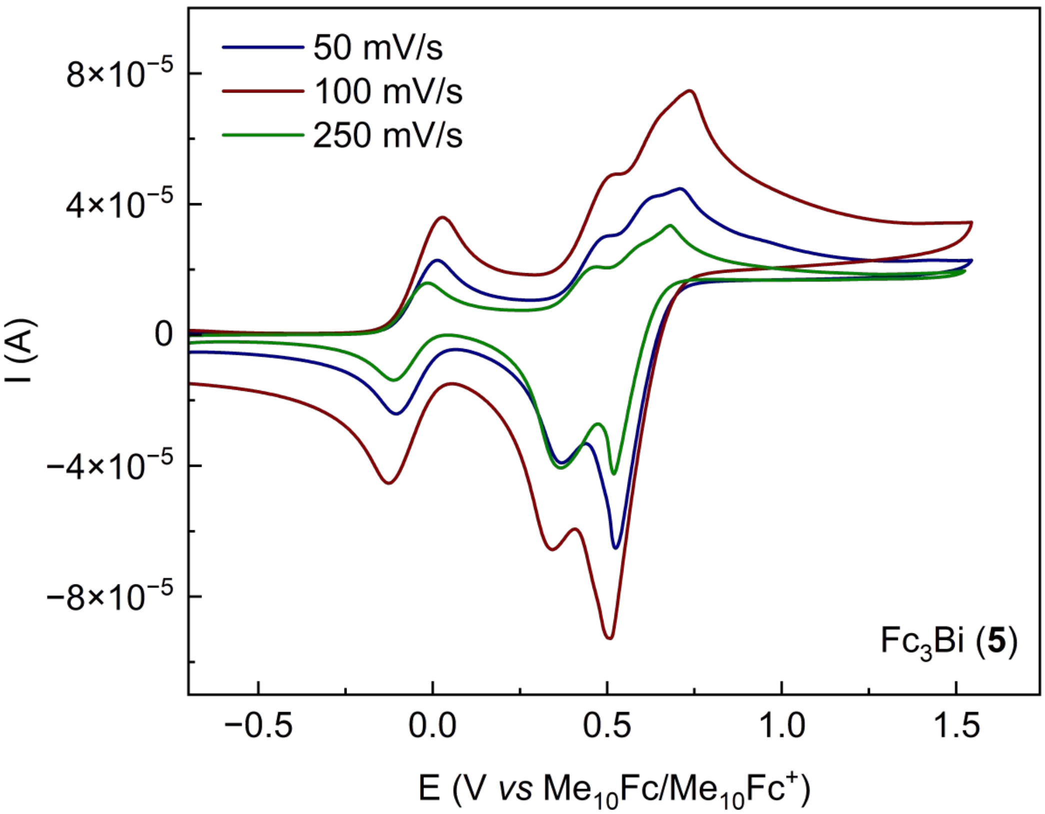

Figure S48. Cyclic voltammograms of **5** (1 mM) in **dichloromethane** at room temperature (**298 K**) [*n*-Bu_4N][**SbF_6**] (0.1 M) as supporting electrolyte and different scan rates (50 mV/s, 100 mV/s, and 250 mV/s), potential reported against $Me_{10}Fc/Me_{10}Fc^+$ ($Me_{10}Fc$ was added to the solution).

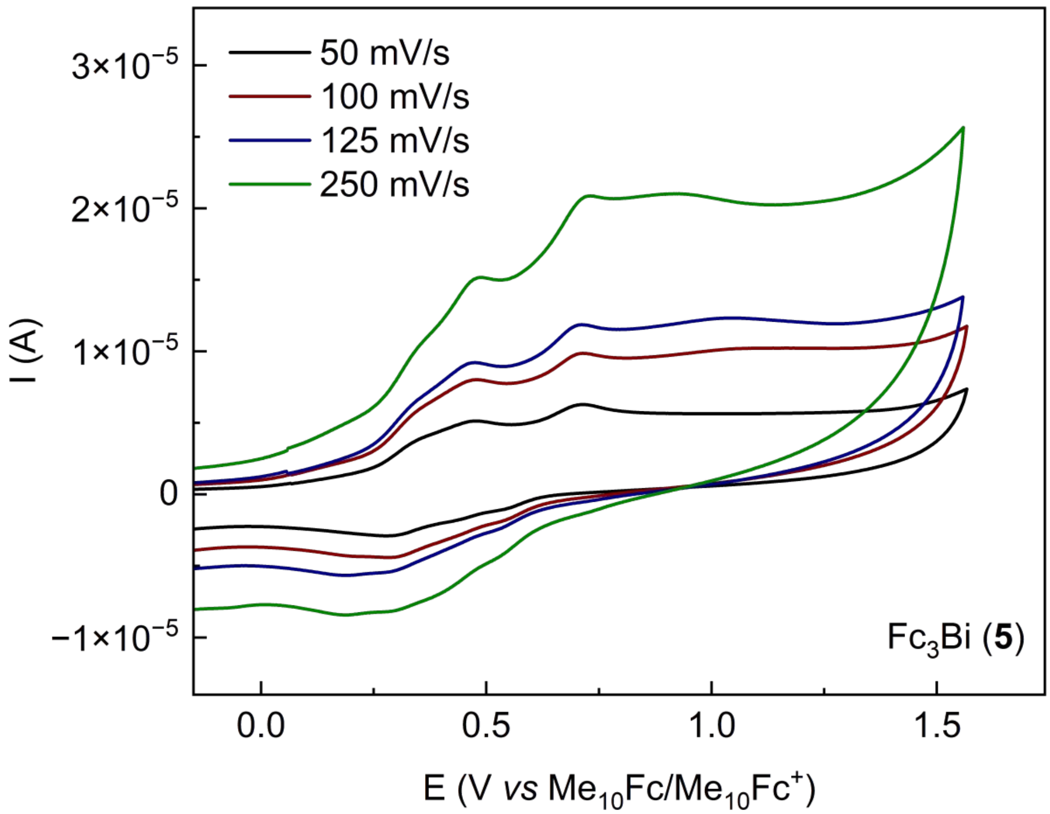

Figure S49. Cyclic voltammograms of **5** (0.25 mM) in **MeCN:CH_2Cl_2** (10:1, V:V) at room temperature (**298 K**) [n-Bu_4N][**PF_6**] (25 mM) as supporting electrolyte and different scan rates (50 mV/s, 100 mV/s, and 250 mV/s), potential reported against $Me_{10}Fc/Me_{10}Fc^+$.

Spectroelectrochemistry

$$H_{ab} = \frac{2.06 \times 10^{-2}\sqrt{\varepsilon_{max}\nu_{max}\Delta\nu_{1/2}}}{r\sqrt{2}}$$

$$E_{op} = \lambda + \Delta G^{\circ}$$

$$\Delta\nu_{1/2_{calc}} = [2310(E_{op} - \Delta E)]^{1/2}$$

$$\alpha = \frac{H_{ab}}{\nu_{max}}$$

$\boldsymbol{\alpha^2}$ – Delocalization parameter.

$\boldsymbol{\Delta\nu_{1/2}}$ – Peak width at half height.

$\boldsymbol{r}$ – Inter component distance (r_{ab}).

$\boldsymbol{E_{op}}$ – Energy of the transition

(E of absorption maximum of the IV band).

ΔE – Energy difference between oxidation maxima

(same as ΔE_1 in the manuscript);

$\boldsymbol{H_{ab}}$ – Coupling constant.

λ – reorganisation energy (experimentally determined from IVCT band, ν_{max})

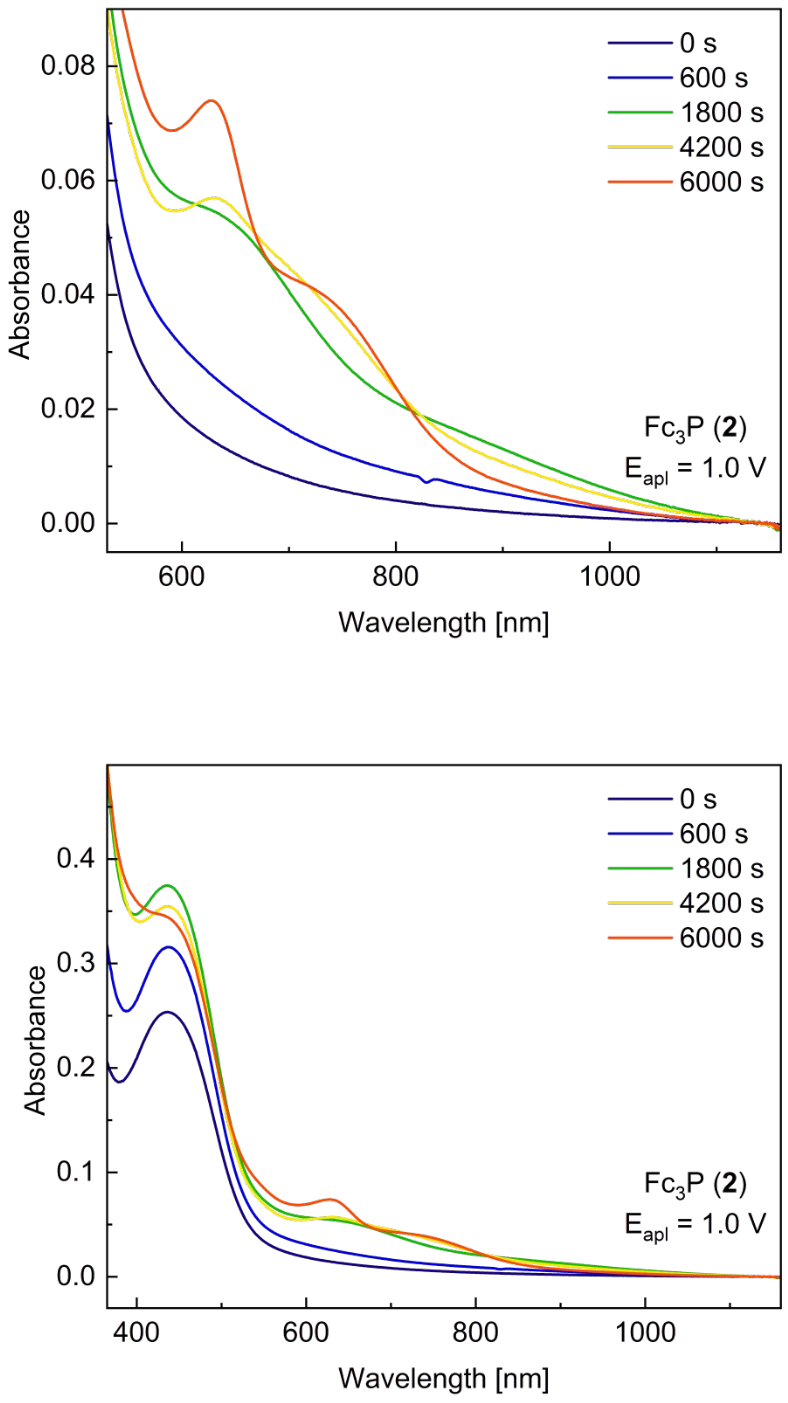

Figure S50. UV-vis absorption spectra of Fc_3P (**2**) at different times during bulk oxidation (applied voltage of 1.0 V). Upper image shows a zoom in into LMCT band.

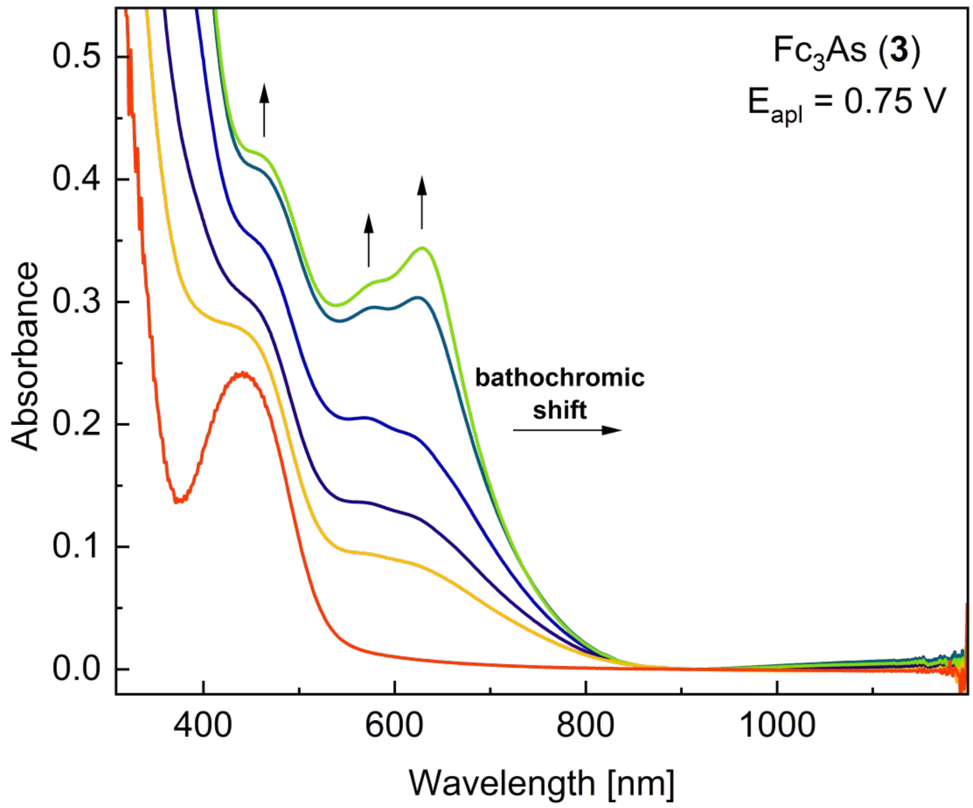

Figure S51. UV-vis absorption spectra of Fc_3As (**3**) at different times during bulk oxidation (0 s, 600 s, 1200 s, 2400 s, 4800 s and 6000 s; applied voltage of 0.75 V for the first oxidation).

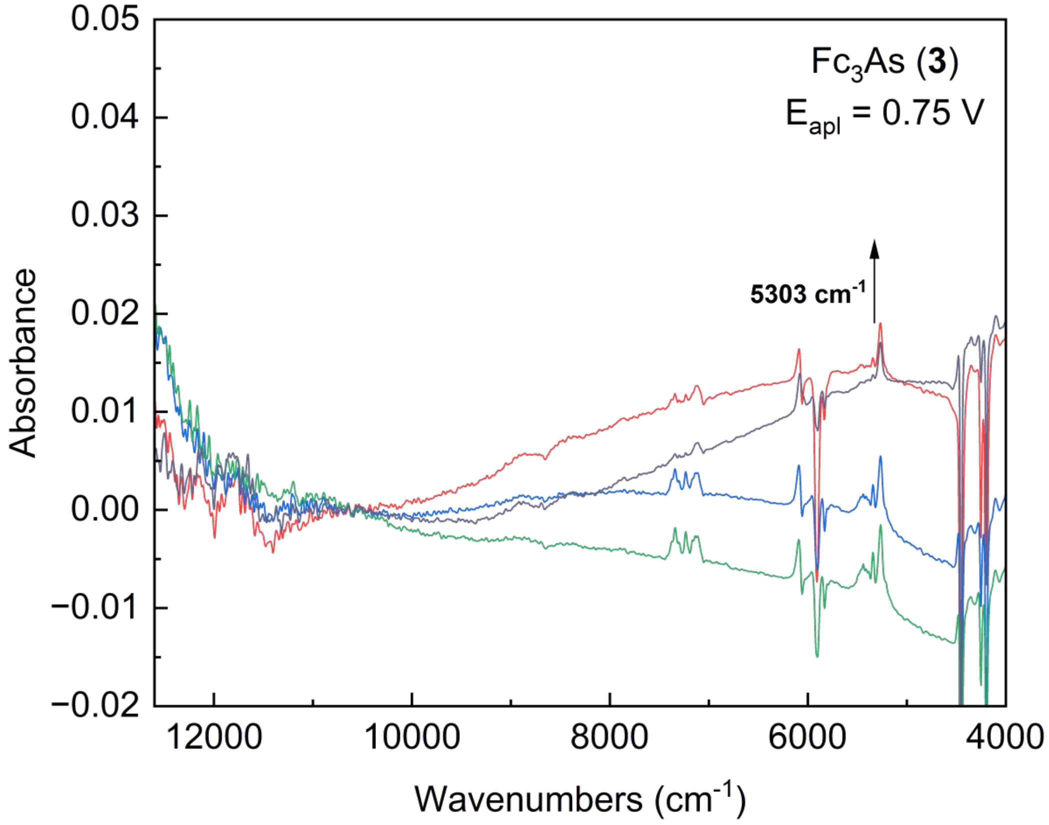

Figure S52. NIR absorption spectra of Fc_3As (**3**) at different times during bulk oxidation (600 s, 1200 s, 2400 s, 4800 s and 6000 s; applied voltage of 0.75 V for the first oxidation); negative bands due to CH_2Cl_2 overtones.

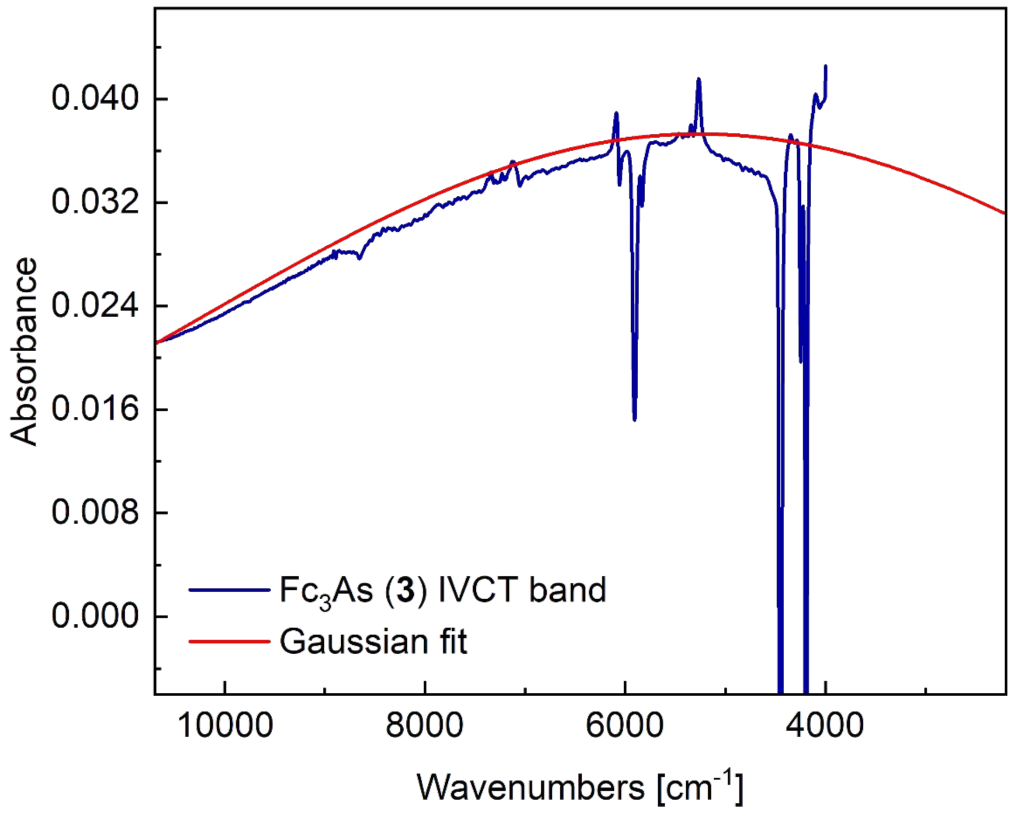

Figure S53. IVCT band of Fc_3As (**3**) t = 6000 s during bulk oxidation (applied voltage of 0.75 V) and the Gaussian fit. Estimated ε_{IVCT} = 112 L*mol^{-1} *cm^{-1} (negative bands due to CH_2Cl_2 overtones).

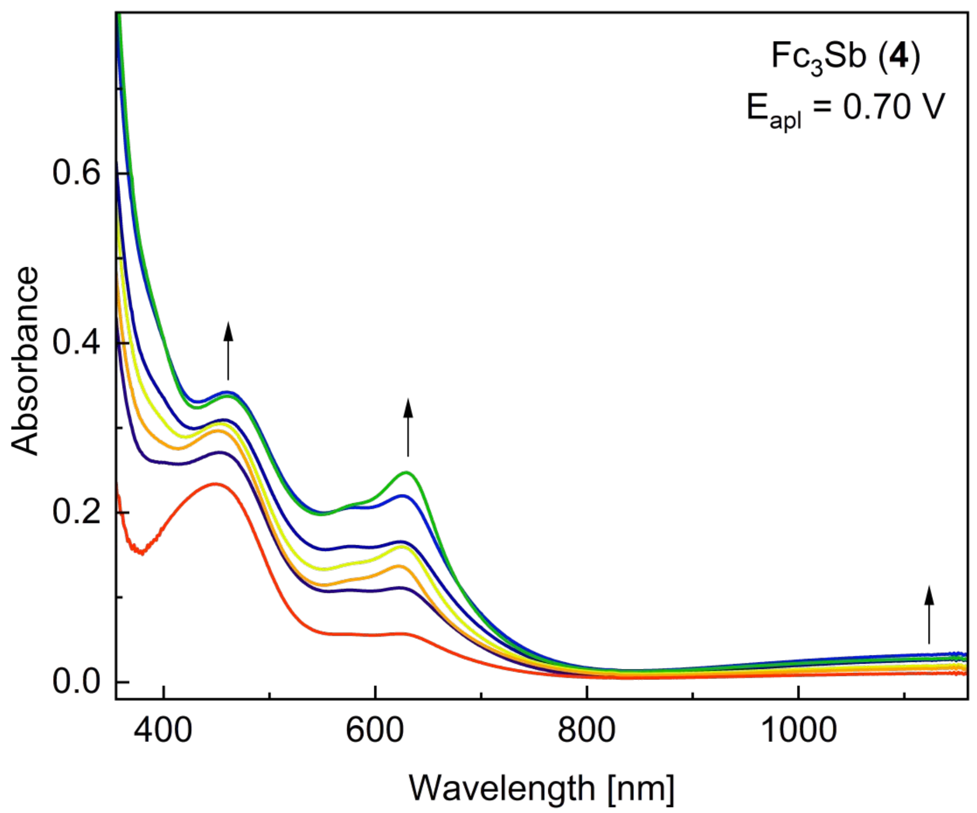

Figure S54. UV-vis absorption spectra of Fc_3Sb (**4**) at different times during bulk oxidation (600 s, 1200 s, 1800 s, 2400 s, 3000 s, 3600 s and 4200 s; applied voltage of 0.70 V for the first oxidation).

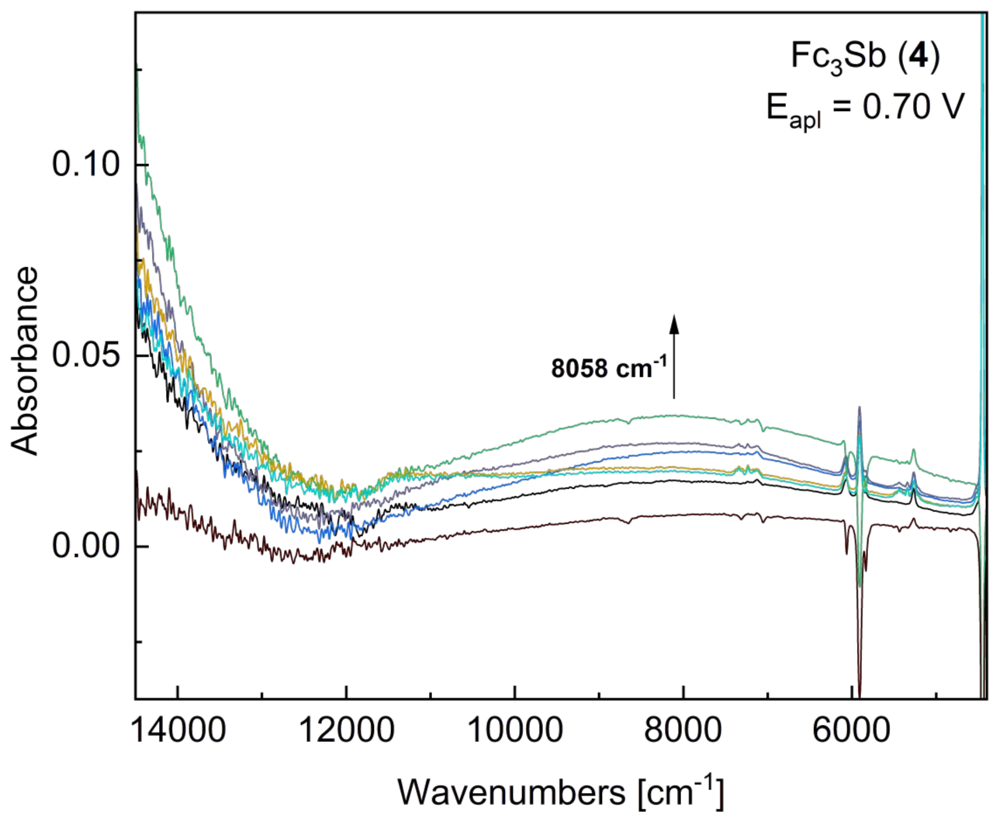

Figure S55. NIR absorption spectra of Fc_3Sb (**4**) at different times during bulk oxidation (600 s, 1200 s, 1800 s, 2400 s, 3000 s, 3600 s and 4200 s; applied voltage of 0.70 V for the first oxidation); negative bands due to CH_2Cl_2 overtones.

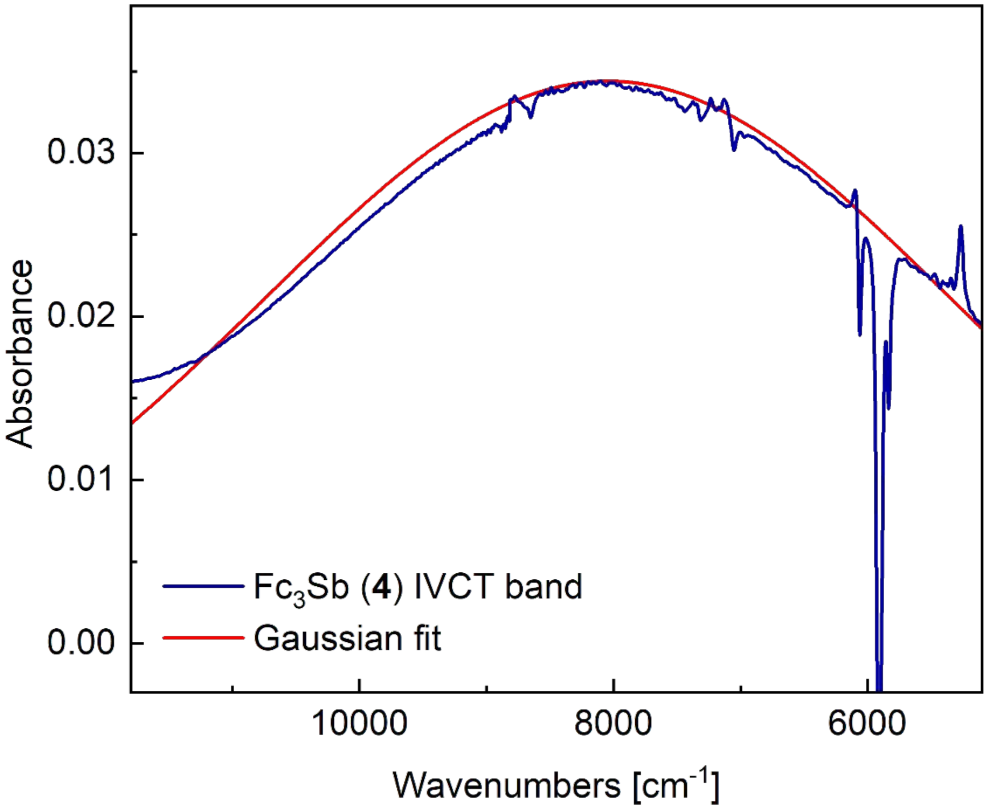

Figure S56. IVCT band of Fc_3Sb (**4**) t = 4200 s during bulk oxidation (applied voltage of 0.70 V) and the Gaussian fit. Estimated ε_{IVCT} = 82 $L*mol^{-1}*cm^{-1}$ (negative bands due to CH_2Cl_2 overtones).

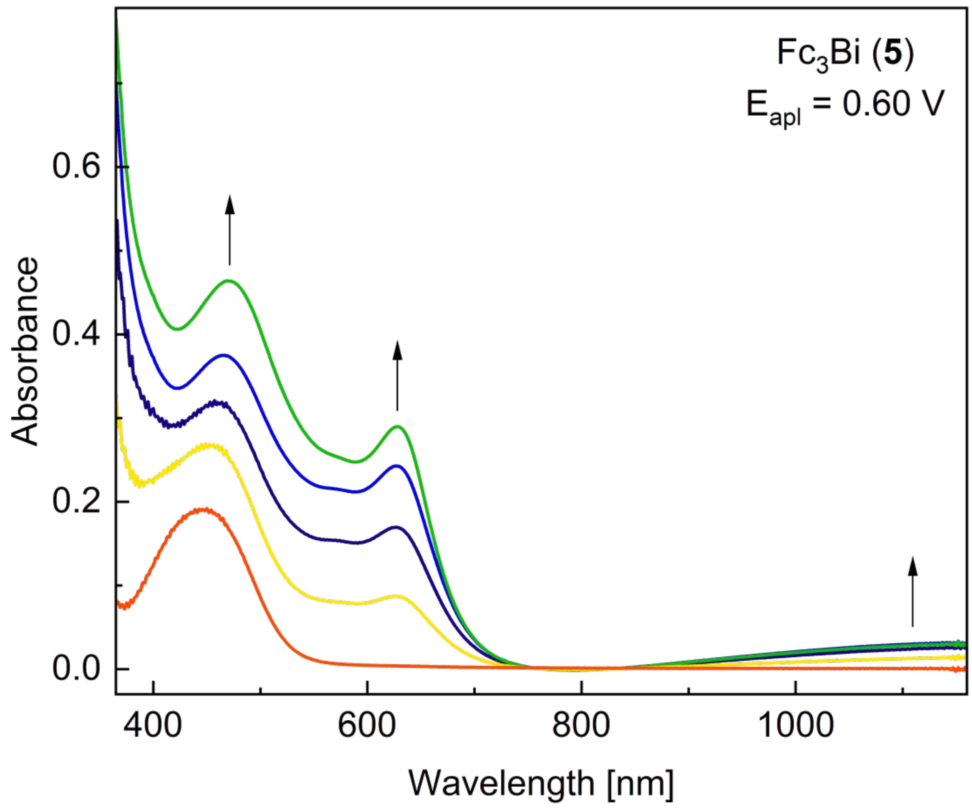

Figure S57. UV-vis absorption spectra of Fc_3Bi (**5**) at different times during bulk oxidation (0 s, 600 s, 1200 s and 2400 s; applied voltage of 0.60 V for the first oxidation).

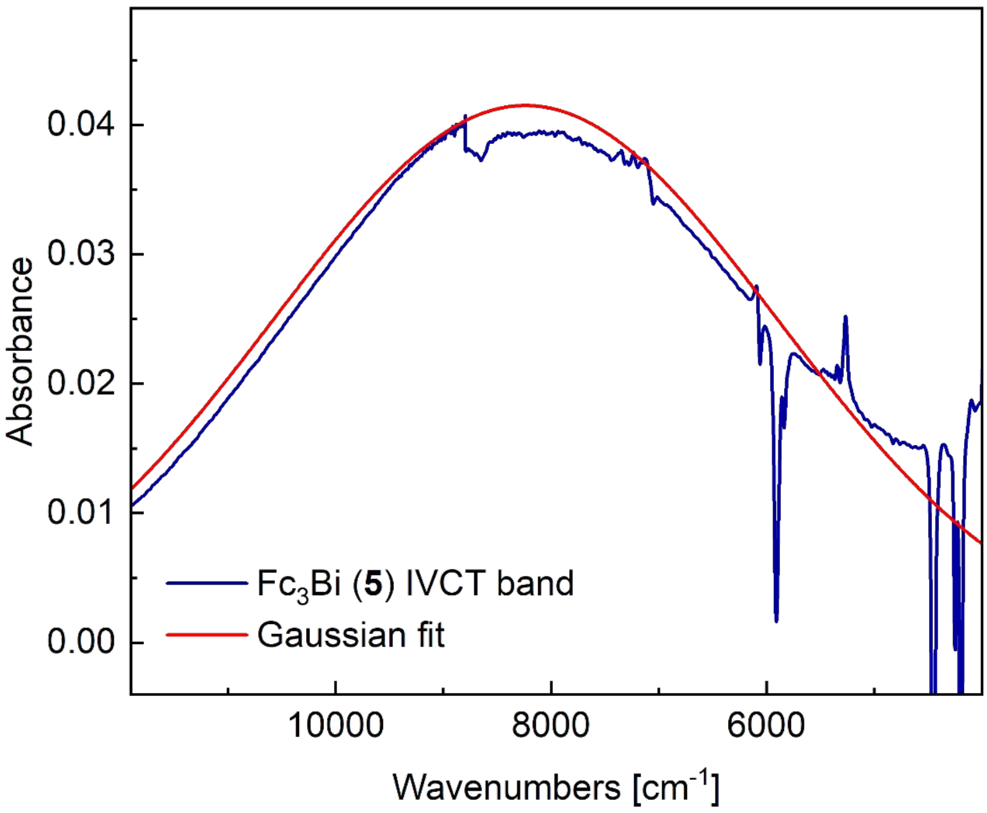

Figure S58. IVCT band of Fc_3Bi (**5**) t = 2400 s during bulk oxidation (applied voltage of 0.60 V) and the Gaussian fit. Estimated ε_{IVCT} = 100 L*mol^{-1} *cm^{-1} (negative bands due to CH_2Cl_2 overtones).

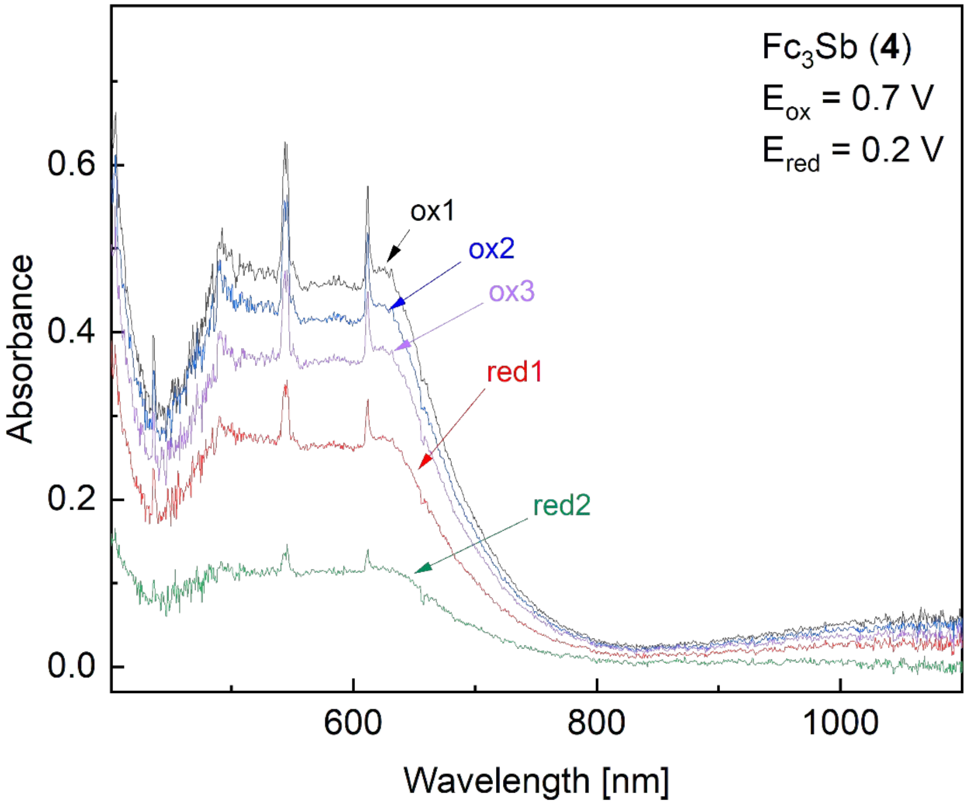

Figure S59. UV-vis absorption spectra of Fc_3Sb (**4**), 1mM, at different times during bulk electrolysis. (ox1 = 320 s, red1 = 400 s, ox2 = 225 s, red2 = 1000s and ox3 = 300 s; applied voltage of 0.7 V for the first oxidation, 0.2 V for the reduction).

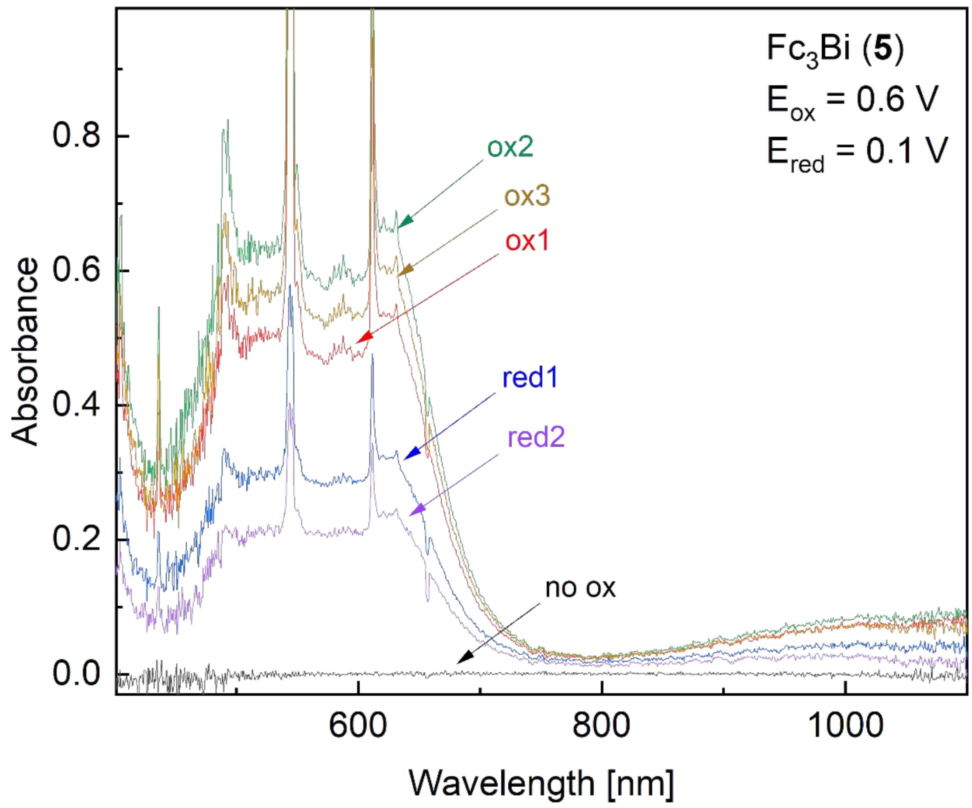

Figure S60. UV-vis absorption spectra of Fc_3Bi (**5**), 1mM, at different times during bulk electrolysis. (ox1 = 30 s, red1 = 400 s, ox2 = 200 s, red2 = 700s and ox3 = 160 s; applied voltage of 0.6 V for the first oxidation, 0.1 V for the reduction).

Crystallographic data

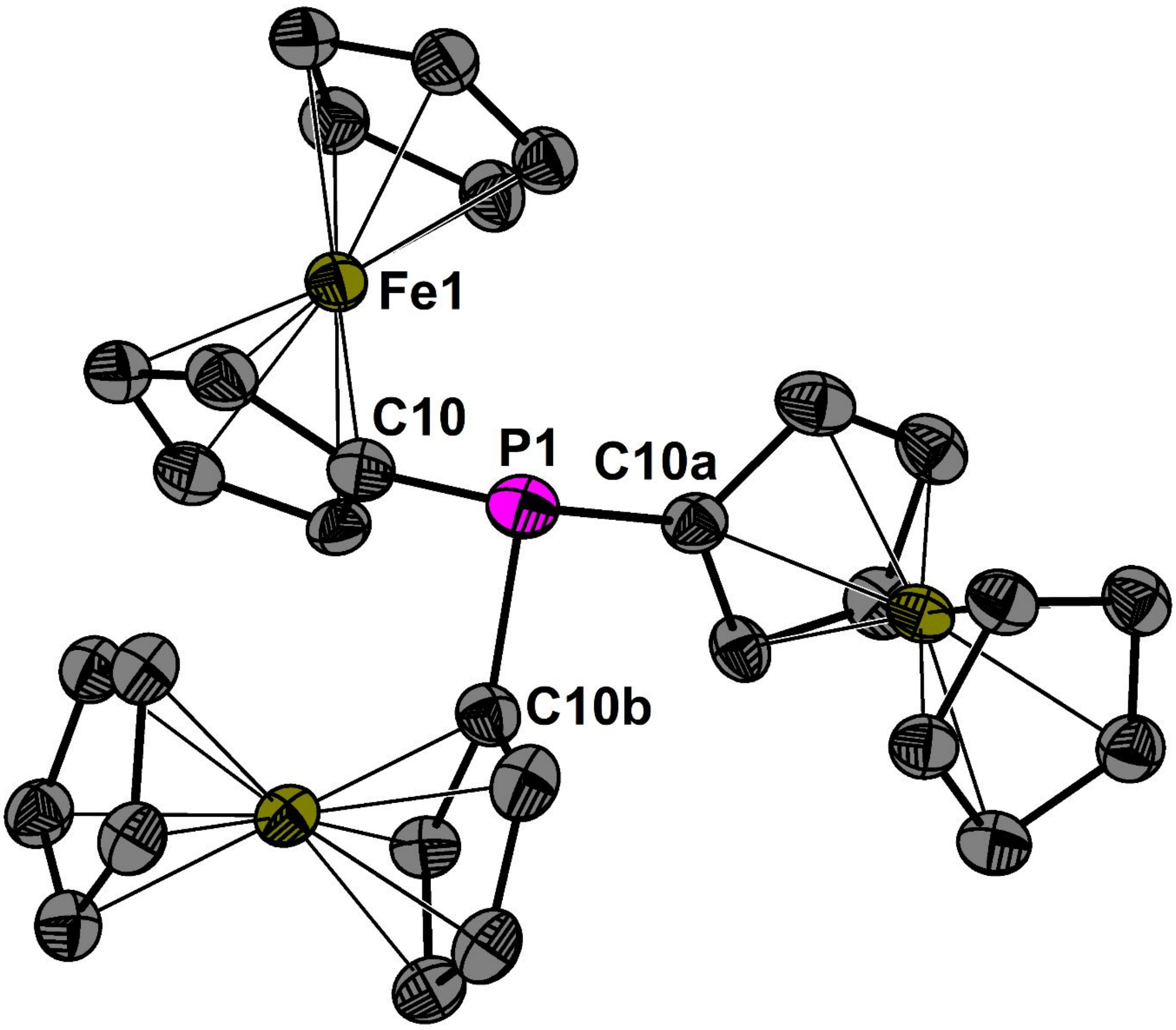

Figure S61. Molecular structure of Fc_3P (**2**) showing 50% probability ellipsoids and the atomic numbering scheme. Hydrogen atoms are omitted for clarity.

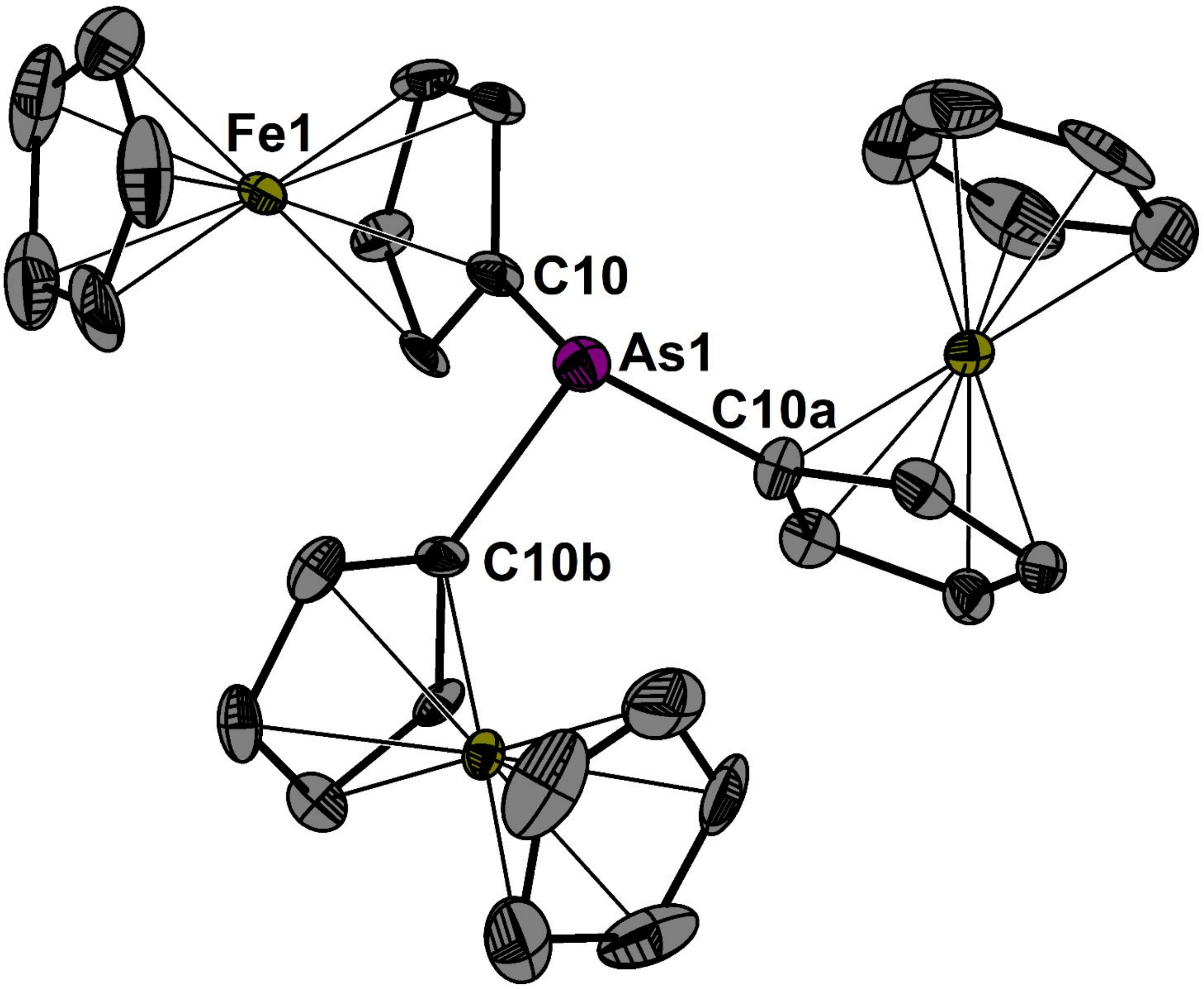

Figure S62. Molecular structure of Fc_3As (**3**) showing 50% probability ellipsoids and the atomic numbering scheme. Hydrogen atoms are omitted for clarity.

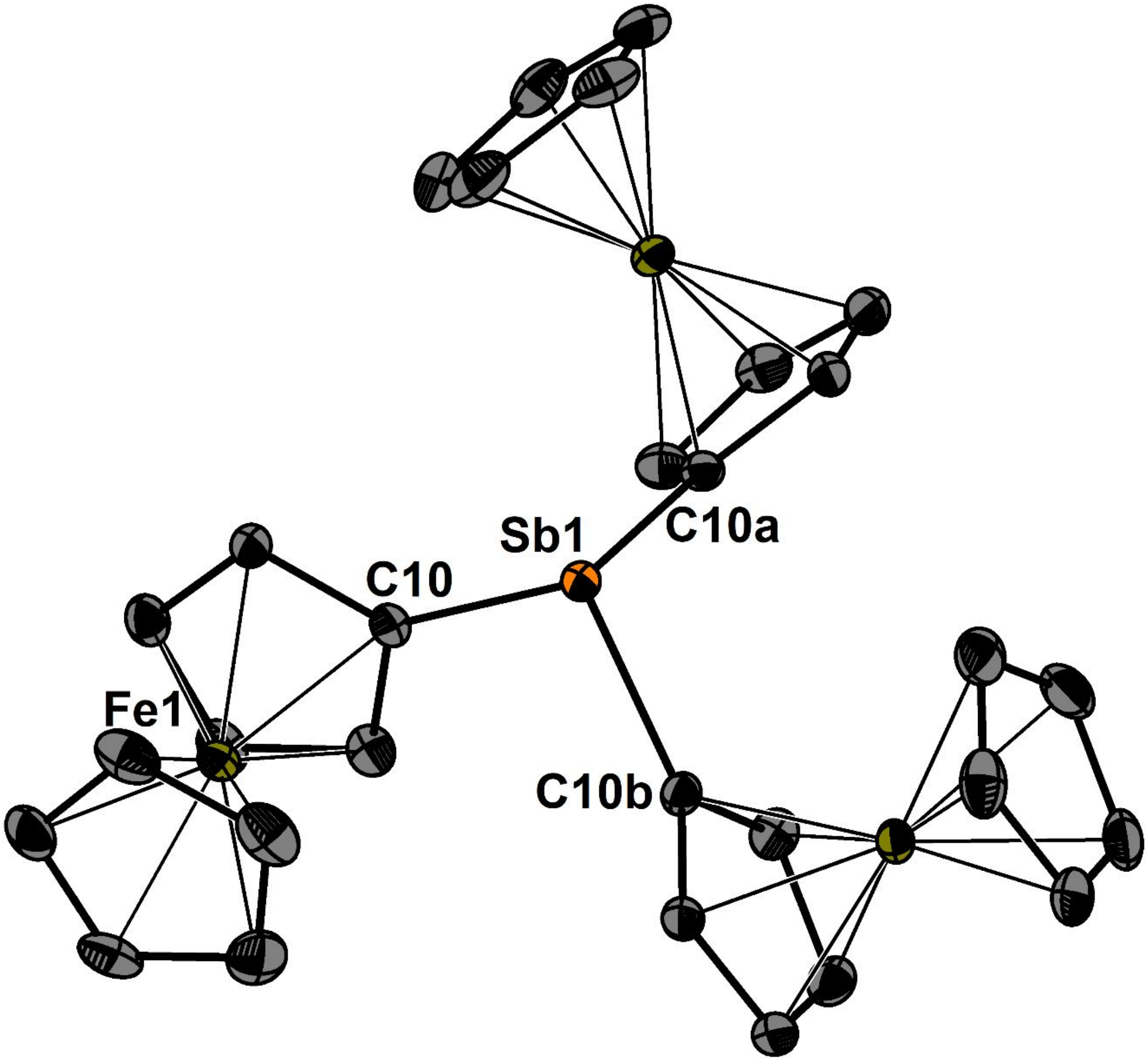

Figure S63. Molecular structure of Fc_3Sb (**4**) showing 50% probability ellipsoids and the atomic numbering scheme. Hydrogen atoms are omitted for clarity.

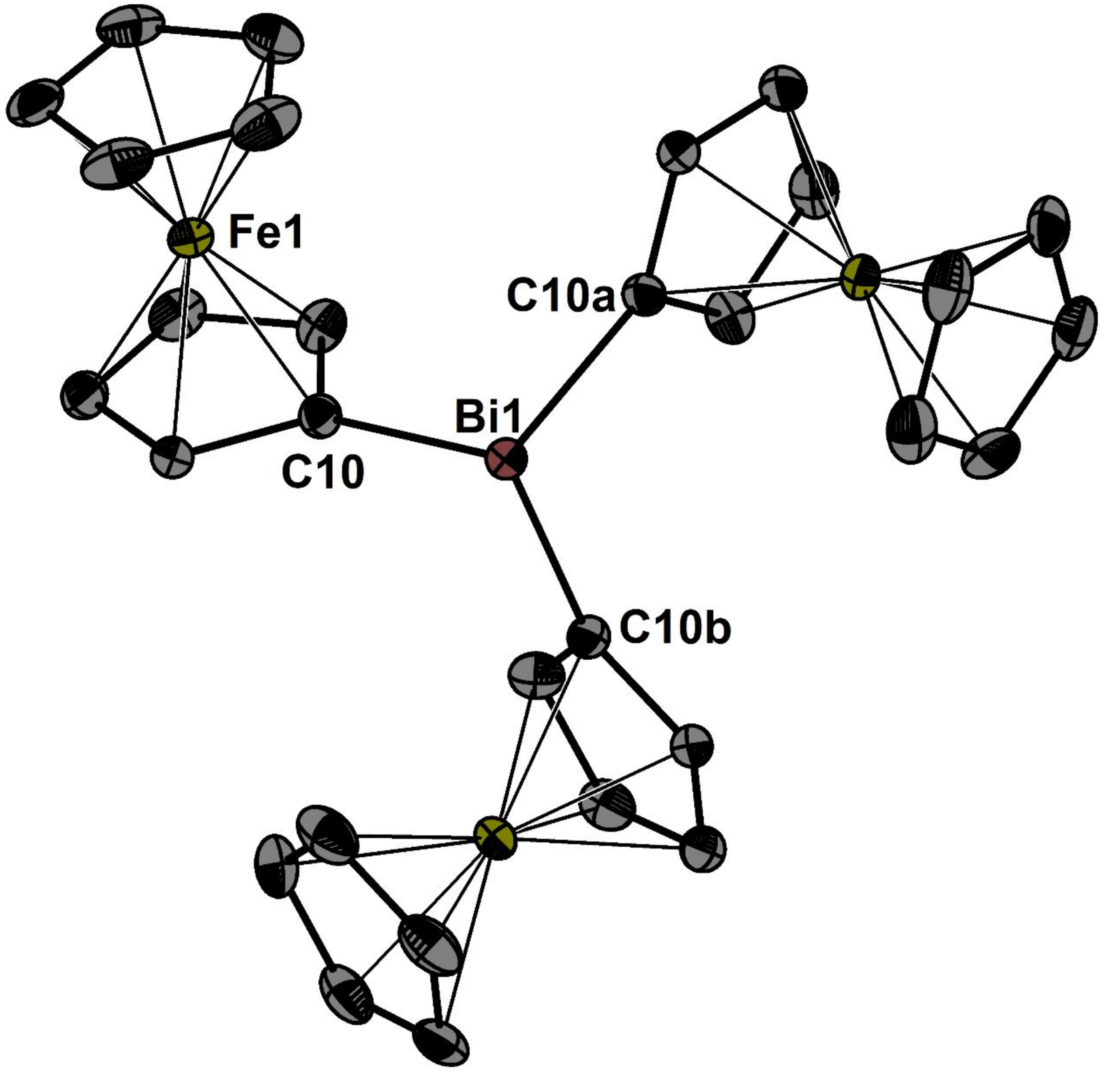

Figure S64. Molecular structure of Fc_3Bi (**5**) showing 50% probability ellipsoids and the atomic numbering scheme. Hydrogen atoms are omitted for clarity.

Table S1. Crystal data and structure refinement of Fc_3P and Fc_3As.

	**$Fc_3P \cdot 0.5\ CH_2Cl_2$**	**$Fc_3As \cdot 0.5\ CHCl_3$**
Formula	$C_{30.50}H_{28}ClFe_3P$	$C_{30.50}H_{27.5}AsCl_{1.5}Fe_3$
Formula weight, g mol^{-1}	628.50	689.68
Crystal system	trigonal	trigonal
Crystal size, mm	0.33 × 0.27 × 0.26	0.25 × 0.23 × 0.22
Space group	$P\bar{3}$	$P\bar{3}$
a, Å	15.7061(10)	15.8312(3)
b, Å	15.7061(10)	15.8312(3)
c, Å	5.8170(5)	5.8490(2)
α, °	90	90
β, °	90	90
γ, °	120	120
V, Å^3	1242.70(19)	1269.52(6)
Z	2	2
Temperature, K	100	100
ρ_{calcd}, Mg m^{-3}	1.680	1.648
μ (Mo *Kα*), mm^{-1}	15.592	3.001
F(000)	642	636
θ range, deg	3.24 to 75.57	2.97 to 24.96
Index ranges	$-19 \le h \le 18$	$-20 \le h \le 20$
	$-19 \le k \le 19$	$-20 \le k \le 20$
	$-7 \le l \le 7$	$-7 \le l \le 7$
No. of reflns collected	12408	28578
Completeness to θ_{max}	99.9%	99.9%
No. indep. Reflns	1576	1483
No. obsd reflns with($I>2\sigma(I)$)	1711	1452
No. refined params	149	177
GooF (F^2)	1.103	1.147
R_1 (*F*) ($I > 2\sigma(I)$)	0.0726	0.0747
wR_2 (F^2) (all data)	0.1910	0.1907
Largest diff peak/hole, e Å^{-3}	0.899 / –0.497	2.745 / –1.837
CCDC number	2352723	2352724

Table S2. Crystal data and structure refinement of Fc_3Sb and Fc_3Bi.

	**Fc_3Sb**	**Fc_3Bi**
Formula	$C_{30}H_{27}Fe_3Sb$	$C_{30}H_{27}BiFe_3$
Formula weight, g mol^{-1}	676.81	764.04
Crystal system	trigonal	trigonal
Crystal size, mm	0.40 × 0.15 × 0.15	0.40 × 0.08 × 0.07
Space group	R3c	R3c
a, Å	18.7256(9)	18.8537(10)
b, Å	18.7256(9)	18.8537(10)
c, Å	12.0721(14)	12.0373(18)
α, °	90	90
β, °	90	90
γ, °	120	120
V, Å^3	3665.9(6)	3705.6(7)
Z	6	6
ρ_{calcd}, Mg m^{-3}	1.839	2.054
μ (Mo *Kα*), mm^{-1}	2.856	8.846
F(000)	2016	2208
θ range, deg	2.31 to 30.06	2.16 to 33.13
Index ranges	$-28 \le h \le 28$	$-28 \le h \le 28$
	$-27 \le k \le 27$	$-28 \le k \le 28$
	$-18 \le l \le 18$	$-18 \le l \le 18$
No. of reflns collected	32986	34007
Completeness to θ_{max}	99.9%	99.7%
No. indep. Reflns	3129	3123
No. obsd reflns with ($I>2\sigma(I)$)	3041	2917
No. refined params	104	104
GooF (F^2)	1.066	1.056
R_1 (*F*) ($I > 2\sigma(I)$)	0.0175	0.0206
wR_2 (F^2) (all data)	0.0415	0.0490
Largest diff peak/hole, e Å^{-3}	0.614 / –0.370	1.481 / –1.110
CCDC number	2352725	2352726

(TD)-DFT Computations

Table S3. TD-DFT computed excited states.

	**Fc_3N**	**Fc_3P**	**Fc_3As**	**Fc_3Sb**	**Fc_3Bi**
Ex. State	E [eV] and f	E [eV] and f	E [eV] and f	E [eV] and f	E [eV] and f
S_1	2.4972<br>0.0000	2.5483<br>0.0000	2.5627<br>0.0000	2.5590<br>0.0002	2.5555<br>0.0002
S_2	2.4976<br>0.0001	2.5484<br>0.0000	2.5629<br>0.0001	2.5592<br>0.0002	2.5559<br>0.0002
S_3	2.4982<br>0.0000	2.5485<br>0.0000	2.5635<br>0.0001	2.5596<br>0.0000	2.5567<br>0.0000
S_4	2.4986<br>0.0001	2.5503<br>0.0003	2.5635<br>0.0002	2.5600<br>0.0001	2.5571<br>0.0000
S_5	2.4993<br>0.0000	2.5503<br>0.0003	2.5641<br>0.0001	2.5603<br>0.0001	2.5575<br>0.0000
S_6	2.5018<br>0.0000	2.5541<br>0.0000	2.5665<br>0.0000	2.5618<br>0.0000	2.5580<br>0.0000
S_7	2.7464<br>0.0000	2.7597<br>0.0002	2.7692<br>0.0001	2.7547<br>0.0002	2.7491<br>0.0002
S_8	2.7469<br>0.0000	2.7597<br>0.0002	2.7696<br>0.0001	2.7549<br>0.0002	2.7493<br>0.0002
S_9	2.7476<br>0.0000	2.7604<br>0.0000	2.7702<br>0.0000	2.7551<br>0.0000	2.7496<br>0.0001
S_{10}	2.7580<br>0.0002	2.7750<br>0.0000	2.7818<br>0.0000	2.7761<br>0.0000	2.7734<br>0.0000
S_{11}	2.7587<br>0.0002	2.7751<br>0.0000	2.7822<br>0.0000	2.7763<br>0.0000	2.7738<br>0.0000
S_{12}	2.7593<br>0.0001	2.7759<br>0.0001	2.7832<br>0.0001	2.7775<br>0.0001	2.7750<br>0.0001
S_{13}	3.5458<br>0.0000	3.5532<br>0.0001	3.5713<br>0.0000	3.5646<br>0.0000	3.5630<br>0.0000
S_{14}	3.5462<br>0.0000	3.5533<br>0.0001	3.5719<br>0.0000	3.5648<br>0.0000	3.5632<br>0.0000
S_{15}	3.5470<br>0.0000	3.5534<br>0.0000	3.5724<br>0.0000	3.5651<br>0.0000	3.5634<br>0.0000
S_{16}	3.5512<br>0.0000	3.5931<br>0.0000	3.5963<br>0.0000	3.5834<br>0.0000	3.5750<br>0.0000
S_{17}	3.5518<br>0.0000	3.5931<br>0.0000	3.5965<br>0.0000	3.5836<br>0.0000	3.5754<br>0.0000
S_{18}	3.5536<br>0.0000	3.5934<br>0.0000	3.5970<br>0.0000	3.5838<br>0.0000	3.5759<br>0.0000
S_{19}	4.5579<br>0.0001	4.6552<br>0.0044	4.7395<br>0.0002	4.5686<br>0.0005	4.5070<br>0.0005
S_{20}	4.5739<br>0.0060	4.6559<br>0.0044	4.7407<br>0.0004	4.5694<br>0.0004	4.5077<br>0.0005

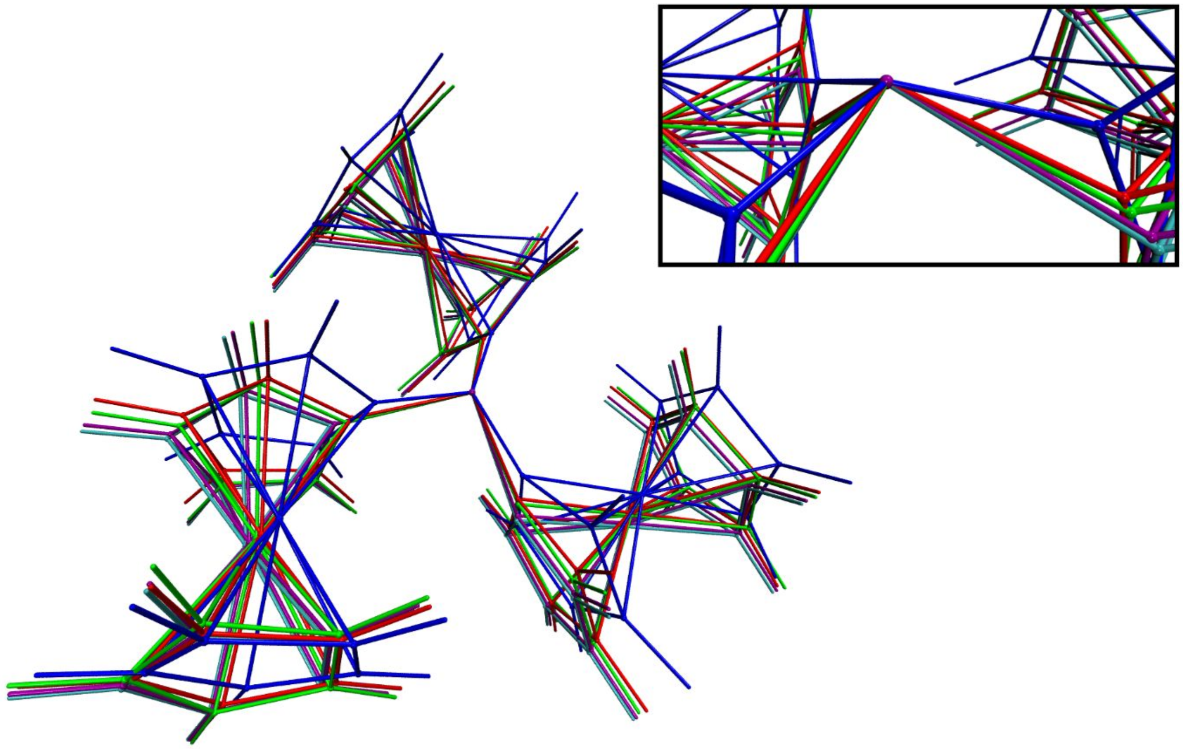

Figure S63. Superposition of gas-phase optimized geometries of Fc_3N (blue), Fc_3P (red), Fc_3As (green), Fc_3Sb (purple) and Fc_3Bi (cyan).

References

[1] D. A. Khobragade, S. G. Mahamulkar, L. Pospíšil, I. Císařová, L. Rulíšek, U. Jahn, *Chem. Eur. J.*, **2012**, *18*: 12267–12277.

[2] D. Miesel, A. Hildebrandt, T. Rüffer, D. Schaarschmidt, H. Lang, *Eur. J. Inorg. Chem.*, **2014**, 2014: 5541–5553.

[3] R. J. LeSuer, C Buttolph, W. E. Geiger, *Anal. Chem.* **2004** *76*, 6395–6401.

[4] G. R. Fulmer, A. J. M. Miller, N. H. Sherden, H. E. Gottlieb, A. Nudelman, B. M. Stoltz, J. E. Bercaw, K. I. Goldberg, *Organometallics*, **2010**, *29*, 2176–2179.

[5] F. Menges, ‘‘Spectragryph – optical spectroscopy software‘‘, Version 1.2.12, **2022**, http://www.effemm2.de/spectragryph/ (accessed 2024-02-14)
